# Supplementary material for: A GWAS in Latin Americans highlights the convergent evolution of lighter skin pigmentation in Eurasia
Source: Nat Commun. 2019 Jan 21;10:358. doi: 10.1038/s41467-018-08147-0 (PMC6341102; doi:10.1038/s41467-018-08147-0)
Supplement: Supplementary file 1 — Supplementary Information [file 41467_2018_8147_MOESM1_ESM.pdf]

Supplementary materials for

A GWAS in Latin Americans highlights the convergent evolution of lighter skin pigmentation in Eurasia

TABLE OF CONTENTS

Supplementary Tables 5
Supplementary Table 1: Features of the study sample 5
Supplementary Table 2: Correlation of pigmentation traits and covariates 6
A) Correlation between the pigmentation traits reported 6
B) Correlation between pigmentation traits and covariates 7
C) Correlation between quantitative eye color variables from different color models 8
Supplementary Table 3: Heritability (h^2) estimates for the pigmentation traits 9
Supplementary Table 4: Tail Strength (TS) statistic 10
Table 4A: Tail Strength (TS) statistic and genomic Inflation Factor (λ) from the GWAS for pigmentation traits in the CANDELA sample 10
Table 4B: Tail Strength (TS) statistic and genomic Inflation Factor (λ) from previously published GWAS in the CANDELA sample 11
Table 4C: Tail Strength (TS) statistic and genomic Inflation Factor (λ) from other published GWAS studies 13
Supplementary Table 5: Conditional GWAS results. 15
Supplementary Table 6: Multivariate association analysis combining all pigmentation traits. 17
Supplementary Table 7: Allele frequencies of the derived allele for index SNPs 19
Supplementary Table 8: Signals of selection at index SNPs in Eurasian populations. 20
Supplementary Table 9: Enrichment of signatures of positive selection at genomic regions associated with pigmentation traits. 21
A) 21
B) 21
Supplementary Table 10: Worldwide populations included in the correlation analysis between the derived allele frequency at pigmentation loci and solar radiation. 22
Supplementary Table 11: Correlation between allele frequency at index SNPs and solar radiation. 25
Supplementary Table 12: List of reported GWAS associations with pigmentation. 26
Supplementary Table 13: Proportion of trait variation explained by each SNP 30

Supplementary Figures 31
Supplementary Figure 1: Eye landmarking protocol 31
Supplementary Figure 2: Digital eye color extraction 33
Supplementary Figure 3: Color spaces used in the eye pigmentation analysis 37
RGB Space: 37
RGB Principal Components space: 38
HSV color space: 39
HCL color space: 41
Lab color space: 43
Projection Length: 45
Supplementary Figure 4: Frequency distribution of quantitative eye color variables 47
A) Histogram of L (Brightness) 47
B) Histogram of C (Saturation) 48
C) Histogram of cos(H) (Hue) 49
Supplementary Figure 5: Continental ancestry in the CANDELA sample 52
Supplementary Figure 6: Selection of genetic Principal Components for inclusion in the GWAS analyses 54
A) Scree plot 54

B) GWAS Q-Q plots 55
Supplementary Figure 7: Regional association plots of genome-wide significant SNPs associated to pigmentation traits 57
A) rs16891982 – 5p13 (SLC45A2) – Skin pigmentation (MI) 57
B) rs12203592 – 6p25 (IRF4) – L (Brightness) 57
C) rs10809826 – 9p23 (TYRP1) – L (Brightness) 58
D) rs7118677 – 11q14 (GRM5) – Skin pigmentation (MI) 58
E) rs1042602 – 11q14 (TYR) – Skin pigmentation (MI) 59
F) rs1126809 – 11q14 (TYR) – Skin pigmentation (MI) 59
G) rs4778219 – 15q13 (OCA2) – L (Brightness) 60
H) rs1800407 – 15q13 (OCA2) – L (Brightness) 60
I) rs1800404 – 15q13 (OCA2) – Skin pigmentation (MI) 61
J) rs12913832 – 15q13 (HERC2) – L (Brightness) 61
K) rs4778249 – 15q13 (HERC2) – C (Saturation) 62
L) rs1426654 – 15q21 (SLC24A5) – Skin pigmentation (MI) 62
M) rs885479 – 16q24 (MC1R) – Skin pigmentation (MI) 63
Supplementary Figure 8: Meta-analysis for 6 index SNPs representing novel associations to pigmentation traits 64
Supplementary Figure 9: Genomic annotation in the 10q26 intergenic region around rs11198112 65
Supplementary Figure 10: Worldwide allele frequency of rs11198112 67
Supplementary Figure 11: Worldwide allele frequencies of rs2240751 (MFSD12) 68
Supplementary Figure 12: Cross-tissue expression of novel candidate genes of pigmentation traits in the GTEx database 69
A) 1q32 DSTYK 69
B) 10q26 EMX2 70
C) 19p13 MFSD12 70
D) 20q13 WFDC5 71
E) 22q12 MPST 71
Supplementary Figure 13: Distribution of selection statistics scores surrounding index SNPs 72
A) 1q32 – rs3795556 73
B) 5p13 – rs16891982 74
C) 6p25 – rs12203592 75
D) 9p23 – rs10809826 76
E) 10q26 – rs11198112 77
F) 11q14 – rs1042602 78
G) 11q14 – rs1126809 79
H) 11q14 – rs7118677 80
I) 15q13 – rs1800404 81
J) 15q13 – rs1800407 82
K) 15q13 – rs4778219 83
L) 15q13 – rs4778249 84
M) 15q13 – rs12913832 85
N) 15q21 – rs1426654 86
O) 16q24 - rs885479 87
P) 19p13 – rs2240751 88
Q) 20q13 – rs17422688 89

R) 22q12 – rs5756492 90
Supplementary Figure 14: RMSE plots. **91**
Supplementary Figure 15: Estimation of the start of selection and selection coefficient at the *MFSD12* gene region **92**
Supplementary Figure 16: Joint estimation start of the start of selection and selection coefficient at the *MFSD12* gene region **93**
Supplementary Figure 17: Histogram of Melanin Index measurement variability **94**
Supplementary Figure 18: Conversion of Melanin Index into approximate skin color **95**

Supplementary Notes **96**
Supplementary Note 1: Coalescent simulations for the Out-of-Africa model **96**

REFERENCES **97**

SUPPLEMENTARY TABLES

Supplementary Table 1: Features of the study sample

	Total	Colombia	Brazil	Chile	Mexico	Peru
Sample size	6357	1507	651	1745	1207	1247
Percentage	100	23.7	10.2	27.5	19	19.6
% Female	54	55.9	68.5	39.6	60.3	58.4
Age (years)						
Min	18	18	18	18	18	18
Mean	24.2	24	25.8	25.2	24.4	22.2
Max	45	40	45	45	44	44
S.D.	5.7	5.3	6.3	5.8	5.6	5.2
Age, for Males (years)						
Min	18	18	18	18	18	18
Mean	24.9	24.7	25.8	25.3	25.1	23
Max	45	40	45	45	44	44
S.D.	5.7	5.5	6.4	5.5	5.6	5.7
Age, for Females (years)						
Min	18	18	18	18	18	18
Mean	23.8	23.5	25.4	25.2	24	21.6
Max	45	40	44	45	41	42
S.D.	5.7	5	4.2	6.2	4.7	4.7

Supplementary Table 2: Correlation of pigmentation traits and covariates

A) Correlation between the pigmentation traits reported

Correlation values are presented in the lower left triangle while corresponding P-values are presented in the upper right triangle. Negative correlation values are represented in red color while positive correlation values are represented in green color. Higher magnitude of correlation is represented by darker intensity of colors. From a total of 6,357 volunteers with phenotype & genotype data, sample size for the quantitative eye color traits is 5513.

	Skin color (MI)	Hair color (cat)	Eye color (cat)	L (Brightness)	C (Saturation)	cos(H) (Hue)
Skin color (MI)		0	0	0	0	0
Hair color (categorical)	0.30		0	0	0	0
Eye color (categorical)	0.31	0.50		0	0	0
L (Brightness)	-0.35	-0.46	-0.78		0	0
C (Saturation)	-0.20	-0.05	-0.08	0.34		0
cos(H) (Hue)	0.10	0.24	0.40	-0.39	0.23	

Hue (H) values were standardized prior to the cosine transformation by subtracting its median, 20°.

B) Correlation between pigmentation traits and covariates

Correlation coefficient:

			Ancestry			Genetic PCs					
	Age	Sex	European	Native American	African	PC1	PC2	PC3	PC4	PC5	PC6
Skin color (MI)	-0.05	0.03	-0.47	0.40	0.19	-0.46	0.13	-0.04	0.15	0.01	-0.03
Hair color (categorical)	-0.01	-0.10	-0.38	0.34	0.08	-0.35	0.21	-0.10	0.12	-0.11	-0.01
Eye color (categorical)	-0.08	0.00	-0.43	0.39	0.07	-0.39	0.20	-0.08	0.12	-0.06	0.00
L (Brightness)	0.14	-0.07	0.48	-0.43	-0.13	0.44	-0.29	0.17	0.04	-0.02	-0.01
C (Saturation)	0.07	-0.05	0.24	-0.23	0.01	0.24	-0.03	-0.11	0.08	-0.22	0.01
cos(H) (Hue)	-0.06	0.00	-0.20	0.18	0.07	-0.18	0.18	-0.05	0.06	-0.14	0.03

Corresponding P-values:

	Age	Sex	European	Native American	African	PC1	PC2	PC3	PC4	PC5	PC6
Skin color (MI)	0	0.03	0	0	0	0	0	0	0	0.32	0.01
Hair color (categorical)	0.36	0	0	0	0	0	0	0	0	0	0.46
Eye color (categorical)	0	0.85	0	0	0	0	0	0	0	0	0.98
L (Brightness)	0	0	0	0	0	0	0	0	0.01	0.23	0.51
C (Saturation)	0	0	0	0	0.59	0	0.01	0	0	0	0.45
cos(H) (Hue)	0	0.79	0	0	0	0	0	0	0	0	0.05

Native American, European and African continental ancestry estimates were obtained from ADXMITURE (Supplementary Figure 5). Sex was coded as female = 0 and male = 1. Negative correlation values are represented in red color while positive correlation values are represented in green color. Higher magnitude of correlation is represented by darker intensity of colors.

C) Correlation between quantitative eye color variables from different color models

A detailed description of the quantitative eye color phenotyping is presented in the Supplementary Figures 1-3. Correlation values are presented in the lower left triangle while corresponding P-values are presented in the upper right triangle. Negative correlation values are represented in red color while positive correlation values are represented in green color. Higher magnitude of correlation is represented by darker intensity of colors.

	RGB			PC of RGB		HCL			HSV		CIE Lab			Hue standardized		
	R	G	B	PC1	PC2	H	C	L	S	V	L	a	b	cos(H)	sin(H)	Proj. Len.
R		0.00	0.00	0.00	0.00	0.00	0.00	0.00	0.00	0.00	0.00	0.00	0.00	0.00	0.00	0.00
G	0.82		0.00	0.00	0.00	0.00	0.00	0.00	0.00	0.00	0.00	0.00	0.00	0.00	0.00	0.00
B	0.54	0.90		0.00	0.00	0.00	0.00	0.00	0.00	0.00	0.00	0.00	0.00	0.00	0.00	0.00
PC1	0.90	0.98	0.84		1.00	0.00	0.00	0.00	0.00	0.00	0.00	0.00	0.00	0.00	0.00	0.00
PC2	0.43	-0.15	-0.53	0.00		0.00	0.00	0.61	0.00	0.00	0.00	0.00	0.00	0.00	0.00	0.58
H	0.19	0.43	0.53	0.38	-0.37		0.00	0.00	0.00	0.00	0.00	0.00	0.00	0.00	0.00	0.00
C	0.70	0.19	-0.21	0.34	0.93	-0.18		0.00	0.00	0.00	0.00	0.00	0.00	0.00	0.03	0.00
L	0.91	0.97	0.84	1.00	0.01	0.38	0.34		0.00	0.00	0.00	0.00	0.00	0.00	0.00	0.00
S	-0.26	-0.67	-0.81	-0.58	0.61	-0.43	0.40	-0.57		0.00	0.00	0.00	0.00	0.00	0.00	0.00
V	1.00	0.83	0.56	0.91	0.40	0.22	0.69	0.92	-0.28		0.00	0.00	0.00	0.00	0.00	0.00
L	0.94	0.96	0.78	0.99	0.10	0.35	0.43	0.99	-0.52	0.95		0.00	0.00	0.00	0.00	0.00
a	0.29	-0.30	-0.59	-0.13	0.94	-0.35	0.84	-0.11	0.70	0.27	-0.04		0.00	0.00	0.00	0.00
b	0.69	0.20	-0.23	0.33	0.93	-0.25	0.98	0.33	0.38	0.67	0.42	0.79		0.00	0.00	0.00
cos(H)	-0.15	-0.42	-0.58	-0.38	0.46	-0.69	0.23	-0.39	0.35	-0.20	-0.34	0.37	0.34		0.00	0.00
sin(H)	0.46	0.71	0.64	0.64	-0.25	0.23	-0.03	0.62	-0.56	0.47	0.63	-0.44	0.04	-0.27		0.00
Proj. Len.	0.91	0.97	0.84	1.00	0.01	0.39	0.34	1.00	-0.57	0.92	0.99	-0.11	0.33	-0.39	0.62	

Abbreviations: RGB: Red, Green, and Blue; HCL: Hue, Chroma, and Lightness; HSV: Hue, Saturation, and Value; Lab: Lightness, a and b color components. Hue (H) values were standardized prior to sine & cosine transformations by subtracting its median, 20°. Projected length represents T-index measure Beleza, Johnson (1).

Supplementary Table 3: Heritability (h^2) estimates for the pigmentation traits

Trait	Heritability	S.E.	P-value
Skin color (MI)	0.85	0.05	0.00
Hair color (categorical)	1.00	0.05	0.00
Eye color (categorical)	1.00	0.06	0.00
cosH (Hue)	0.84	0.06	0.00
C (Saturation)	0.79	0.06	0.00
L (Brightness)	1.00	0.06	0.00

Supplementary Table 4: Tail Strength (TS) statistic

Table 4A: Tail Strength (TS) statistic and genomic Inflation Factor (λ) from the GWAS for pigmentation traits in the CANDELA sample

Trait	TS	λ
Skin pigmentation (MI)	0.08	1.11
Hair color (categorical)	0.04	1.05
Eye color (categorical)	0.05	1.07
L (Brightness)	0.05	1.07
C (Saturation)	0.03	1.04
cosH (Hue)	0.05	1.05

Note: All the TS statistics shown above are significantly different from zero. Results are based on the conditional GWAS.

Tables 4B presents TS and Lambda results for previously published CANDELA GWAS studies. Though based on the same cohort and employing nearly identical set of samples and PCs to adjust for substructure, the TS statistic varies considerably, having low values for ear traits which didn't produce any genome-wide significant hits, and having the highest value (comparable to pigmentation traits here) for hair shape which had a large number of significant SNPs in long LD blocks around the index SNPs.

Table 4C presents TS & Lambda results for other published GWAS studies whose summary statistics are publicly available. Again TS values vary considerably within the same study, having highest values for pigmentation traits, height and BMI.

Only autosomal SNPs were used in Tables 4B-C as sex chromosome results were not available for some studies.

In all studies, the TS statistic is generally higher for traits with more significantly associated SNPs. That it is very close to zero for some traits in the CANDELA sample that show few or no associations indicates that the distributional assumption under the null is indeed correct, and that there is no inherent substructure remaining in the dataset after controlling with the genetic PCs.

Table 4B: Tail Strength (TS) statistic and genomic Inflation Factor (λ) from previously published GWAS in the CANDELA sample

The table below lists results from three previously published CANDELA studies on ear, face and hair traits. Number of genome-wide significant SNPs is shown (not pruned for LD). Only autosomal SNPs are used.

Trait	Trait type	Population	Gwas method	Sample size	Total SNPs	Significant SNPs	Lambda	TS	Source
Cheekbone Protrusion	Quantitative	Latin Americans	Single GWAS	6,275	670,261	0	1.0180	0.0234	(2)
Chin Protrusion	Quantitative	Latin Americans	Single GWAS	6,275	670,261	0	1.0068	0.0080	(2)
Chin Shape	Quantitative	Latin Americans	Single GWAS	6,275	670,261	0	1.0130	0.0170	(2)
Columella Inclination	Quantitative	Latin Americans	Single GWAS	6,275	670,261	4	1.0153	0.0219	(2)
Forehead Bias	Binary	Latin Americans	Single GWAS	6,275	670,143	0	1.0189	0.0203	(2)
Lower Lip	Quantitative	Latin Americans	Single GWAS	6,275	670,261	0	1.0235	0.0330	(2)
Mouth Corner Orientation	Quantitative	Latin Americans	Single GWAS	6,275	670,261	0	1.0035	0.0078	(2)
Nasal Root	Binary	Latin Americans	Single GWAS	6,275	670,143	0	1.0108	0.0146	(2)
Nose Bridge Breadth	Quantitative	Latin Americans	Single GWAS	6,275	670,261	7	1.0077	0.0111	(2)
Nose Profile	Quantitative	Latin Americans	Single GWAS	6,275	670,261	0	1.0106	0.0113	(2)
Nose Protrusion	Quantitative	Latin Americans	Single GWAS	6,275	670,261	1	1.0199	0.0274	(2)
Nose Tip Shape	Quantitative	Latin Americans	Single GWAS	6,275	670,261	0	1.0082	0.0103	(2)
Nose Wing Breadth	Quantitative	Latin Americans	Single GWAS	6,275	670,261	3	1.0199	0.0277	(2)
Upper Lip	Quantitative	Latin Americans	Single GWAS	5,062	670,261	0	1.0187	0.0274	(2)
Antitragus size	Quantitative	Latin Americans	Single GWAS	5,062	595,536	1	1.0103	0.0150	(3)
Crus helix expression	Quantitative	Latin Americans	Single GWAS	5,062	595,536	1	1.0103	0.0150	(3)
Darwin's tubercle	Quantitative	Latin Americans	Single GWAS	5,062	595,536	1	1.0103	0.0150	(3)
Ear Protrusion	Quantitative	Latin Americans	Single GWAS	5,062	670,261	4	1.0242	0.0317	(3)
Fold of antihelix	Quantitative	Latin Americans	Single GWAS	5,062	595,536	1	1.0103	0.0150	(3)
Helix Rolling	Quantitative	Latin Americans	Single GWAS	5,062	595,536	1	1.0103	0.0150	(3)
Lobe Attachment	Quantitative	Latin Americans	Single GWAS	5,062	595,536	1	1.0103	0.0150	(3)
Lobe Size	Quantitative	Latin Americans	Single GWAS	5,062	595,536	1	1.0103	0.0150	(3)
Superior Crus of antihelix expression	Quantitative	Latin Americans	Single GWAS	5,062	595,536	1	1.0103	0.0150	(3)
Tragus size	Quantitative	Latin Americans	Single GWAS	5,062	595,536	1	1.0103	0.0150	(3)
Balding	Quantitative	Latin Americans	Single GWAS	6,630	691,403	1	1.0300	0.0418	(3)
Beard density	Quantitative	Latin Americans	Single GWAS	6,630	667,544	25	1.0115	0.0208	(3)

EyeBrow density	Quantitative	Latin Americans	Single GWAS	6,630	667,531	3	1.0151	0.0210	(4)
Hair graying	Quantitative	Latin Americans	Single GWAS	6,630	691,403	1	1.0139	0.0191	(4)
Hair shape	Quantitative	Latin Americans	Single GWAS	6,630	691,403	618	1.0381	0.0629	(4)
Head Shape	Quantitative	Latin Americans	Single GWAS	5,062	670,261	0	0.9970	0.0003	(4)
Mono-Brow	Quantitative	Latin Americans	Single GWAS	6,630	668,298	0	1.0160	0.0229	(4)

Table 4C: Tail Strength (TS) statistic and genomic Inflation Factor (λ) from other published GWAS studies

The table below lists results from other published GWAS results from various sources.

Trait	Type	Population	Gwas method	Sample size	Total SNPs	Significant SNPs	Lambda	Tail Statistic	Source
Crohn's disease	Binary	European	Meta-analysis	21,389	953,241	995	1.3107	0.1671	(5)
WHR (adjusted for BMI)	Quantitative	European	Meta-analysis	77,167	2,483,325	299	1.0475	0.0237	(6)
Height	Quantitative	European	Meta-analysis	183,727	2,469,635	4,663	1.0645	0.0909	(7)
Age at menarche	Quantitative	European	Single GWAS	459,327	12,006,186	31,311	1.4295	0.2290	(8)
Age at menopause	Quantitative	European	Single GWAS	459,327	11,991,424	7,666	1.1474	0.0924	(8)
Body mass index (BMI)	Quantitative	European	Single GWAS	459,327	12,007,571	74,325	1.7665	0.3456	(8)
Hair color	Quantitative	European	Single GWAS	459,327	12,008,523	68,728	1.2544	0.1866	(8)
Height	Quantitative	European	Single GWAS	459,327	12,007,535	306,420	2.1738	0.4244	(8)
Skin color	Quantitative	European	Single GWAS	459,327	12,006,891	33,063	1.2544	0.1563	(8)
Sunburn	Quantitative	European	Single GWAS	459,327	12,010,706	12,170	1.1999	0.1311	(8)
Tanning	Quantitative	European	Single GWAS	459,327	12,007,147	28,872	1.1999	0.1428	(8)
Type 2 Diabetes	Binary	European	Single GWAS	459,327	12,007,881	3,252	1.1999	0.1150	(8)
Fasting glucose	Quantitative	European	Meta-analysis	51,750	2,628,879	505	1.0728	0.0539	(9)
Fasting insuline	Quantitative	European	Meta-analysis	51,750	2,627,848	34	1.0640	0.0504	(9)
Depression	Quantitative	European	Meta-analysis	161,460	6,524,474	3	1.1021	0.0732	(10)
Neuroticism	Quantitative	European	Meta-analysis	170,911	6,524,432	4,192	1.2264	0.1474	(10)
Body mass index (BMI)	Quantitative	European	Meta-analysis	249,796	2,471,516	772	1.0426	0.0487	(11)
HDL cholesterol	Quantitative	European	Meta-analysis	100,136	2,692,429	2,214	1.0005	0.0220	(12)
LDL cholesterol	Quantitative	European	Meta-analysis	100,136	2,692,564	1,769	1.0000	0.0234	(12)
Total Cholesterol	Quantitative	European	Meta-analysis	100,136	2,692,413	2,593	1.0000	0.0293	(12)
Triglycerides	Quantitative	European	Meta-analysis	100,136	2,692,560	1,808	1.0000	0.0243	(12)
Adenoma Prevention with Celecoxib	Binary	European	Single GWAS	1,406	575,563	5	1.0023	0.0013	(13)
Allopurinol response	Quantitative	European	Single GWAS	2,027	635,721	1	1.0033	0.0034	(14)
Cisplatin toxicity	Quantitative	European	Single GWAS	511	5,060,354	1	1.0431	0.0277	(15)
HDL cholesterol	Quantitative	Primarily European	Meta-analysis	94,595	2,447,441	3,524	1.0150	0.0373	(16)
LDL cholesterol	Quantitative	Primarily European	Meta-analysis	94,595	2,437,751	3,078	1.0155	0.0354	(16)
Total Cholesterol	Quantitative	Primarily European	Meta-analysis	94,595	2,446,981	4,169	1.0141	0.0435	(16)

Triglycerides	Quantitative	Primarily European	Meta-analysis	94,595	2,439,432	3,249	1.0080	0.0312	(16)

Supplementary Table 5: Conditional GWAS results.

Genome-wide significant P-values (< 5E-8) are in the deepest shade of orange. Abbreviations: MI, Melanin Index; L, lightness; C, chroma; H, hue.

Table 5A. Conditional GWAS results for previously well-established index SNPs in genomic regions (from Table 1) associated with pigmentation traits[*].

				Trait/Association (P-value)					
				Skin	Hair	Eye			
Region	SNP	Candidate gene	SNP Annotation	MI	Categorical	Categorical	L (Brightness)	C (Saturation)	cos(H) (Hue)
5p13	rs16891982[a]	*SLC45A2*	F374L	—	—	—	—	1.7E-08	4.5E-05
6p25	rs12203592	*IRF4*	Intronic	—	—	—	—	1.5E-03	8.0E-02
9p23	rs10809826[a]	*TYRP1*	Intergenic	2.2E-03	6.5E-02	—	—	—	7.8E-03
15q13	rs1800404	*OCA2*	Synonymous/TFB	—	4.4E-03	—	—	3.4E-10	2.7E-02
15q13	rs12913832	*HERC2*	Intronic	—	—	—	—	5.2E-07	—
15q21	rs1426654	*SLC24A5*	T111A	—	—	—	—	—	6.7E-01

Table 5B. Other index SNPs in genomic regions (from Table 1) associated with pigmentation traits. Novel genomic regions in bold.

				Trait/Association (P-value)					
				Skin	Hair	Eye			
Region	SNP	Candidate gene	SNP Annotation	MI	Categorical	Categorical	L (Brightness)	C (Saturation)	cos(H) (Hue)
**1q32**	**rs3795556**	***DSTYK***	**3' UTR**	1.5E-01	7.7E-01	8.1E-01	1.8E-08	1.3E-09	2.6E-01
**10q26**	**rs11198112**	***EMX2***	**Intergenic**	7.3E-11	6.6E-01	4.1E-01	7.7E-01	6.0E-01	5.7E-01
11q14	rs1042602	*TYR*	S192Y	1.0E-13	2.5E-09	6.0E-01	3.5E-01	2.2E-02	9.5E-01
16q24	rs885479	*MC1R*	R163Q	4.1E-09	5.5E-02	7.3E-01	8.8E-01	8.5E-01	8.4E-01
**19p13**	**rs2240751**	***MFSD12***	**Y182H**	4.2E-13	9.7E-01	3.0E-01	9.1E-01	1.8E-01	9.9E-01
**20q13**	**rs17422688**	***WFDC5***	**H97Y**	7.2E-01	5.4E-01	9.9E-01	1.9E-01	9.4E-01	3.5E-09
**22q12**	**rs5756492**	***MPST***	**Intronic**	2.3E-03	9.9E-01	8.8E-02	1.0E-02	4.8E-08	2.2E-01

Table 5C. Additional index SNPs in well-established genomic regions (from Table 1) associated with pigmentation traits[†].

				Trait/Association (P-value)					
				Skin	Hair	Eye			
Region	SNP	Candidate gene	SNP Annotation	MI	Categorical	Categorical	L (Brightness)	C (Saturation)	cos(H) (Hue)
11q14	rs7118677[a]	*GRM5*	Intronic	6.1E-12	2.7E-08	4.1E-01	9.7E-01	4.7E-01	5.8E-01
11q14	rs1126809[a]	*TYR*	R402Q	5.7E-10	1.0E-05	2.0E-05	3.2E-08	1.3E-01	6.4E-04
15q13	rs4778219	*OCA2*	Intronic	3.0E-09	4.4E-03	3.7E-07	3.6E-11	7.0E-01	4.8E-01
15q13	rs1800407	*OCA2*	R419Q	4.7E-19	1.8E-04	6.1E-17	1.3E-19	3.7E-08	8.9E-03
15q13	rs4778249[a]	*HERC2*	Intronic	1.3E-03	5.2E-01	3.0E-02	2.8E-09	1.8E-14	1.5E-01

[*] These six SNPs were used to condition the GWAS, indicated by superscript [b] in Table 1. A '—' indicates associations that were significant in Table 1 for a SNP with a trait and was therefore conditioned upon, hence the P-value was not computed in conditioned analysis.

[a] These markers were obtained through imputation. Their imputation quality 'info' metric was ≥ 0.975, the median value being 0.993. The other markers were obtained from chip genotyping, and their 'concordance' metric was > 0.9, the median value being 0.981.

[†] The independence of association signals of these SNPs (indicated by superscript [c] in Table 1) from the main index SNPs in these genomic regions was confirmed by conditioned analyses.

Supplementary Table 6: Multivariate association analysis combining all pigmentation traits.

Multivariate regression analysis, an extension of the single-trait regression performed in GWAS, was performed to check if correlation between pigmentation traits could lead to new or stronger genetic signals. The regression analysis was performed for each SNP to test for association with all pigmentation traits (except categorical eye color) combined while adjusting for age, sex and the first 6 PCs. The method provides a regression coefficient for each pigmentation trait and the vector of coefficient is tested jointly using the Wald test to check if it deviates significantly from zero. The test employed here is nearly identical to the Wald test performed in Zhou and Stephens (17), the only difference being in the use of genetic PCs instead of a genetic kinship matrix. The difference is minor since the PCs are top eigenvectors of the kinship matrix. The Manhattan plot below summarizes the results obtained.

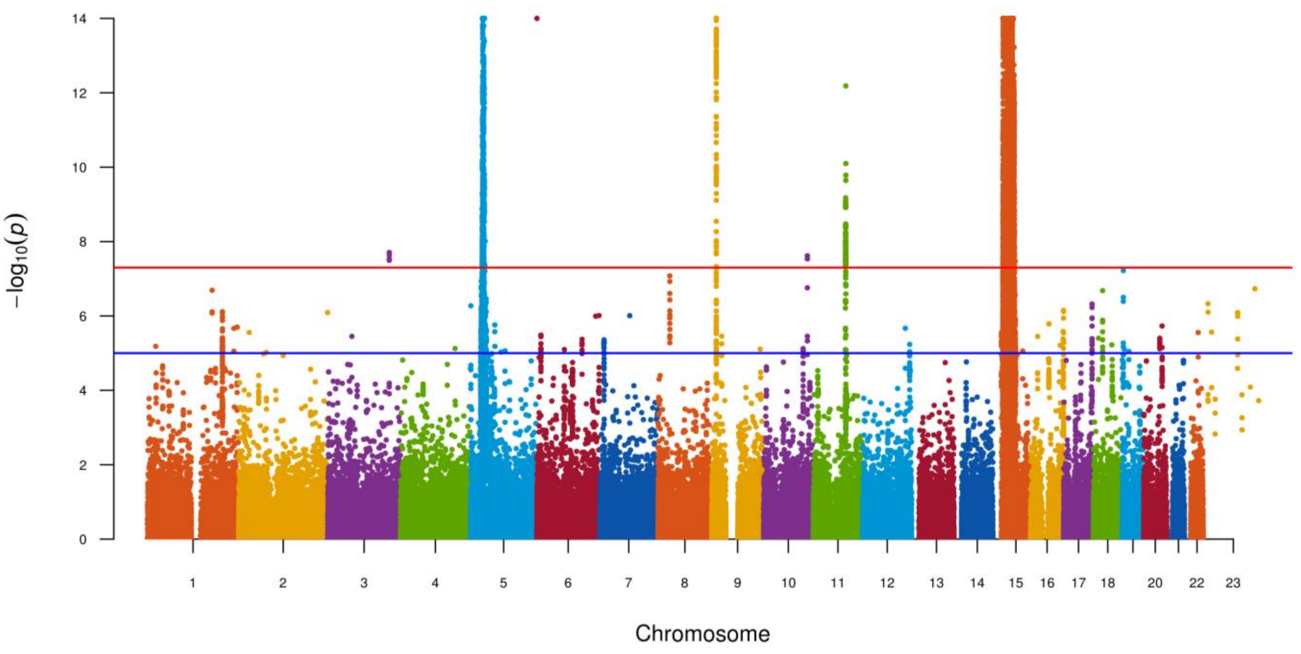

SNPs showing genome-wide significant (P-value = $5x10^{-8}$) are reported below with the index SNPs from Table 1 highlighted in bold. As expected, index SNPs with effect shared across pigmentation traits were found to be significantly associated, whereas SNPs that only affect a particular pigmentation trait were not, consistent with a reduced power of association under this scenario(18). The frequency column in the table below refers to the allele frequency of the minor allele in the CANDELA cohort.

Chromosome	SNP	Position	Nearest gene	Wald P-value	$-log_{10}$(P-value)	Frequency
3	rs9819158	167480661	SERPINI1	1.96E-08	7.71	0.13
**5**	**rs28777**	**33958959**	**SLC45A2**	**9.05E-127**	**126.04**	**0.50**
**6**	**rs12203592**	**396321**	**IRF4**	**3.90E-43**	**42.41**	**0.07**
**10**	**rs11198112**	**119564143**	**EMX2**	**2.41E-08**	**7.62**	**0.16**
**11**	**rs7118677**	**88511524**	**GRM5**	**8.07E-10**	**9.09**	**0.40**
**11**	**rs1126809**	**89017961**	**TYR**	**6.53E-13**	**12.19**	**0.11**
**15**	**rs1800407**	**28230318**	**OCA2**	**9.71E-13**	**12.01**	**0.05**
**15**	**rs1800404**	**28235773**	**OCA2**	**4.45E-18**	**17.35**	**0.41**
**15**	**rs12913832**	**28365618**	**HERC2**	**<1E-200**	**200.00**	**0.23**
**15**	**rs4778249**	**28380518**	**HERC2**	**6.45E-21**	**20.19**	**0.03**
**15**	**rs1426654**	**48426484**	**SLC24A5**	**4.10E-150**	**149.39**	**0.43**

The novel signal of association on 3q26 detected in the multivariate regression analysis (index SNP rs9819158, P-value = $1.96x10^{-8}$ overlaps the *SERPINI1* gene. Interestingly, this SNP was close to suggestive genome-wide significance for C (Saturation; P-value = $9.4x10^{-6}$) and was nominally significant for cos(H) (Hue; P-value = $2.29x10^{-2}$), skin pigmentation (P-value = $7.34x10^{-4}$) and hair color (P-value = $5.29x10^{-3}$). This gene encodes a member of the serpin superfamily of serine proteinase inhibitors and is primarily secreted by axons in the brain (19). Consistent with this finding, *SERPINI1* is highly expressed in different brain related areas and virtually absent in other human tissues according to the GTEx database. Although *SERPINI1* is not known to play a role in pigmentation, other members of the serpin gene family have been reported to affect pigmentation in various organisms(20). In humans, the pigment epithelium-derived factor is encoded by the *SERPINF1* and *SERPINF2* genes (21, 22).

Supplementary Table 7: Allele frequencies of the derived allele for index SNPs

Allele frequencies for CEU, IBS, CHB, and YRI were retrieved from 1000 Genomes Project Phase 3 data. Allele frequencies for Native Americans are based on a subset of individuals with more than 99% Native American ancestry (ADMIXTURE analysis) from (23). This table is plotted in Figure 4B.

**Region**	**SNP**	**Ancestral/ Derived**	**CEU**	**IBS**	**CHB**	**YRI**	**Native American**	**CANDELA**
1q32	rs3795556	T/C	0.23	0.26	0.41	0.36	0.42	0.33
5p13	rs16891982	C/G	0.98	0.82	0.01	0	0.03	0.49
6p25	rs12203592	C/T	0.16	0.13	0	0	0	0.07
9p23	rs10809826	C/G	0.62	0.56	0.01	0.06	0.03	0.29
10q26	rs11198112	C/T	0.17	0.14	0.14	0.17	0.19	0.16
11q14	rs7118677	G/T	0.67	0.74	0.33	0.21	0.05	0.4
11q14	rs1042602	C/A	0.4	0.39	0	0	0.01	0.25
11q14	rs1126809	G/A	0.25	0.29	0	0	0	0.11
15q13	rs4778219	T/C	0.88	0.8	0.11	0.84	0.64	0.75
15q13	rs1800407	C/T	0.08	0.1	0	0	0	0.05
15q13	rs1800404	C/T	0.82	0.73	0.39	0.07	0.31	0.58
15q13	rs12913832	A/G	0.77	0.32	0	0	0.03	0.23
15q13	rs4778249	T/A	0.98	0.98	0.98	0.28	1	0.97
15q21	rs1426654	G/A	1	1	0.03	0.01	0.02	0.56
16q24	rs885479	G/A	0.08	0.02	0.64	0	0.69	0.34
19p13	rs2240751	A/G	0.01	0	0.4	0	0.3	0.19
20q13	rs17422688	G/A	0.15	0.2	0.01	0	0	0.08
22q12	rs5756492	G/A	0.29	0.35	0.54	0.28	0.26	0.26

Supplementary Table 8: Signals of selection at index SNPs in Eurasian populations.

Three selection statistics (iHS, Tajima's D and PBS) were computed at index SNPs (Table 1) in Europeans (CEU) and East Asians (CHB) from the 1000 Genomes Project. P-values were estimated using an outlier approach, by ranking all the genome-wide scores and dividing by the number of values in the distribution, taking the upper tail for iHS and PBS and the lower tail for Tajima's D selection scores. Note that given how these selection statistics are computed, it is not possible to obtain selection scores for each SNP (see Methods). Selection statistics scores with empirical P-values < 0.01 are in bold.

			CEU			CHB		
**Region**	**SNP**	**BP (hg19)**	**|iHS| (P)**	**Tajima's D (P)**	**PBS (P)**	**|iHS| (P)**	**Tajima's D (P)**	**PBS (P)**
1q32	rs3795556	205112911	0.88 (3.49×10^{-1})	1.54 (9.41×10^{-1})	0.05 (2.66×10^{-1})	0.26 (7.85×10^{-1})	0.26 (6.70×10^{-1})	0.02 (4.75×10^{-1})
5p13	rs16891982	33951693	-	**-2.47 (1.13×10^{-3})**	**3.64 (3.61×10^{-7})**	-	0.51 (7.47×10^{-1})	0.00 (1.00)
6p25	rs12203592	396321	0.37 (6.89×10^{-1})	-0.59 (3.12×10^{-1})	-	-	0.12 (6.26×10^{-1})	-
9p23	rs10809826	12682663	1.54 (1.10×10^{-1})	-1.17 (1.48×10^{-1})	**0.82 (7.26×10^{-4})**	-	-1.76 (6.39×10^{-2})	0.10 (2.12×10^{-1})
10q26	rs11198112	119564143	0.64 (4.90×10^{-1})	0.13 (6.18×10^{-1})	0.00 (1.00)	0.60 (5.21×10^{-1})	0.33 (6.93×10^{-1})	0.00 (6.62×10^{-1})
11q14	rs7118677	88511524	1.08 (2.50×10^{-1})	0.40 (7.08×10^{-1})	0.31 (2.68×10^{-2})	1.11 (2.43×10^{-1})	0.89 (8.39×10^{-1})	0.00 (1.00)
11q14	rs1042602	88911696	1.65 (8.79×10^{-2})	0.25 (6.58×10^{-1})	-	-	0.43 (7.23×10^{-1})	-
11q14	rs1126809	89017961	0.47 (6.10×10^{-1})	-1.19 (1.43×10^{-1})	-	-	-0.77 (2.84×10^{-1})	-
15q13	rs4778219	28213850	-	0.48 (7.33×10^{-1})	0.10 (1.54×10^{-1})	-	-1.29 (1.46×10^{-1})	**1.27 (1.71×10^{-4})**
15q13	rs1800407	28230318	0.27 (7.73×10^{-1})	0.13 (6.18×10^{-1})	0.08 (2.01×10^{-1})	-	1.06 (8.72×10^{-1})	0.00 (1.00)
15q13	rs1800404	28235773	0.75 (4.22×10^{-1})	0.13 (6.18×10^{-1})	**0.71 (1.50×10^{-3})**	**3.45 (3.54×10^{-3})**	1.06 (8.72×10^{-1})	0.00 (1.00)
15q13	rs12913832	28365618	**3.16 (6.18×10^{-3})**	**-2.20 (8.88×10^{-3})**	-	-	-1.95 (4.02×10^{-2})	-
15q13	rs4778249	28380518	-	-2.04 (1.87×10^{-2})	0.02 (4.04×10^{-1})	-	-1.19 (1.71×10^{-1})	0.00 (1.00)
15q21	rs1426654	48426484	-	-1.94 (2.67×10^{-2})	**3.87 (2.41×10^{-7})**	-	-1.40 (1.24×10^{-1})	0.00 (1.00)
16q24	rs885479	89986154	-	-0.03 (5.19×10^{-1})	0.00 (1.00)	0.12 (8.99×10^{-1})	0.24 (6.66×10^{-1})	**0.83 (2.07×10^{-3})**
19p13	rs2240751	3548231	-	-0.78 (2.52×10^{-1})	0.00 (1.00)	0.70 (4.55×10^{-1})	-1.71 (7.13×10^{-2})	0.50 (1.37×10^{-2})
20q13	rs17422688	43739119	0.27 (7.73×10^{-1})	-0.77 (2.57×10^{-1})	0.14 (1.07×10^{-1})	-	-1.70 (7.24×10^{-2})	0.00 (1.00)
22q12	rs5756492	37424991	0.37 (6.92×10^{-1})	0.00 (5.72×10^{-1})	0.00 (1.00)	0.18 (8.50×10^{-1})	0.38 (7.07×10^{-1})	0.13 (1.65×10^{-1})

Abbreviations: BP, Base Position

Supplementary Table 9: Enrichment of signatures of positive selection at genomic regions associated with pigmentation traits.

The maximum PBS and iHS selection scores at haplotype blocks showing suggestive association with pigmentation traits (i.e. those containing SNPs with P-values < 10^{-5}) was compared to the distribution of maximum scores at all other haplotype blocks across rest of the genome in East Asians (CHB) and Europeans (CEU) from the 1000 Genomes Project (see Methods). **A)** P-values reported for a one-sided Mann-Whitney *U*-test using PBS selection scores and **B)** iHS selection scores. We did not perform the enrichment analysis using Tajima's D selection scores, as the number of windows with scores was significantly lower compared to that of PBS and iHS, and because the windows would sometimes overlap two different haplotype blocks.

A)

Phenotype	CHB	CEU
Skin color (MI)	$1.67x10^{-18}$	$4.58x10^{-101}$
Hair color (categorical)	$1.60x10^{-4}$	$1.66x10^{-22}$
Eye color (categorical)	$3.75x10^{-15}$	$7.91x10^{-47}$
L (Brightness)	$1.74x10^{-15}$	$7.62x10^{-76}$
cos(H) (Hue)	$5.63x10^{-13}$	$4.77x10^{-5}$
C (Saturation)	$4.25x10^{-18}$	$3.01x10^{-73}$

B)

Phenotype	CHB	CEU
Skin color (MI)	$3.82x10^{-2}$	$1.08x10^{-8}$
Hair color (categorical)	$1.47x10^{-2}$	$5.38x10^{-5}$
Eye color (categorical)	$9.16x10^{-3}$	$6.78x10^{-5}$
L (Brightness)	$1.02x10^{-3}$	$3.32x10^{-6}$
cos(H) (Hue)	$5.84x10^{-4}$	$2.20x10^{-7}$
C (Saturation)	$8.61x10^{-4}$	$6.14x10^{-8}$

Supplementary Table 10: Worldwide populations included in the correlation analysis between the derived allele frequency at pigmentation loci and solar radiation.

Sample size, geographic coordinates (in degrees) and mean annual solar radiation (in kWh/m^2/day) per population ordered by major geographical regions. For our analysis of the Western Eurasian dataset we included all European, North African and Middle Eastern populations. Similarly, for our analysis of the Eastern Eurasian dataset we included all East Asian, South Asian, Southern East Asian, Oceanian and Siberian populations.

**Population**	**Country of origin**	**Major geographical region**	**Sample size**	**Longitude**	**Latitude**	**Solar radiation**	**Reference**
ESN	Nigeria	Africa	95	5.61	6.33	4.81	(24)
GuiGhanaKgal	Botswana	Africa	14	26.00	-22.00	5.85	(25)
GWD	Gambia	Africa	111	-16.32	13.24	5.56	(24)
Juhoansi	Namibia	Africa	15	18.00	-18.00	6.03	(25)
Khwe	Namibia	Africa	14	19.00	-13.00	5.58	(25)
LWK	Kenya	Africa	73	34.77	0.62	5.91	(24)
MSL	Sierra Leone	Africa	69	-10.69	8.21	5.14	(24)
Bantu	South Africa	Africa	19	27.00	-22.00	5.8	(25)
Xun	Angola	Africa	19	15.00	-13.00	5.65	(25)
YRI	Nigeria	Africa	101	3.94	7.38	4.89	(24)
Aymara	Bolivia	Americas	13	-68.20	-16.50	5.3	(23)
Colla	Argentina	Americas	11	-66.32	-24.23	6.2	(26)
Embera	Colombia	Americas	14	-76.00	7.00	4.31	(23)
Mixe	Mexico	Americas	16	-96.58	16.95	5.26	(23)
Nahua	Mexico	Americas	17	-99.08	17.63	6.05	(23)
Quechua	Peru/Bolivia	Americas	11	-72.00	-13.50	5.34	(23)
Wichi	Argentina	Americas	15	-64.10	-23.22	4.6	(26)
CHB	China	East Asia	103	116.40	39.91	4.4	(24)
CHS-FuJian	China	East Asia	25	118.12	25.49	3.63	(24)
CHS-HuNan	China	East Asia	59	111.65	27.54	3.29	(24)
CHS	China	East Asia	13	114.00	32.30	3.83	(24)
JPT	Japan	East Asia	104	138.09	35.81	3.63	(24)
Papuan	Papua New	Oceania	15	143.00	-4.00	4.91	(27)

BEB	Bangladesh	South Asia	83	90.41	23.81	4.65	(24)
GIH	India	South Asia	96	71.00	23.16	5.16	(24)
ITU	India	South Asia	98	79.14	17.88	5.17	(24)
PJL	Pakistan	South Asia	86	74.35	31.56	5.33	(24)
STU	Sri Lanka	South Asia	95	80.77	7.59	5.34	(24)
Bajo	Indonesia	South East Asia	31	122.52	-4.00	5.5	(28)
Burmese	Myanmar	South East Asia	20	96.60	21.63	4.78	(28)
CDX	China	South East Asia	82	100.82	22.02	4.61	(24)
Dusun	Brunei	South East Asia	20	114.68	4.64	5.65	(28)
Igorot	Philippines	South East Asia	21	121.27	16.57	4.67	(28)
KHV	Vietnam	South East Asia	98	106.65	10.81	5.14	(24)
Lebbo	Indonesia	South East Asia	15	117.28	1.45	4.8	(28)
Malay	Singapore	South East Asia	25	103.86	1.35	4.49	(28)
Murut	Brunei	South East Asia	17	115.17	4.61	5.65	(28)
Vietnamese	Vietnam	South East Asia	18	108.41	14.48	4.56	(28)
Evenki	Russia	Siberia	16	92.88	56.01	3.06	(29)
Even	Russia	Siberia	14	151.29	59.58	2.82	(29)
Koryak	Russia	Siberia	15	159.23	62.03	2.61	(29)
Eskimo	Russia	Siberia	10	-173.05	64.68	2.34	(29)
CEU[a]	USA	Europe	91	6.00	52.00	2.69	(24)
FIN	Finland	Europe	99	24.94	60.17	2.73	(24)
France	France	Europe	10	2.00	46.00	3.34	(23)
Cornwall	UK	Europe	29	-4.78	50.47	3.11	(24)
Kent	UK	Europe	31	0.84	51.22	2.81	(24)
Orkney	UK	Europe	21	-3.28	58.79	2.53	(24)
Germany	Germany	Europe	10	10.64	51.11	2.71	(23)
Catilla y Leon	Spain	Europe	12	-4.73	41.65	4.06	(24)
Catalunya	Spain	Europe	10	2.93	41.95	4.11	(24)
Valencia	Spain	Europe	14	-0.50	39.47	4.98	(24)
Portugal (Central)	Spain	Europe	11	-8.19	39.57	4.32	(23)
Portugal (North)	Spain	Europe	13	-8.59	41.15	4.35	(23)
Portugal (South)	Spain	Europe	12	-7.92	37.02	4.88	(23)
Spain (Andalucia)	Spain	Europe	15	-4.81	37.54	4.78	(23)

Spain (Basque)	Spain	Europe	14	0.00	43.00	3.71	(23)
Spanish(Canary Island)	Spain	Europe	14	-16.31	28.49	5.4	(23)
Spanish	Spain	Europe	15	-3.69	40.40	4.4	(23)
TSI	Italy	Europe	106	11.06	43.50	3.91	(24)
Jordan	Jordania	Middle East	15	35.94	31.95	5.17	(23)
Libya	Libya	North Africa	15	17.55	27.00	5.89	(23)
Morocco	Morocco	North Africa	14	-8.01	31.63	5.24	(23)
Tunisia	Tunisia	North Africa	14	10.18	36.81	4.95	(23)

[a] CEU population of Northern and Western European ancestry was assigned the coordinates of Rheden in the Netherlands.

Supplementary Table 11: Correlation between allele frequency at index SNPs and solar radiation.

Allele frequency correlations between the derived allele frequency of the top index SNPs and solar radiation was tested using Bayenv2.0 (see Methods). For each SNP we estimated a Bayes Factor (BF) and Spearman's rank correlation coefficient (rho). SNPs not present in the extended worldwide populations dataset (Supplementary Table 10) or that were fixed in a geographical region were not included in the analysis. Significant SNPs for both the BF and rho's are in bold.

		Worldwide		Africa		Eastern Eurasia		Western Eurasia	
**Region**	**SNP**	**$\log_{10}$(BF) (P)**	**rho (P)**	**$\log_{10}$(BF) (P)**	**rho (P)**	**$\log_{10}$(BF) (P)**	**rho (P)**	**$\log_{10}$(BF) (P)**	**rho (P)**
1q32	rs3795556	-0.80 (0.57)	0.04 (0.634)	-0.66 (0.406)	0.13 (0.547)	-0.54 (0.466)	0.04 (0.77)	-0.96 (0.938)	0.03 (0.841)
5p13	rs16891982	-	-	-	-	-	-	-	-
6p25	rs12203592	-0.87 (0.807)	-0.02 (0.834)	-	-	-0.71 (0.826)	0.04 (0.757)	-0.30 (0.085)	-0.26 (0.025)
9p23	rs10809826	-	-	-	-	-	-	-	-
10q26	rs11198112	-0.80 (0.566)	0.04 (0.642)	-0.55 (0.264)	0.20 (0.344)	-0.66 (0.703)	0.10 (0.496)	-0.69 (0.295)	-0.11 (0.339)
11q14	rs7118677	-	-	-	-	-	-	-	-
11q14	rs1042602	-0.80 (0.592)	-0.05 (0.527)	-0.83 (0.829)	-0.05 (0.81)	-0.54 (0.473)	-0.03 (0.798)	-0.76 (0.368)	-0.07 (0.568)
11q14	rs1126809	-	-	-	-	-	-	-	-
15q13	rs4778219	-	-	-	-	-	-	-	-
15q13	rs1800407	-0.49 (0.194)	0.09 (0.279)	-	-	-0.78 (0.968)	0.06 (0.663)	-0.90 (0.672)	-0.03 (0.818)
15q13	rs1800404	**3.12 (0.002)**	**-0.22 (0.019)**	-0.55 (0.275)	0.01 (0.962)	**1.48 (0.017)**	**-0.30 (0.029)**	-0.78 (0.406)	0.00 (0.985)
15q13	rs12913832	**12.66 (0.001)**	**-0.39 (0.001)**	-0.78 (0.666)	0.16 (0.452)	-0.40 (0.324)	-0.10 (0.461)	**9.40 (0.001)**	**-0.36 (0.003)**
15q13	rs4778249	-	-	-	-	-	-	-	-
15q21	rs1426654	-	-	-	-	-	-	-	-
16q24	rs885479	0.29 (0.029)	-0.14 (0.111)	-	-	**1.97 (0.009)**	**-0.29 (0.033)**	0.25 (0.017)	-0.19 (0.113)
19p13	rs2240751	0.49 (0.016)	-0.14 (0.116)	-	-	**2.32 (0.004)**	**-0.28 (0.047)**	-0.53 (0.172)	-0.05 (0.692)
20q13	rs17422688	-0.95 (0.983)	-0.02 (0.753)	-0.09 (0.076)	0.44 (0.02)	-0.71 (0.827)	-0.09 (0.535)	-0.88 (0.614)	-0.09 (0.428)
22q12	rs5756492	-0.74 (0.447)	-0.06 (0.475)	-0.50 (0.225)	-0.26 (0.224)	0.22 (0.094)	-0.08 (0.561)	-0.91 (0.713)	0.05 (0.694)

Supplementary Table 12: List of reported GWAS associations with pigmentation.

The table below lists 161 SNPs collated from published association studies on pigmentation. Some of these SNPs were not present in the Candela dataset (e.g. SNPs that are not biallelic were excluded from the Candela dataset during QC). A final set of 139 SNPs that were used to do the preliminary analysis to search for 'most important' SNPs are denoted with 'Y' in the 'Used' column. Position is in GrCh37. A1 and A2 are reference and alternative allele(s), respectively.

						A2 allele frequency							
**Chr**	**Position**	**SNP**	**A1**	**A2**	**Gene**	**IBS**	**YRI**	**CHB**	**CLM**	**MXL**	**PEL**	**Used**	**Reference**
1	17722363	rs7538876	A	G	PADI6	0.3178	0.3611	0.233	0.3936	0.2891	0.3941	Y	(30)
1	22124820	rs6659601	G	A	LDLRAD2	0.8458	0.6019	0.7039	0.8032	0.9297	0.9412	Y	(31)
1	67627828	rs117633859	G	A	IL23R	0.03271	0	0.1553	0.04787	0.01562	0.1471	Y	(32)
1	188000243	rs77443641	A	C	PLA2G4A	0	0.09259	0	0	0	0		(31)
1	196942660	rs115105970	T	C	CFHR5	0	0.1019	0	0	0.007812	0.01765		(31)
1	197452914	rs114143322	A	G	CRB1	0	0.01852	0	0.005319	0	0		(33)
1	204344757	rs112725747	T	C	LOC127841	0.004673	0.03704	0	0	0	0		(31)
1	213126565	rs3002288	A	G	VASH2	0.3925	0.05093	0.3932	0.2287	0.2656	0.08235	Y	(34)
1	228997835	rs801114	G	T	RHOU	0.4393	0.8704	0.3689	0.516	0.3516	0.3176	Y	(30)
1	235907825	rs3768056	A	G	LYST	0.7664	0.9074	0.8738	0.7872	0.7266	0.6588	Y	(35) (36)
1	236039877	rs9782955	C	T	LYST	0.771	1	0.8981	0.8032	0.7422	0.6647	Y	(36)
1	244364879	rs76327975	G	C	ZNF238	0	0.03704	0	0	0	0	Y	(31)
2	66820715	rs55767876	G	T	MEIS1	0.08411	0.09722	0.02913	0.04255	0.0625	0.02353	Y	(31)
2	150937112	rs10445747	G	A	RND3	0.07009	0	0	0.01596	0.03125	0.01765	Y	(37)
2	151111030	rs62170035	A	C	RND3	0.02336	0.1111	0	0.02128	0.007812	0.005882	Y	(31)
2	168646041	rs13020412	G	A	B3GALT1	0.09813	0.01389	0	0.03191	0.05469	0.02353	Y	(37)
2	234672639	rs6742078	T	G	UGT1A1	0.2804	0.5046	0.1117	0.3404	0.3672	0.4529	Y	(38)
2	240030484	rs3791406	T	C	HDAC4	0.6729	0.3148	0.5146	0.6702	0.7109	0.6765	Y	(37)
3	9886352	rs6788400	G	A	RPUSD3	0.02336	0.3519	0.02913	0.03723	0.03906	0.005882	Y	(31)
3	100590822	rs9848726	T	A	ABI3BP	0.3271	0.2824	0.2621	0.4681	0.3281	0.3588	Y	(37)
3	187398936	rs79592764	T	C	SST	0	0.1389	0	0.01596	0	0.01176	Y	(31)
4	10398635	rs4698048	G	T	ZNF518B	0.5981	0.6528	0.4903	0.6277	0.6562	0.6235	Y	(37)
4	14776694	rs28479566	T	C	LOC441009	0.004673	0.2269	0	0.005319	0.007812	0.01176	Y	(31)
5	314935	rs7736	C	T	PDCD6	0.07009	0.5694	0.01456	0.09574	0.05469	0.04118	Y	(39)
5	6767312	rs12520016	G	T	PAPD7	0.04673	0.1204	0.004854	0.05319	0.01562	0.02353	Y	(34)
5	33948589	rs35395	C	T	SLC45A2	0.8738	0.2037	0.08738	0.6596	0.4219	0.1824	Y	(1)
5	33951693	rs16891982	G	C	SLC45A2	0.8178	0	0.01456	0.6383	0.4141	0.1588	Y	(31, 36, 40-44)
5	33952106	rs185146	T	C	SLC45A2	0.8738	0.213	0.08738	0.6596	0.4219	0.1882	Y	(37)
5	33955673	rs35391	C	T	SLC45A2	0.8925	0.5787	0.3592	0.6755	0.4844	0.2765	Y	(45)
5	33958959	rs28777	A	C	SLC45A2	0.8738	0.1667	0.08738	0.6543	0.4219	0.1824	Y	(46)
5	33963870	rs26722	T	C	SLC45A2	0.07944	0.06019	0.432	0.2447	0.3203	0.5882	Y	(42, 43, 47, 48)
5	33964210	rs183671	G	T	SLC45A2	0.8551	0.1667	0.07767	0.6543	0.4219	0.1824	Y	(36)
5	33967145	rs35412	G	C	SLC45A2	0.8692	0.5556	0.3738	0.6809	0.4922	0.2824	Y	(36)

5	107400505	rs288139	A	G	FBXL17	0.243	0.1065	0.3252	0.1649	0.1094	0.09412	Y	(49)
5	149210848	rs32579	T	C	PPARGC1B	0.285	0.5972	0.3641	0.383	0.5156	0.5765	Y	(45)
5	150691486	rs428668	C	T	SLC36A2	0.3879	0.5602	0.4417	0.5319	0.5625	0.8	Y	(37)
5	178762064	rs340417	A	C	ADAMTS2	0.09813	0.06019	0.1553	0.1489	0.07812	0.1059	Y	(31)
6	396321	rs12203592	T	C	IRF4	0.1262	0	0	0.03723	0.08594	0.02941	Y	(4) (34, 36, 37, 40, 46, 50)
6	466033	rs1540771	T	C	EXOC2	0.5234	0.06944	0.2524	0.5053	0.5391	0.5353	Y	(41, 43, 51)
6	471136	rs12202284	A	C	EXOC2	0.1776	0.1204	0.004854	0.1064	0.04688	0.04118	Y	(52)
6	475489	rs12210050	T	C	EXOC2	0.1028	0.00463	0	0.04255	0.03125	0.04118	Y	(45)
6	542159	rs6918152	G	A	EXOC2	0.6542	0.09259	0.4806	0.5638	0.5547	0.4	Y	(46)
6	19996808	rs9350204	C	A	MBOAT1	0.1589	0	0.3786	0.1543	0.1719	0.2706	Y	(53)
6	31244789	rs2524069	T	A	HLA-C	0.1682	0.06019	0	0.08511	0.0625	0.05294		(53)
6	32428715	rs9268838	A	G	HLA-DRA	0.2757	0.1389	0.3544	0.3404	0.3125	0.5176		(32)
6	32575658	rs3021304	C	G	HLA-DRB1	0.5374	0.5509	0.6019	0.4362	0.5547	0.6	Y	(32)
6	41729406	rs12661968	C	T	FRS3	0.1449	0.08333	0.1408	0.1702	0.1562	0.1588	Y	(37)
6	92184362	rs73753762	G	A	MIR4643	0	0.1111	0	0	0.007812	0		(31)
6	111993747	rs77437330	A	G	FYN	0.05607	0.02778	0.09709	0.05851	0.02344	0	Y	(31)
7	36685144	rs7794780	C	T	AOAH	0.5701	0.662	0.3107	0.4787	0.4453	0.2941	Y	(31)
7	122042978	rs77462788	C	T	CADPS2	0.02336	0	0	0.02128	0.007812	0.01765		(31)
8	8757628	rs96621	C	T	MFHAS1	0.486	0.5139	0.335	0.3883	0.3594	0.3176	Y	(37)
8	15500967	rs12541402	C	T	TUSC3	0.2991	0	0.08738	0.2021	0.2734	0.1529	Y	(31)
8	132353735	rs4596632	A	G	ADCY8	0.6776	0.4213	0.4029	0.6862	0.7344	0.8118	Y	(49)
9	12396731	rs13289810	G	A	TYRP1	0.3458	0.287	0.03883	0.2128	0.1406	0.04118	Y	(54)
9	12672097	rs1408799	C	T	TYRP1	0.6121	0.1991	0.01456	0.3936	0.3594	0.1353	Y	(34, 41, 55)
9	12704725	rs2733832	T	C	TYRP1	0.5421	0.04167	0.01456	0.3085	0.4297	0.3059	Y	(43)
9	16858084	rs10756819	A	G	BNC2	0.6822	0.03704	0.6165	0.6064	0.6094	0.4765	Y	(38)
9	16864521	rs2153271	T	C	BNC2	0.5935	0.1204	0.199	0.4521	0.2812	0.1588	Y	(40)
9	16901067	rs62543565	A	C	BNC2	0.6355	0.5093	0.2864	0.5	0.3359	0.1647	Y	(50)
10	13605982	rs6602665	C	T	PRPF18	0.004673	0.3056	0.009709	0.01064	0.02344	0.01176	Y	(31)
10	13606490	rs6602666	G	A	PRPF18	0.004673	0.3056	0.009709	0.01064	0.03125	0.01176	Y	(31)
10	24164956	rs11812960	G	A	KIAA1217	0.004673	0.05556	0	0.005319	0	0		(33)
10	25207241	rs151165649	A	G	PRTFDC1	0.01402	0	0	0.01596	0	0.005882	Y	(31)
10	47632167	rs111256285	G	A	ANTXRL	0	0.1204	0	0.01064	0.007812	0	Y	(31)
10	64490495	rs442309	T	C	ZNF365	0.5561	0.4398	0.1942	0.5319	0.4297	0.2941	Y	(32)
11	61106892	rs2513329	C	G	DAK	0.986	0.3102	0.9709	0.8989	0.9766	0.9588	Y	(39)
11	61137147	rs7948623	A	T	TMEM138	1	0.7361	1	0.9574	0.9922	0.9882	Y	(56)
11	68846399	rs35264875	T	A	TPCN2	0.1449	0	0	0.1011	0.1016	0.02941	Y	(43, 55)
11	68855363	rs3829241	A	G	TPCN2	0.3879	0.01852	0.1748	0.3085	0.1875	0.07059	Y	(43)
11	68872843	rs72930659	T	C	TPCN2	0.08411	0	0	0.05851	0.0625	0.02353	Y	(41)
11	88557991	rs10831496	G	A	GRM5	0.2664	0.8889	0.7524	0.5426	0.6172	0.7353	Y	(45)
11	88911494	rs13312741	G	T	TYR	0	0	0	0	0	0		(57)
11	88911696	rs1042602	A	C	TYR	0.3925	0	0	0.3245	0.1797	0.1	Y	(42, 43, 51)
11	88976157	rs12295166	C	T	TYR	0.4065	0	0	0.3245	0.1797	0.1	Y	(42)
11	89011046	rs1393350	A	G	TYR	0.285	0	0	0.1011	0.1406	0.06471	Y	(45, 51)
11	89017961	rs1126809	A	G	TYR	0.2944	0	0	0.1064	0.1406	0.06471	Y	(34, 43, 44)
11	95773435	rs10831469	G	A	MAML2	0.3832	0.1944	0.5049	0.4521	0.5469	0.5765	Y	(1)

11	95895552	rs115019323	A	G	MAML2	0	0.009259	0	0	0.007812	0		(31)
11	131350968	rs12421680	A	G	NTM	0.271	0.6481	0.6748	0.5426	0.7344	0.8294	Y	(34)
12	785468	rs10849455	G	T	NINJ2	0.8318	0.4167	0.7087	0.8298	0.8984	0.8647	Y	(31)
12	41778982	rs1902910	G	A	PDZRN4	0.1916	0.1065	0.1165	0.1277	0.07812	0.04118	Y	(31)
12	54159277	rs7969151	A	G	CALCOCO1	0.2804	0.4074	0.06796	0.2074	0.1484	0.2118	Y	(45)
12	89299746	rs642742	T	C	LOC728084	0.1729	0.9444	0.301	0.2713	0.2656	0.2235	Y	(43)
12	89328335	rs12821256	C	T	LOC728084	0.0514	0	0	0.02128	0.01562	0.005882	Y	(34, 41, 43, 51)
13	78381146	rs975739	T	G	SLAIN1	0.6028	0.5231	0.2087	0.617	0.4922	0.4235	Y	(34)
13	95096013	rs1407995	C	T	DCT	0.7757	0.8148	0.233	0.6862	0.5625	0.3353	Y	(42)
13	106743244	rs188019015	T	C	LOC728192	0	0.03241	0	0	0	0		(31)
13	113819785	rs3024737	G	A	PROZ	0	0.05093	0	0.01064	0	0.01176		(31)
13	114460362	rs7326155	C	T	FAM70B	0.004673	0.05093	0	0	0	0.01176		(31)
14	52310104	rs8015138	C	A	GNG2	0.6028	0.1713	0.1117	0.3138	0.2812	0.2235	Y	(34)
14	65783804	rs73278043	G	C	MIR4708	0	0.06944	0.09709	0.02128	0	0.005882		(33)
14	92773663	rs12896399	T	G	SLC24A4	0.3458	0.00463	0.2913	0.2872	0.2266	0.2706	Y	(34, 40, 43, 46, 51)
14	92800004	rs8014907	T	A	SLC24A4	0.2243	0.3796	0.05825	0.1489	0.1719	0.09412	Y	(41)
14	92866905	rs10133804	T	A,C	SLC24A4	0.019	0.148	0	0.005	0.016	0.006	Y	(31)
14	97103807	rs17094273	A	G	PAPOLA	0.1168	0.3056	0.1602	0.1117	0.09375	0.05294	Y	(45)
15	28196821	rs7173419	C	T	OCA2	0.715	0.2778	0.08252	0.5691	0.4688	0.3941	Y	(34)
15	28197037	rs1800414	C	T	OCA2	0	0	0.5922	0	0	0	Y	(57) (58) (35, 59) (43, 52, 60)
15	28228553	rs74653330	T	C	OCA2	0	0	0.01942	0.01596	0	0		(57) (58) (35)
15	28230318	rs1800407	T	C	OCA2	0.09813	0	0.004854	0.04787	0.03125	0	Y	(35) (43, 61)
15	28235773	rs1800404	T	C	OCA2	0.7336	0.06944	0.3932	0.6436	0.5469	0.4647	Y	(56)
15	28335820	rs4778138	G	A	OCA2	0.3037	0.7685	0.7282	0.266	0.3125	0.2		(46) (31)
15	28365618	rs12913832	G	A	HERC2	0.3224	0	0	0.266	0.1797	0.1118	Y	(4) (40, 46) (34, 36, 43, 62)
15	28511997	rs79097182	T	C	HERC2	0.1869	0	0.4126	0.08511	0.08594	0.04706	Y	(41)
15	28514281	rs4932620	C	T	HERC2	0.9813	0.9167	0.9806	0.9734	0.9922	0.9941	Y	(56)
15	28530182	rs1667394	T	C	HERC2	0.5654	0.0463	0.2379	0.5745	0.5469	0.6235	Y	(49) (51)
15	28533565	rs1667392	G	A,C	HERC2	0.355	0	0	0.005	0.172	0.112	Y	(36)
15	29006093	rs8033165	T	C	WHAMMP2	0.2757	0.09259	0	0.25	0.1719	0.08235	Y	(46)
15	29261716	rs4424881	T	C	APBA2	0.1168	0.9444	0.6117	0.234	0.1875	0.3118	Y	(1)
15	48392165	rs1834640	G	A	SLC24A5	0.004673	0.9861	0.9029	0.2181	0.3438	0.5529	Y	(42)
15	48400199	rs2675345	G	A	SLC24A5	0.004673	0.9861	0.767	0.25	0.3828	0.5647	Y	(31)
15	48426484	rs1426654	G	A	SLC24A5	0	0.9861	0.9709	0.2819	0.4844	0.7176	Y	(4) (1) (56) (33, 42) (43)
15	48433494	rs2470102	G	A	MYEF2	0	0.9861	0.767	0.2553	0.375	0.5765	Y	(1) (31, 33)
15	50307416	rs2899446	G	A	ATP8B4	0.3925	0.8519	0.6942	0.4734	0.6016	0.7765	Y	(31)
15	50308950	rs8033655	G	A	ATP8B4	0.3925	0.8565	0.6942	0.4734	0.6016	0.7765	Y	(31)
15	59175467	rs28753701	T	C	SLTM	1	0.8519	1	1	1	1		(31)
15	61817211	rs532282237	T	C	LOC107984782	0	0.005	0	0	0	0		(31)
15	66319806	rs61310892	A	G	MEGF11	0	0.07407	0	0.01064	0	0.005882		(31)
16	26959698	rs62029775	T	C	C16orf82	0.5701	0.375	0.3786	0.6809	0.6875	0.7706	Y	(31)
16	86363054	rs2353688	C	T	LOC732275	0.02804	0.00463	0.03398	0.06915	0.1328	0.2176	Y	(41)
16	89667337	rs154659	C	T	CPNE7	0.2103	0.5556	0.4126	0.3245	0.4219	0.6059	Y	(45)

16	89736157	rs35063026	T	C	C16orf55	0.03271	0	0	0.02128	0.02344	0.01765	Y	(50)
16	89755903	rs258322	G	A	CDK10	0.9439	0.838	0.3932	0.8457	0.8594	0.7118	Y	(46)
16	89818732	rs12931267	G	C	FANCA	0.03271	0	0.02427	0.02128	0.007812	0.005882	Y	(40)
16	89887249	rs35096708	A	G	FANCA	0.1822	0	0.009709	0.1011	0.2188	0.07059	Y	(37)
16	89985844	rs1805005	T	G	MC1R	0.1542	0	0	0.1011	0.03906	0.01176	Y	(36, 43)
16	89985918	rs1805006	A	C	MC1R	0.009346	0	0	0	0.007812	0	Y	(36, 43)
16	89985940	rs2228479	A	G	MC1R	0.0514	0	0.2136	0.01064	0.04688	0.01176	Y	(36, 43)
16	89986091	rs11547464	A	G	MC1R	0.02804	0	0	0.01064	0.007812	0	Y	(43)
16	89986117	rs1805007	T	C	MC1R	0.03271	0	0.004854	0.02128	0.007812	0.005882	Y	(34, 36, 43, 51)
16	89986130	rs1110400	C	T	MC1R	0.02336	0	0	0.005319	0	0.005882	Y	(36, 43)
16	89986144	rs1805008	T	C	MC1R	0.009346	0	0	0.005319	0	0	Y	(36, 43, 51)
16	89986154	rs885479	A	G	MC1R	0.01869	0	0.6408	0.2074	0.3906	0.6471	Y	(43, 63, 64)
16	89986546	rs1805009	C	G	MC1R	0.01402	0	0	0.02128	0	0	Y	(36, 43)
16	90022693	rs146972365	C	T	DEF8	0.03738	0.00463	0	0.02128	0.01562	0.005882	Y	(41)
16	90026512	rs4268748	C	T	DEF8	0.1449	0.412	0.2282	0.133	0.1172	0.05294	Y	(36, 37)
16	90054709	rs8063160	C	T	AFG3L1P	0.05607	0.2407	0	0.06383	0.03125	0.03529	Y	(41)
16	90084561	rs11648785	T	C	DBNDD1	0.4065	0.1065	0.2039	0.4468	0.1875	0.2235	Y	(45)
17	33823098	rs117307642	T	C	SLFN12L	0.06075	0	0	0.04255	0.007812	0.01176	Y	(31)
17	79591813	rs7219915	T	C	NPLOC4	0.4626	0.838	0.9951	0.6915	0.7031	0.8647	Y	(62)
17	79596811	rs9894429	T	C	NPLOC4	0.4159	0.7222	0.8058	0.6649	0.6719	0.8471	Y	(62)
17	79664426	rs12452184	T	C	HGS	0.5374	0.6944	0.3447	0.5213	0.5234	0.4824	Y	(62)
18	11451676	rs74791047	A	T	GNAL	0	0.1065	0	0.005319	0	0	Y	(33)
19	3544892	rs56203814	T	C	C19orf28	0.01402	0.2222	0	0.01064	0.007812	0.005882	Y	(56)
19	3545022	rs10424065	T	C	C19orf28	0.01869	0.2685	0	0.02128	0.007812	0.005882	Y	(56)
19	54364168	rs62143248	T	C	MYADM	0.07477	0.2269	0	0.06383	0.03125	0.01176	Y	(31)
20	32665748	rs6059655	G	A	RALY	0.9766	1	1	0.984	0.9922	1		(36, 50)
20	32704627	rs6142102	G	C	EIF2S2	0.7523	0.3843	0.8689	0.6755	0.7422	0.4353	Y	(31)
20	32729444	rs4911414	G	T	EIF2S2	0.757	0.8704	0.8689	0.7447	0.75	0.4412	Y	(55) (34)
20	32738612	rs1015362	T	C	EIF2S2	0.257	0.8519	0.1311	0.3351	0.2969	0.5647	Y	(55)
20	32856998	rs6058017	G	A	ASIP	0.1121	0.8148	0.2427	0.133	0.07812	0.01765	Y	(42, 65)
20	33355046	rs4911442	A	G	NCOA6	0.9159	1	0.9951	0.9681	0.9766	0.9882	Y	(43, 63)
20	33867697	rs619865	G	A	EIF6	0.9252	1	1	0.9628	0.9844	0.9824	Y	(40)
20	36662831	rs755107	A	C,G	RPRD1B	0.005	0.328	0.184	0.162	0.125	0.059	Y	(31)
21	38491095	rs1003719	G	A	TTC3	0.6075	0.3056	0.3544	0.4149	0.4766	0.3176	Y	(62)
21	38507572	rs2252893	C	T	TTC3	0.4626	0.6991	0.6165	0.6596	0.5547	0.7059	Y	(62)
21	38510616	rs2835621	A	G	TTC3	0.4626	0.6991	0.6165	0.6649	0.5547	0.7059	Y	(62)
21	38521842	rs2835630	G	A	TTC3	0.4766	0.6991	0.6602	0.6649	0.5547	0.7059	Y	(62)
21	38580309	rs7277820	G	A	DSCR9	0.472	0.6991	0.6602	0.6649	0.5547	0.7059	Y	(62)
21	43227915	rs7279297	G	A	PRDM15	0.3411	0.5093	0.5388	0.2926	0.3047	0.1882	Y	(45)
22	31113081	rs201429679	T	G	OSBP2	0.2056	0.4213	0.3835	0.3298	0.3438	0.5059		(31)

Supplementary Table 13: Proportion of trait variation explained by each SNP

Proportion of total trait variation explained by each SNP (in unconditional GWAS) of Table 1 is listed below.

			**Skin**	**Hair**	**Eye**			
**Region**	**Candidate gene**	**SNP**	MI	Categorical	Categorical	L (Brightness)	C (Saturation)	cos(H) (Hue)
1q32	DSTYK	rs3795556	0.02	0.00	0.00	0.10	0.45	0.02
5p13	SLC45A2	rs1689198	6.07	3.78	0.87	0.89	0.40	0.24
6p25	IRF4	rs1220359	0.44	0.71	0.64	0.73	0.17	0.07
9p23	TYRP1	rs1080982	0.13	0.06	0.54	0.85	0.48	0.11
10q26	EMX2	rs1119811	0.48	0.00	0.01	0.01	0.00	0.01
11q14	GRM5	rs7118677	0.45	0.30	0.00	0.00	0.01	0.01
11q14	TYR	rs1042602	0.46	0.30	0.00	0.01	0.07	0.00
11q14	TYR	rs1126809	0.44	0.28	0.20	0.28	0.05	0.19
15q13	OCA2	rs4778219	0.00	0.00	0.05	0.04	0.00	0.03
15q13	OCA2	rs1800407	0.41	0.05	0.09	0.04	0.44	0.35
15q13	OCA2	rs1800404	0.52	0.10	0.61	1.01	0.38	0.07
15q13	HERC2	rs1291383	0.90	5.95	25.64	26.74	0.40	6.35
15q13	HERC2	rs4778249	0.27	0.14	0.56	1.15	0.98	0.01
15q21	SLC24A5	rs1426654	6.57	1.01	1.51	2.80	3.19	0.01
16q24	MC1R	rs885479	0.33	0.05	0.00	0.00	0.00	0.00
19p13	MFSD12	rs2240751	0.53	0.00	0.01	0.00	0.04	0.00
20q13	WFDC5	rs1742268	0.01	0.00	0.00	0.02	0.00	0.52
22q12	MPST	rs5756492	0.10	0.00	0.07	0.09	0.45	0.03

Supplementary Figures

Supplementary Figure 1: Eye landmarking protocol

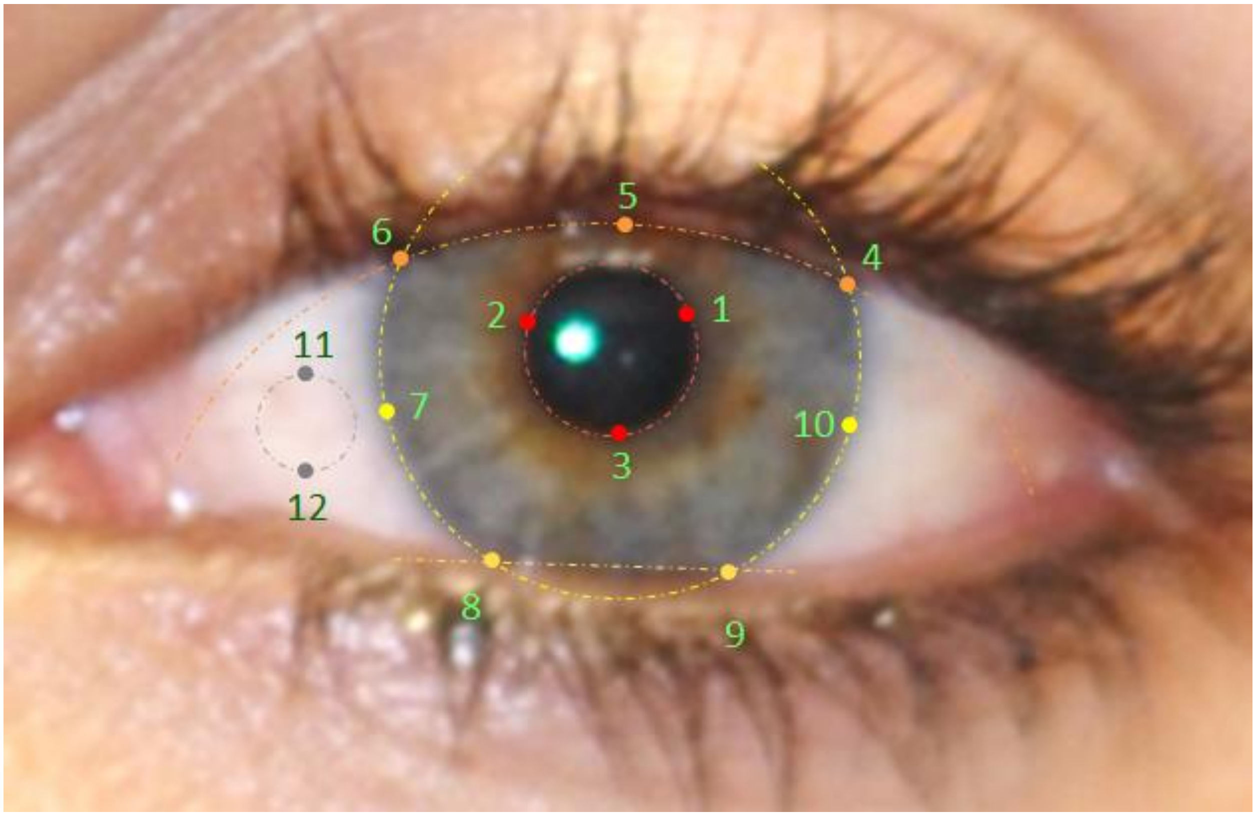

Landmarking software was developed in MatLab R2013b. A rectangular area around one of the eyes was selected by zooming-in using the frontal facial photograph of each research subject. A total of 12 landmarks were then positioned. Landmarks 1-3 were placed on the circular boundary of the pupil (the inner iris boundary). These landmarks were positioned equidistantly from each other in order to improve the accuracy of a circle fit. Landmarks 4 and 6 were placed at the points where the lower boundary of the upper eyelid meets the outer iris boundary (if eyelashes partially cover the iris, the lower boundary of the eyelashes were used instead). Landmark 5 was placed midway between landmarks 4 and 6, at the center of the lower boundary of the upper eyelid (it is usually not on the outer iris boundary). Landmarks 8 and 9 are placed where the upper boundary of the lower eyelid meets the outer iris boundary. In some cases the lower eyelid did not overlap the iris. In such cases, landmarks 8 and 9 were considered identical, being the lowermost point on the outer iris boundary. The same point was clicked twice to indicate to the software that the lower eyelid did not overlap the iris. Because there would be slight variations when trying to click on the exact same point twice, a threshold was decided – If the distance between points 8 & 9 is less than 1/4th of the distance between points 1 & 2, then they are considered to be the same point and the lower eyelid boundary is ignored. Point 9 is then taken to be identical to point 8 and is ignored. Landmark 7 was placed on the outer iris

boundary (where the iris meets the sclera), halfway between landmarks 6 and 8. Similarly, landmark 10 was placed halfway between landmarks 9 and 4. Landmarks 11 and 12 were considered to be the opposite edges of the diameter of a circle placed on the whitest region of the sclera (i.e. an area without major reflections, blood vessels etc).

Landmarks 1-10 are on the visible iris boundaries, outer (relative to the sclera) or inner (relative to the pupil). Photographs usually do not have a very sharp edge around the iris boundaries but rather a gradient of color to black/dark (pupil) or white (sclera). Landmarking was done conservatively, i.e. landmarks were placed on the edges of the iris before the gradient started. This was done so that the lightening or darkening of colors near the edges does not affect the estimated average color of the iris.

Supplementary Figure 2: Digital eye color extraction

Exclusion masks:

Eye landmarks were used to define four exclusion areas (corresponding to pupil, sclera, upper and lower eyelids). Landmarks 1-3 are used to fit a circle around the pupil denoting the pupil exclusion mask (11B). Landmarks 4 and 6-10 are used to fit a circle along the outer iris boundary. All points outside the circle are excluded (i. e. sclera exclusion mask, Suppl. Figure 2C). Landmarks 4-6 are used to fit a circle that approximates the upper eyelid boundary in that region. Points above (i.e. outside) the circle are excluded, as shown in Suppl. Figure 2D. As the lower eyelid usually covers a much smaller area of the iris compared to the upper eyelid, a simpler masking is enough. Landmarks 8 and 9 are used to fit a straight line, and all points below the line are excluded (Suppl. Figure 2E). As described earlier, there are instances when the lower eyelid does not cover the iris, in which case this exclusion criteria is ignored. To avoid including the gradient areas at the border of these regions, the pupil exclusion mask was extended outward by a (heuristic) threshold of 10%, while the other three masks were shrunk inwards by a threshold of 10%.

Color adjustment:

The pupil is used to define black. However, pixels in the pupil circle can show variation in color. Thus the 'black' color of the pupil differs from the actual RGB (0,0,0) black value. To set the black point of the image we used the lower 10% quantile for each of the Red, Green and Blue channels of all pixels in the pupil. The circle defined by landmarks 11-12 in the sclera was used to define a white point. To set the white point we took the upper 80% quantile for each of the Red, Green and Blue channels. Using these black and white points for each channel, a linear transformation is used to adjust the colors for each channel separately, so that the black and white points have values of 0 and 1 respectively:

$$y = \frac{(x - b)}{(w - b)} \quad (1)$$

where x is the old pixel value for a particular channel, and b is the black point and w the white point for the channel. y is the new pixel value. This linear transformation is applied to all pixels in the image for each channel separately. Adjusting the channels separately helps correct color casts, e.g. when the background light has a tint instead of being white. This also standardizes varying illumination levels across pictures.

Highlight removal:

The detectability of highlights, i.e. reflections from the camera flash, depends on the color of the eye, because light colored eyes have various visible structures in the stroma that are much lighter colored than the average color of the iris, and therefore may be falsely detected as highlights. By looking at the histogram of colors in the blue channel for various eye colors, a heuristic threshold of 30% is

determined to classify an eye into light vs. dark – if the median value of pixels in the blue channel in an eye is >0.3, it is considered light. In such a case, the threshold of considering pixels as highlights is 67%, i.e. if the brightness of any pixel (determined by its grey level, after converting the RGB values into greyscale) is >0.67 it is considered as a highlight and excluded. For dark eyes, the exclusion threshold is 55%. These thresholds were also constructed heuristically.

Final exclusion mask:

The final exclusion area is calculated combining exclusion areas of pupil, sclera, upper and lower eyelids, and highlights. Any pixel that is excluded in any one of these masks are excluded from the final iris area. The final inclusion area is shown in white in Suppl. Figure 2F. This inclusion-exclusion mask is then superimposed on the original eye picture, as shown is Suppl. Figure 2G, where all excluded areas are marked green.

Figure 2A: Landmarks & boundaries drawn on an eye.

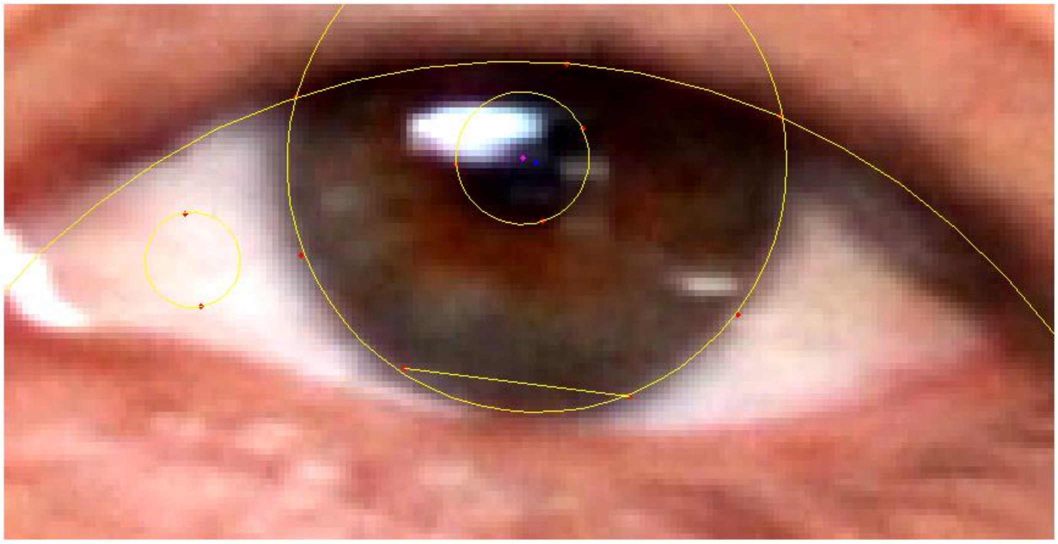

Figure 2B: Exclusion area for pupil, using landmarks 1-3. Black denotes excluded pixels.

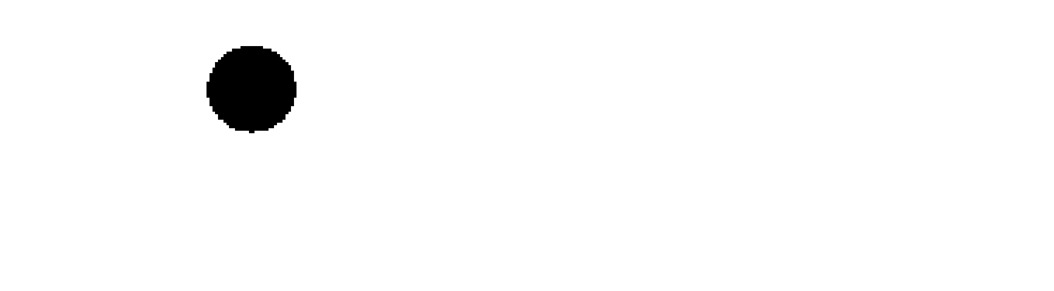

Figure 2C: Exclusion area for sclera, using landmarks 4 and 6-10.

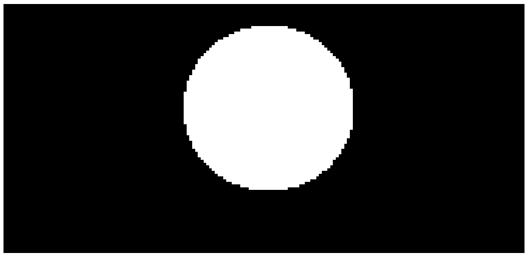

Figure 2D: Exclusion area for upper eyelid, drawn using landmarks 4-6.

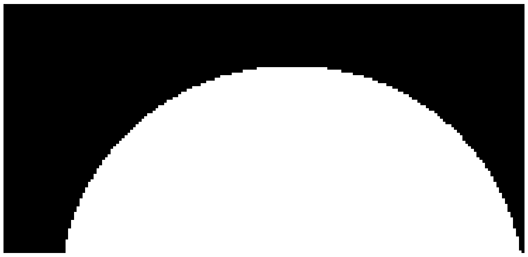

Figure 2E: Exclusion area for lower eyelid, drawn using landmarks 8-9.

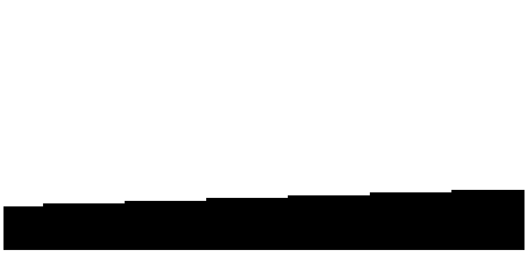

Figure 2F: Final exclusion area combining pupil, sclera, upper and lower eyelids, and highlights. The white region corresponds to iris and it is retained.

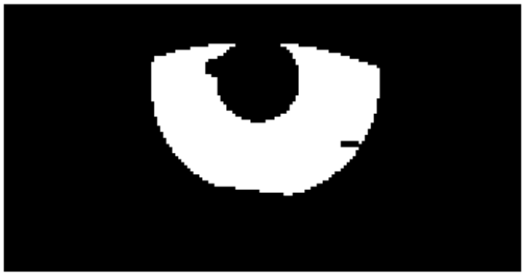

Figure 2G: Exclusion mask superimposed on the picture of the eye. Areas tinted green are excluded.

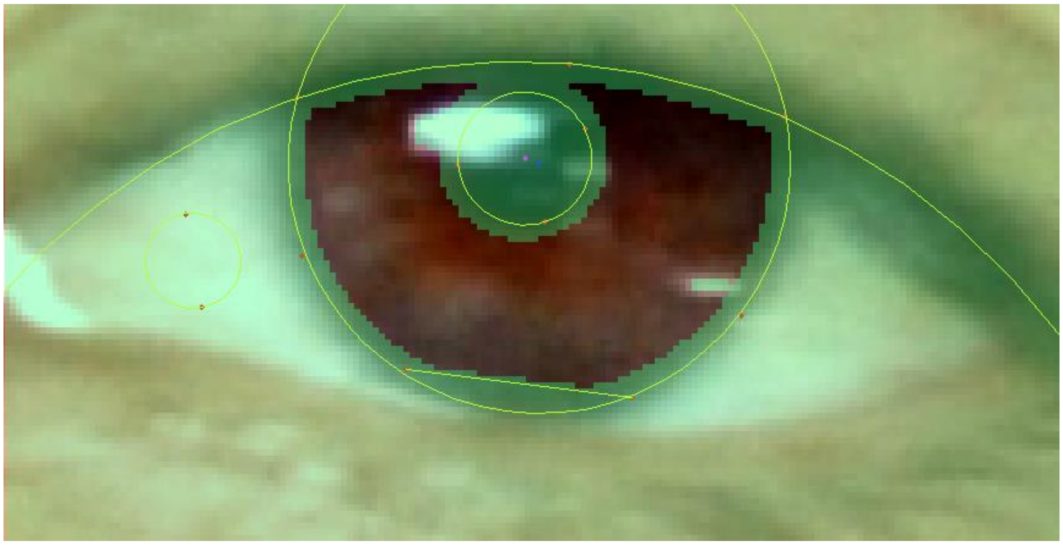

Average iris color:

To reduce the influence of any remaining artifacts, and to get a more robust estimate, the average color was obtained as the multivariate median of the R, G, B values for pixels in the iris.

Supplementary Figure 3: Color spaces used in the eye pigmentation analysis

Various color spaces were considered in the genetic analyses. Their relative advantages and disadvantages were considered before choosing the best color space for the GWAS study.

RGB Space:

RGB is the default color space used in electronic image representation, such as the digital photographs obtained from the CANDELA volunteers. This might not represent the best color space for our analyses as the choice of color space depends partly on the colors being represented. In our study sample eye colors primarily show various shades of brown, with only few blue or grey colors and we do not observe actual green, nor many other RGB colors (such as proper yellow, orange, violet, purple; see Figure 2d). It can be seen that the 3D RGB data cloud lies in fact on a 2-dimensional subspace (Suppl. Figure 3 A and B). Because of this our R, G, B values are highly correlated (Supplementary Table 2C). An increase in each RGB channel correlates with an increase in overall brightness, which is a major axis of variation in eye colors as it is inversely proportional to the concentration of melanin present in the iris.

Figure 3 A and B: Each dot denotes the average color of one iris in RGB color space (cube).

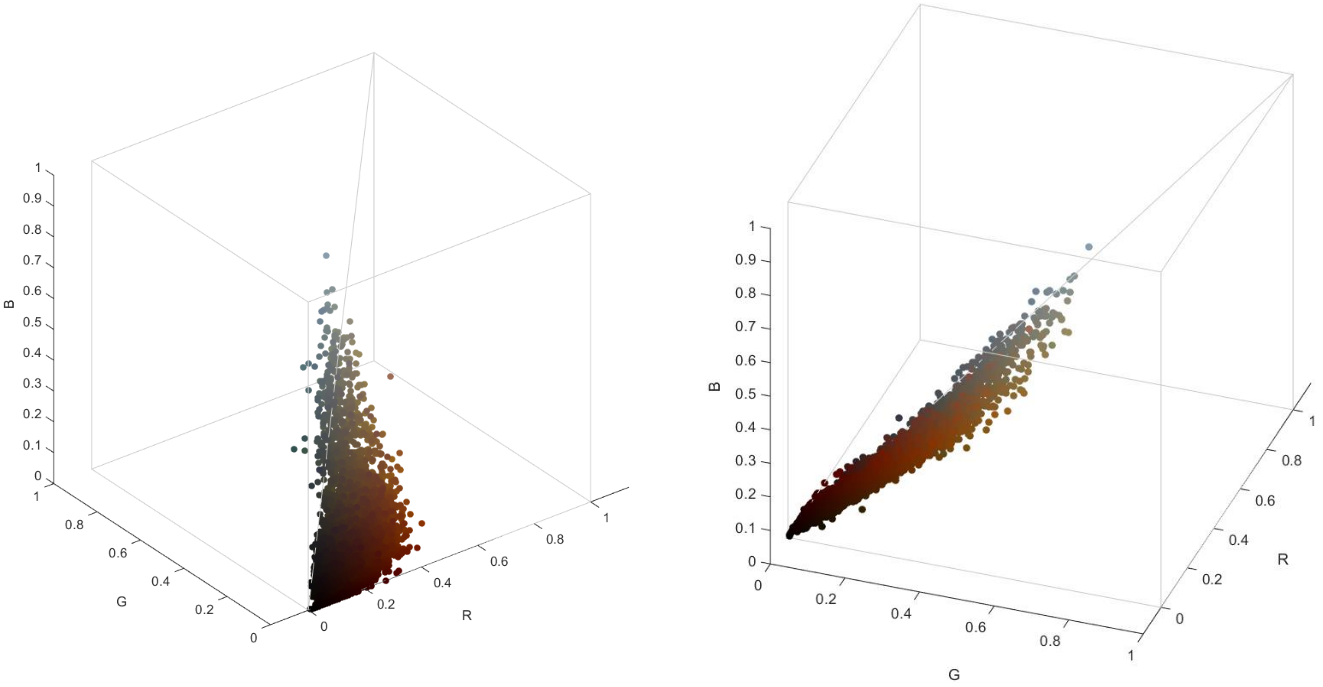

RGB Principal Components space:

Figure 3 C: Biplot of RGB Principal components 1 and 2. PC1 & PC2 are along X & Y axes respectively, while the original R, G, B axes are projected onto the PC1-PC2 space and shown in grey.

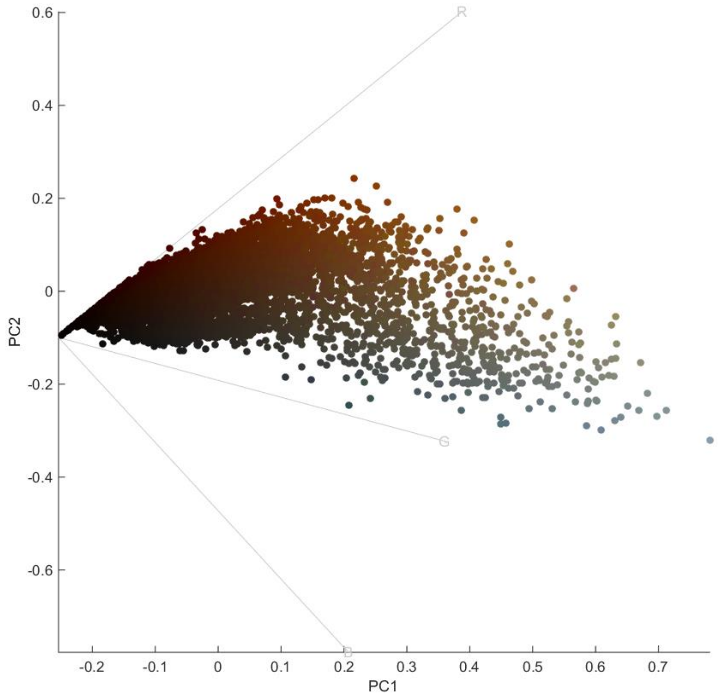

As shown above, the iris color RGB data cloud lies nearly on a 2-dimensional subspace. This is also reflected in the three RGB principal components having variances of: 6.04, 1.12 and 0.04 respectively. Thus, the first two RGB PCs provide a nearly complete summary of the color data (explaining 99.5% of the total variance). PC1 captures mainly the dark-to-light variation while PC2 is mostly blue-to-brown variation. The RGB values are all highly correlated with PC1. One can see in the biplot above that all the RGB values are oriented towards the axis of PC1. Using the RGB Principal components in the genetic analyses has the advantage that PCs are uncorrelated, in contrast to the original RGB values which are highly correlated.

This principal components method is commonly used in analysis of skin pigmentation and detection of pigmentation spots (66).

HSV color space:

The HSV (HSB) color space is a perceptual color space that attempts to separate different aspects of color into different dimensions: Hue, Saturation, and Value (also known as Brightness). It is attractive because these three basic aspects of color are perceived differently, thus separating them into independent axes are useful. E.g. light brown can be distinguished from dark brown simply by change of brightness (V) while Hue (color tone) and Saturation remain the same.

A variation of the HSV model is the HSL model where the measure of brightness, Value (V), is replaced by Lightness (L). The main difference between Value and Lightness is that Value goes from black (V=0) to full color (V=1), while Lightness goes from black (L=0) to full color (L=0.5) to white (L=1). Value only leads to white at V=1 in absence of color, i.e. when S=0.

The calculation of Saturation in the HSL model is slightly different to that in the HSV model.

The definitions of these variables according to CIE (International Commission on Illumination, http://eilv.cie.co.at) are:

Hue: "attribute of a visual perception according to which an area appears to be similar to one of the colours: red, yellow, green, and blue, or to a combination of adjacent pairs of these colours considered in a closed ring".

Value (Brightness): "Attribute of a visual perception according to which an area appears to emit, or reflect, more or less light."

Lightness: "brightness of an area judged relative to the brightness of a similarly illuminated area that appears to be white or highly transmitting".

Saturation: "colourfulness of an area judged in proportion to its brightness".

The HSV model or a subset of the variables have been used in eye and skin pigmentation studies (36, 62).

Hue is depicted as a color circle going from 0° to 360°, with the three primary (RGB) colors set at three equidistant points: R at 0°, G at 120° and B at 240°. The intermediate colors yellow, cyan and magenta fall in between.

Due to the circular nature of Hue, the HSV or HSL colorspaces are cylindrical in shape. Being a circular variable, Hue cannot be used directly in quantitative analysis such as regressions which require a continuous variable. It has a discrete jump at 360°, e.g. 355° + 10° = 5°, which violates the additive nature of continuous variables. For such purposes Hue is commonly converted into trigonometric functions before using: cos(H) and sin(H). These trigonometric functions are periodic and therefore avoid the jump at 360°, e.g. cos(360°) = cos(0°).

Figure 3 D, E, F: set of all iris color values in the cylindrical HSV color space.

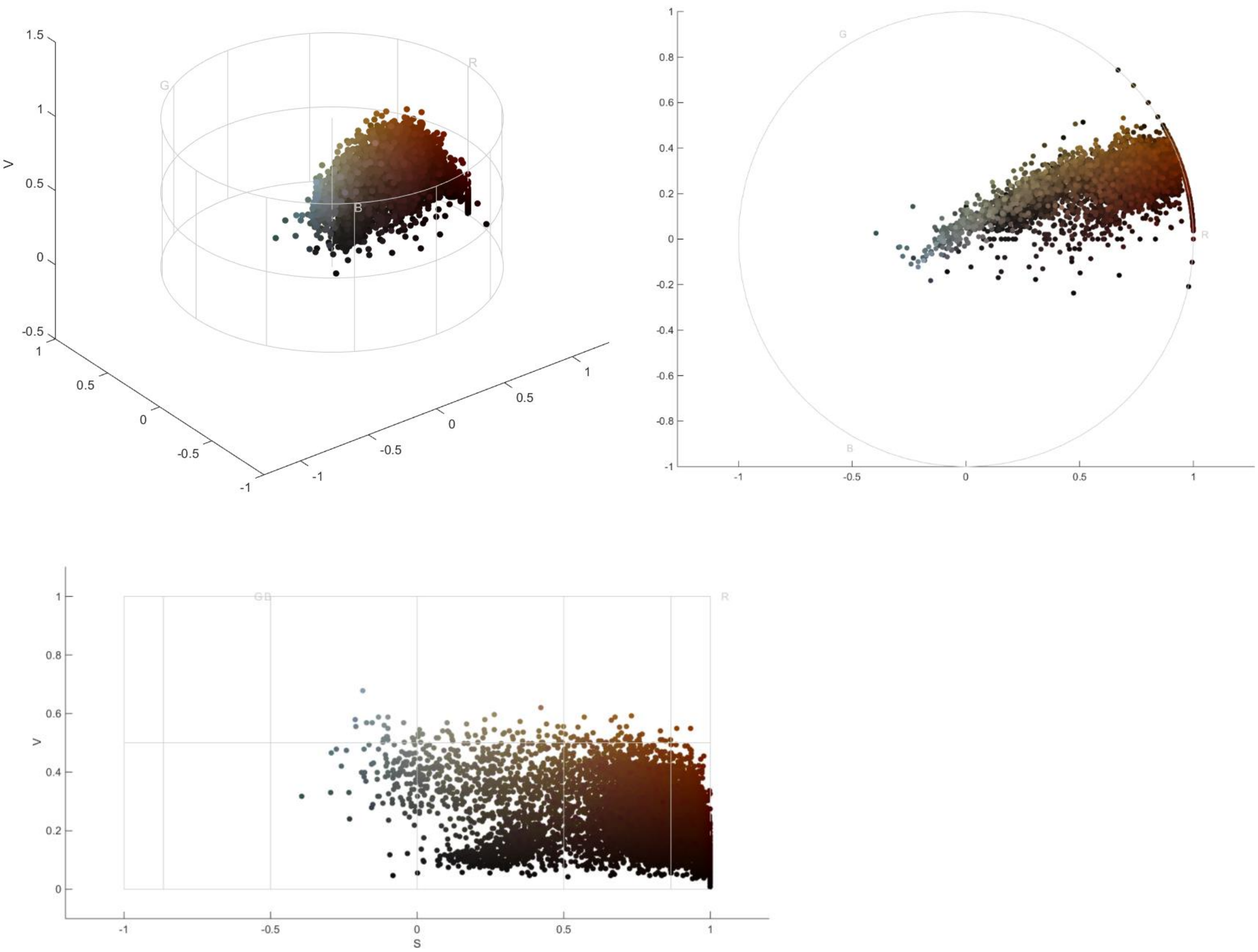

HCL color space:

Figure 3 G, H, I: set of all iris color values in the bicone HCL color space.

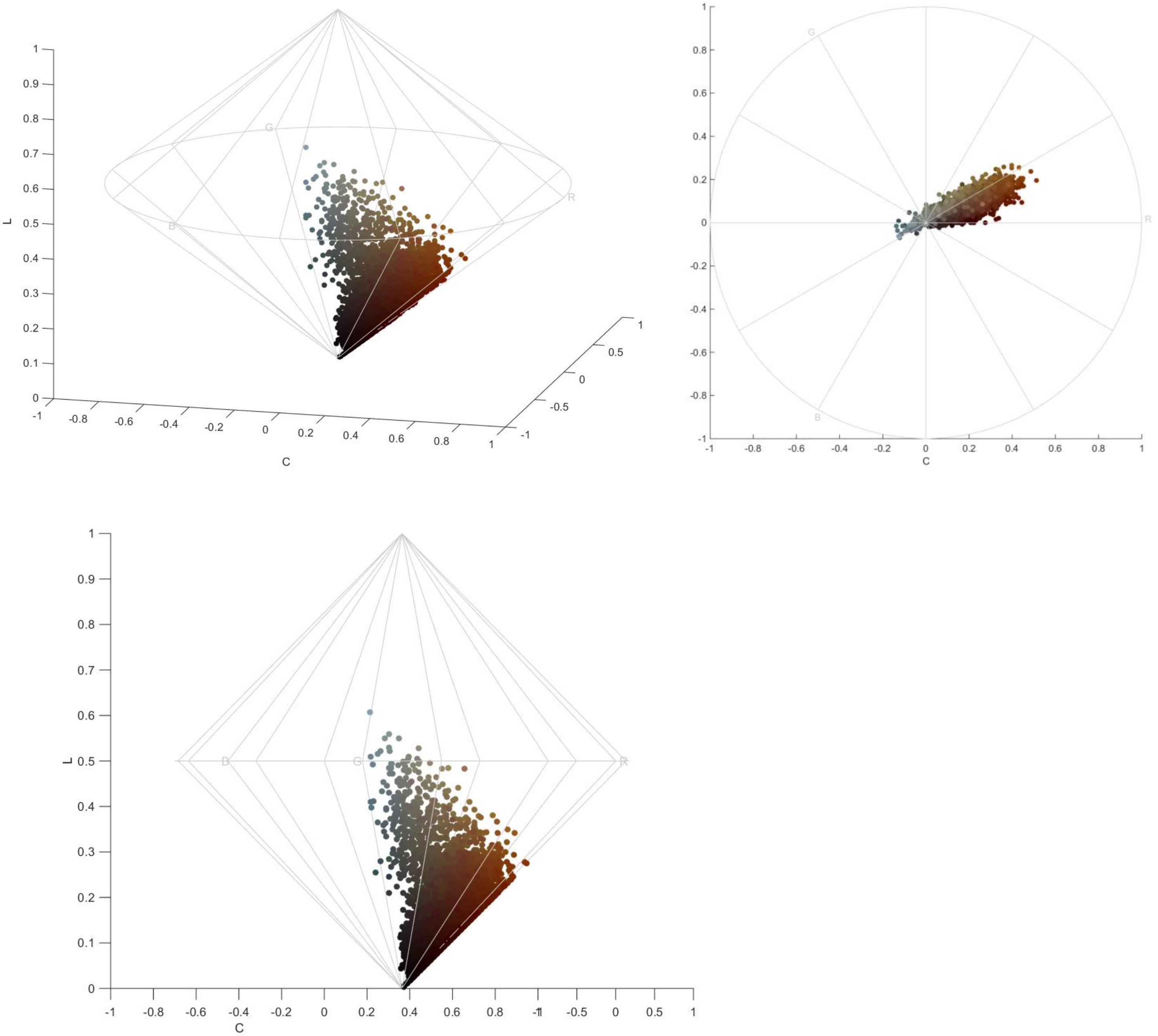

A problem with the Saturation variable in HSV or HSL color models is that its effect on color is rather heavily dependent on brightness, as seen from its definition. For example, when V=0 (or L=0) representing complete black, changing the value of S doesn't have any effect on the color at all. To address this, a new variable Chroma (C) is commonly used in conjunction with Lightness, which restricts the range of values of C to avoid such kind of non-uniqueness in color.

$C \leq L$ if $0 \leq L \leq 0.5$, and $C \leq 1\text{-}L$ if $0.5 \leq L \leq 1$.

Which means C has only an allowable value of 0 if L=0 or L=1, at black or white color.

This restriction on the value of C leads to a bicone colorspace for HCL color model.

Chroma is defined according to CIE as:

"colourfulness of an area judged as a proportion of the brightness of a similarly illuminated area that appears white or highly transmitting".

As seen in the HCL plots above, the data points approximately lie on a vertical 2-d plane that passes through the central line S=0. The axis of this plane is estimated to be the median Hue, which is $H=20^\circ$ in this case. Due to the circular nature of Hue as a variable, mean is not a robust measure of average in this case. Thus, prior to applying the trigonometric functions, the Hue variable was standardized by subtracting the median = 20° from its values. $\cos(H-20^\circ)$, from now on referred to as $\cos(H)$, is close to 1 or -1 most of the times, while $\sin(H-20^\circ)$ is usually small.

The 2-d plane on which the data points approximately lie is thus characterized by two axes: L and $C\times\cos(H)$. Because the axes cos(H) and sin(H) are perpendicular, a projection of all data points onto this plane can simply be attained by ignoring the sin(H) component.

The HSV, HSL and HCL color models however have one criticism, that it doesn't represent perception very accurately. For example, fully saturated red and fully saturated yellow are given the same brightness (L or V) values, though in our perception yellow is a much brighter color than red. However, this is not a problem in our case as our data range is narrower and very few tones of color are present in the data.

Lab color space:

Figure 3 J, K: set of all iris colors in Lab color space (color cube).

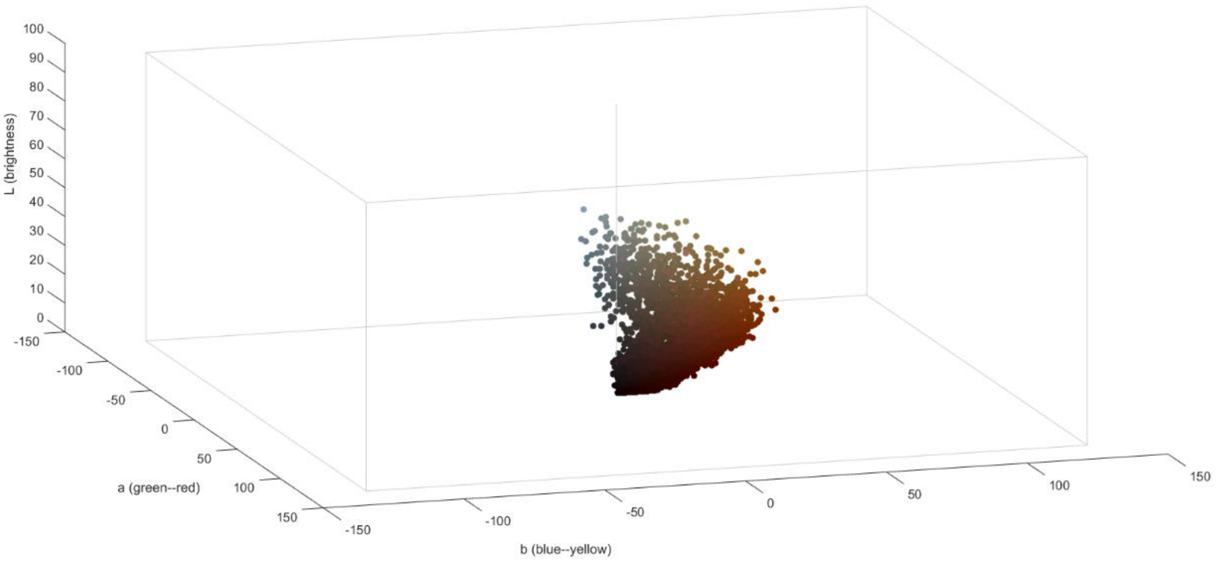

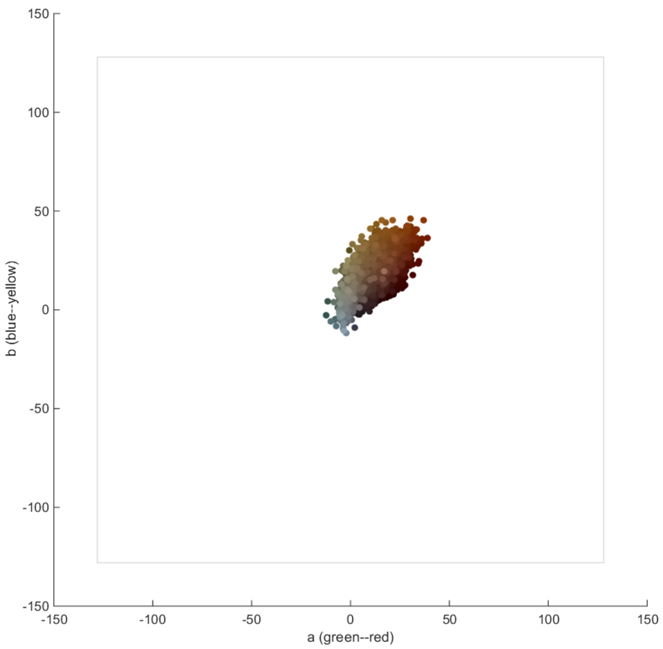

The CIE (International Commission on Illumination) Lab color space is another perceptual tristimulus colorimetric color space, using three components: L (brightness, low to high), a (color, green to red), and b (color, blue to yellow). Brightness L goes from 0 to 100, while the colors a & b are centered at 0 and range from -128 to 128, though it depends on the implementation and varies across software. The Lab color space has been used in studies of eye and hair pigmentation (35, 67, 68). The polar (cylindrical) representation of Lab color space is known as CIE LCH color space, where L remains the

same, while a & b are converted to polar coordinates: C (chroma) is the radius and H (hue) is the angle. This is very similar to the HCL color space above, though the numbers themselves are slightly different. Hue has the biggest difference: Hue in the HCL color model has three reference colors (red, green blue) at three corners while hue in CIE LCH has four reference colors (yellow, green, blue, red) at four corners.

In case of melanin-derived pigmentation, the CIE Lab color space is less appropriate than the HCL color space. That is because the natural variation in eye color is brown-light brown-gray-light blue, which is also the axis of complementary colors in the RGB / HSV / HCL color space. Thus this variation is captured well by Hue which is color angle, having nearly complementary values, and it separates hue as a color component from saturation and brightness parameters. a & b on the other hand are both components representing color, but none of them represents the brown vs. light-blue axis, since red is opposed by green in a and blue opposed by yellow in b in this color model. Thus in the above figure the data spread is diagonally along a & b, so that both variables are highly correlated in our data.

Projection Length:

Figure 3 L, M: procedure for defining the Projection Length metric.

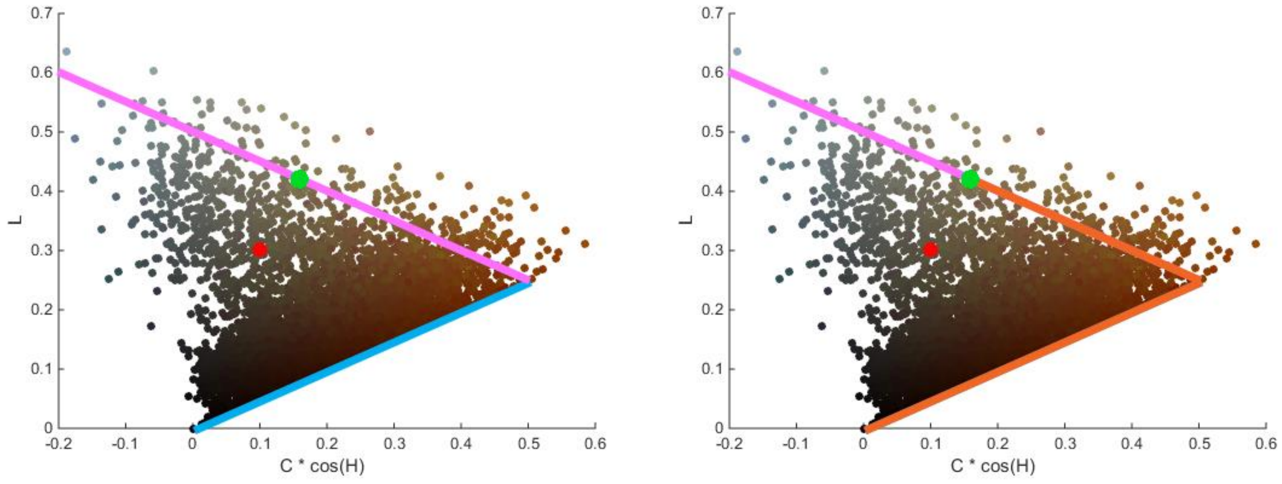

The ‘projection length’ metric is defined to approximate the T-index metric used in Beleza et al. Beleza, Johnson (1)

Because the dataset in that study consisted of various populations across the world with various degrees of admixture, the Green-Blue scatterplot consisted of multiple partially overlapping clusters with an hollow interior (Figure 2 of Beleza et al.), therefore a principal curve was constructed that fits through the clusters. In contrast, the data scatterplots in CANDELA are convex sets; for the RGB data the data cloud has a long, narrow shape (Suppl. Figure 3A-B), thus the principal curve is nearly linear and highly similar to PC1; for the HCL data the data cloud is triangular with no prominent axis of elongation (Suppl. Figure 3I, and above), and in such cases the principal curve are not very useful (69).

As the HCL scatterplot for CANDELA is approximated by a 2-dimensional convex triangle, a curve was constructed along the outside of the cluster instead, i.e. the outer two sides of the triangle. The third side of the triangle is well approximated by L where H=0 & C=0. First, the values were approximated by projecting onto a two-dimensional plane where axis 1 is C×cos(H) and axis 2 is L (see above section on HCL color space). Line 1 was constructed in this plane from (0,0) to (0.5,0.25), and line 2 was constructed from (0.5,0.25) passing through (0,0.5), the center of the HCL bicone, to extend further. Line 1 and 2 are shown in Suppl. Figure 3L using cyan and pink, respectively. Once this two-part line is constructed, rest is similar to the principal curves procedure, i.e. a projection is constructed from any point on this plane to the curve (in Suppl. Figure 3L, the projection of the red point onto this two-part line is the green point, which is the closest point on this curve to the red point), and the distance from this projected point from the origin at (0,0) is measured along the curve as the length of the two line segments (the part of the lines that contribute to the total distance is marked in orange in Suppl. Figure 3M).

Note that the lower boundary of this triangle is strict, as by construction of the HCL color space always $L \geq C\times\cos(H)$, till $L \leq 0.5$. Line 1 was chosen to represent this boundary. The other boundaries are not strict but contains the majority of the data points.

The choice of the three points representing the triangle are heuristic, but with useful mathematical properties. The origin of Line 1, (0,0), is a vertex and the lowest point of the HCL bicone, representing complete black, i.e. the highest concentration of melanin. The Other end of Line 2, (0,0.5) is the center of the HCL bicone, representing light grey, i.e. complete absence of melanin. The line can be extended further through this point to indicate blue eye colors, for which $C \times \cos(H)$ is negative.

The 'elbow' or 'inflexion point' of this two-part line was chosen to be at (0.25,0.5), which is because L at this point is at the midpoint of the other two vertices ($L=0$ & $L=0.5$), thus making the triangle isosceles, which is mathematically attractive. This makes the two lines mirror images of each other along the $L=0.5$ axis. Also, as noted above, most points lie within the two lines if this vertex was chosen.

Supplementary Figure 4: Frequency distribution of quantitative eye color variables

A) Histogram of L (Brightness)

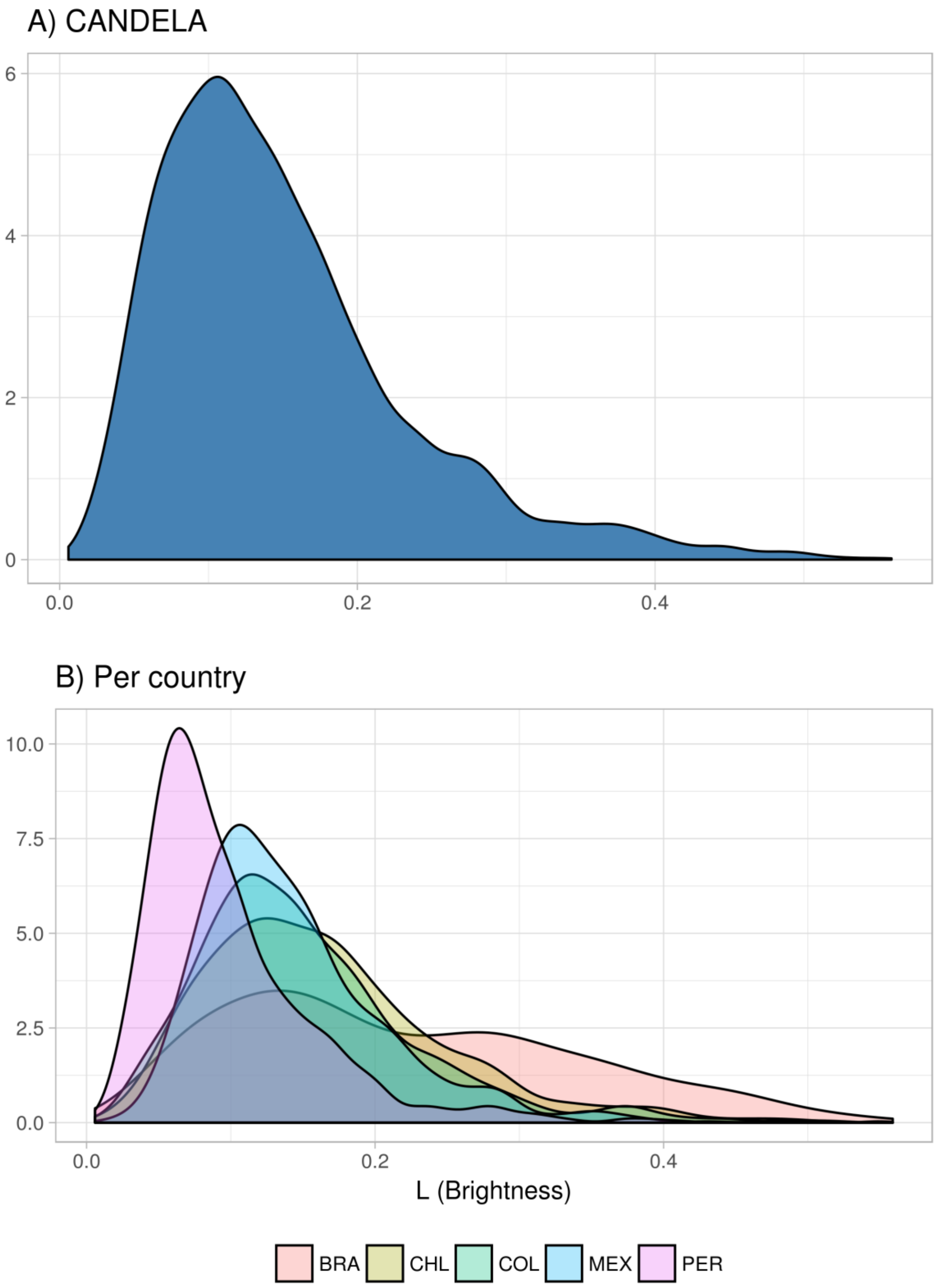

B) Histogram of C (Saturation)

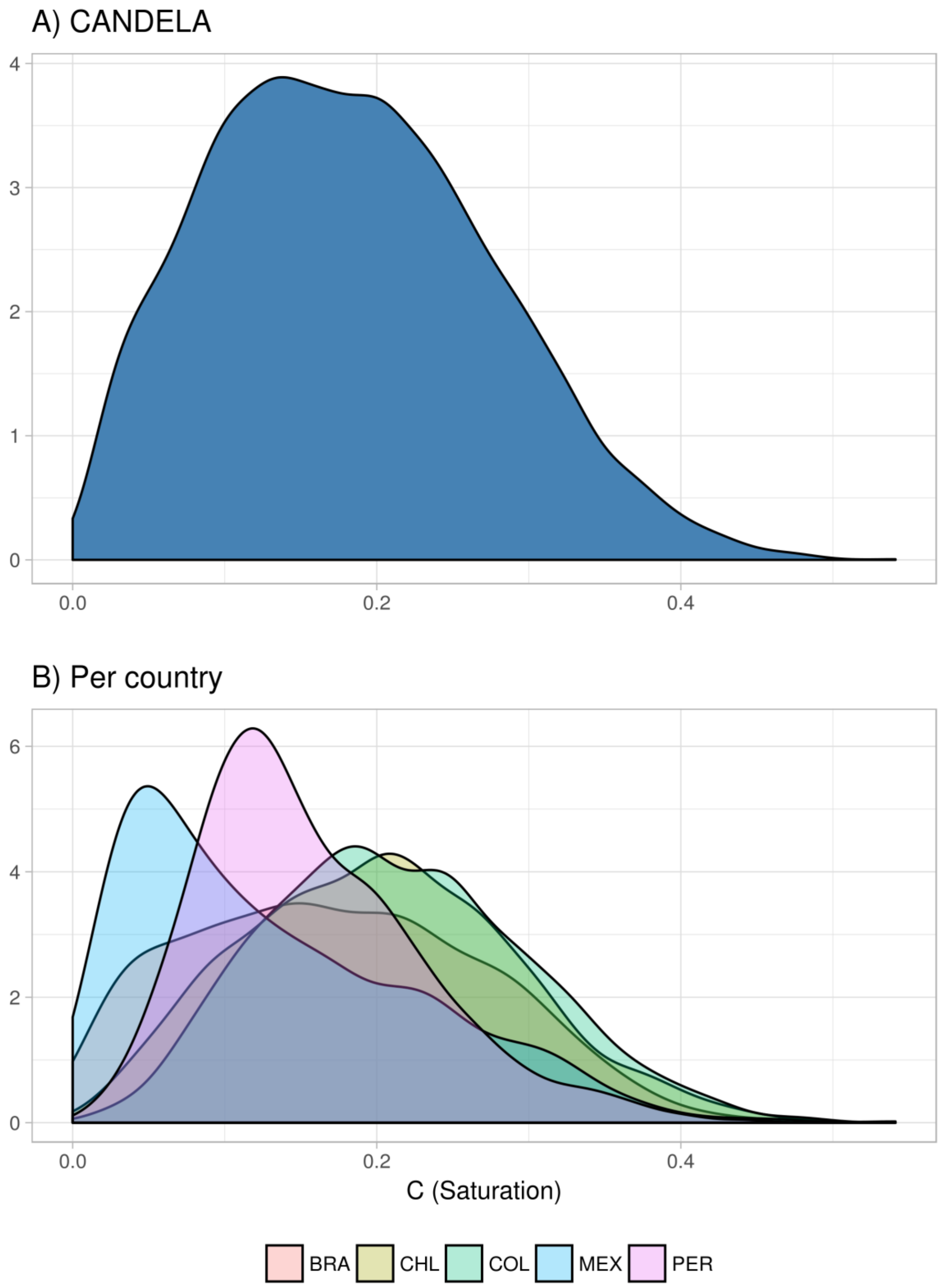

C) Histogram of cos(H) (Hue)

The complete distribution:

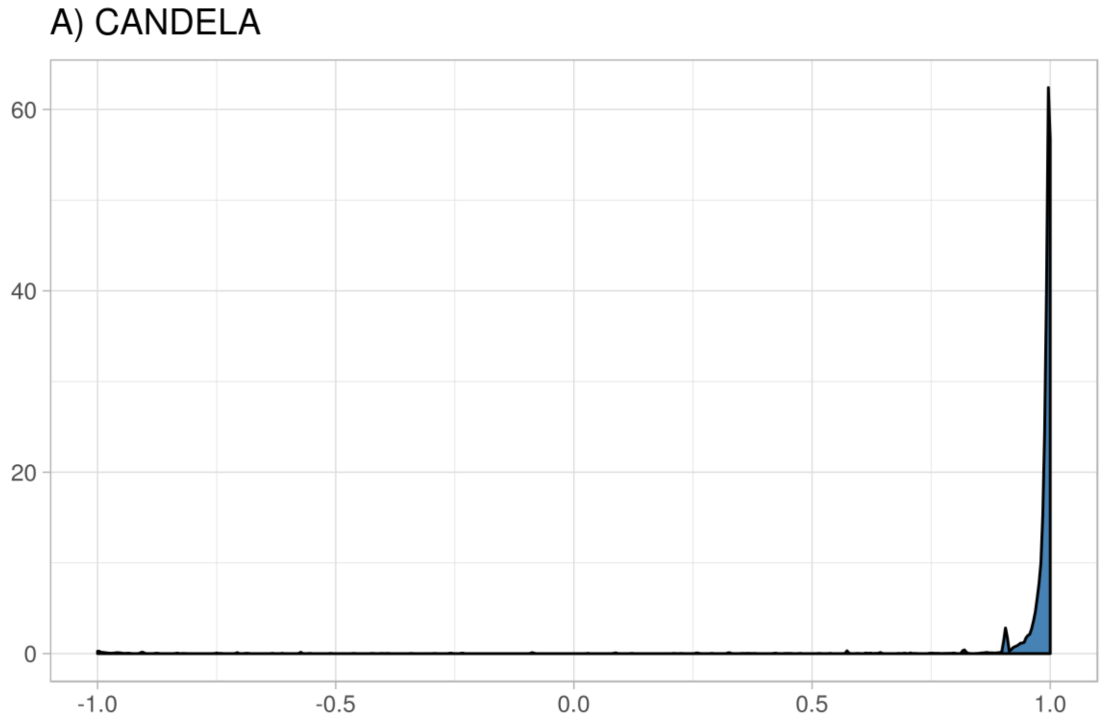

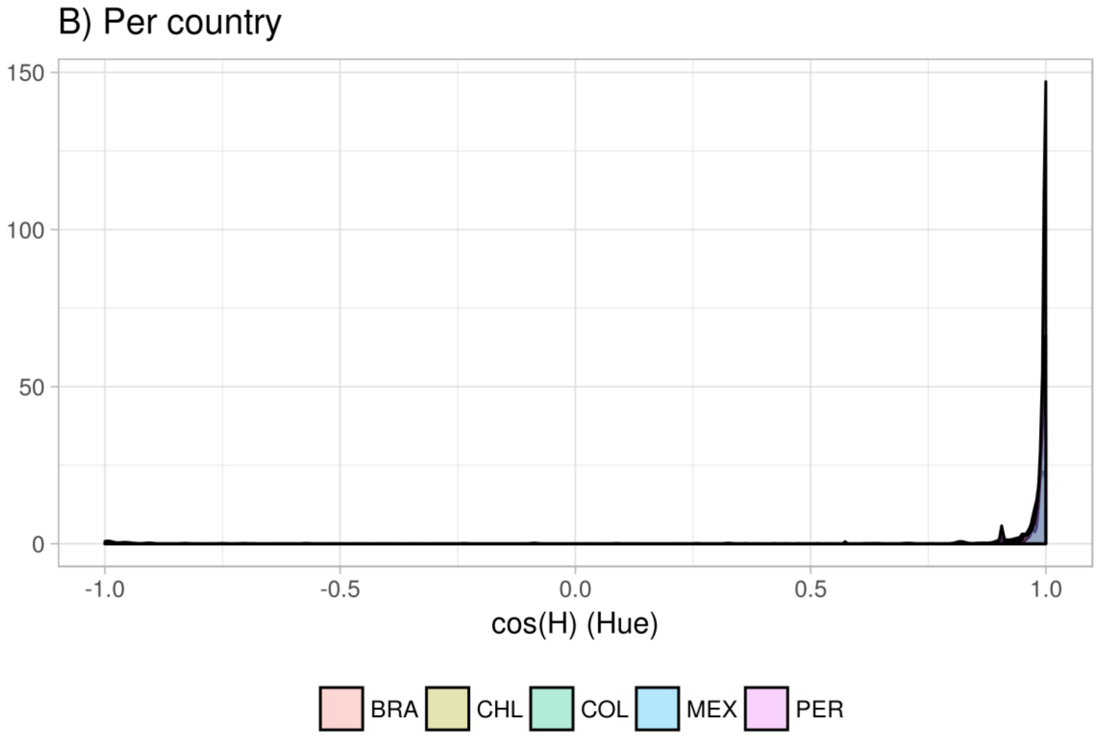

Left of the distribution:

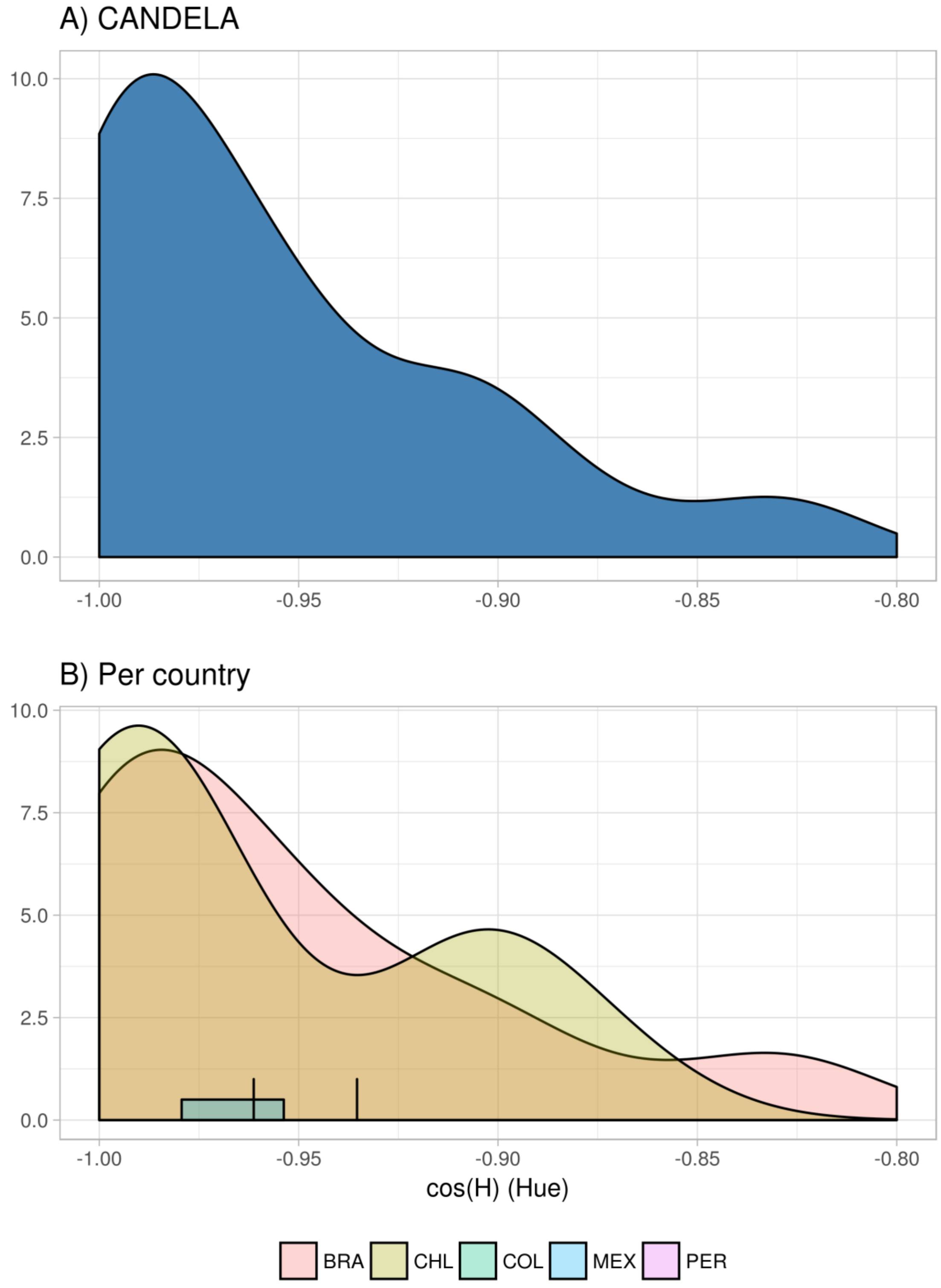

Right of the distribution:

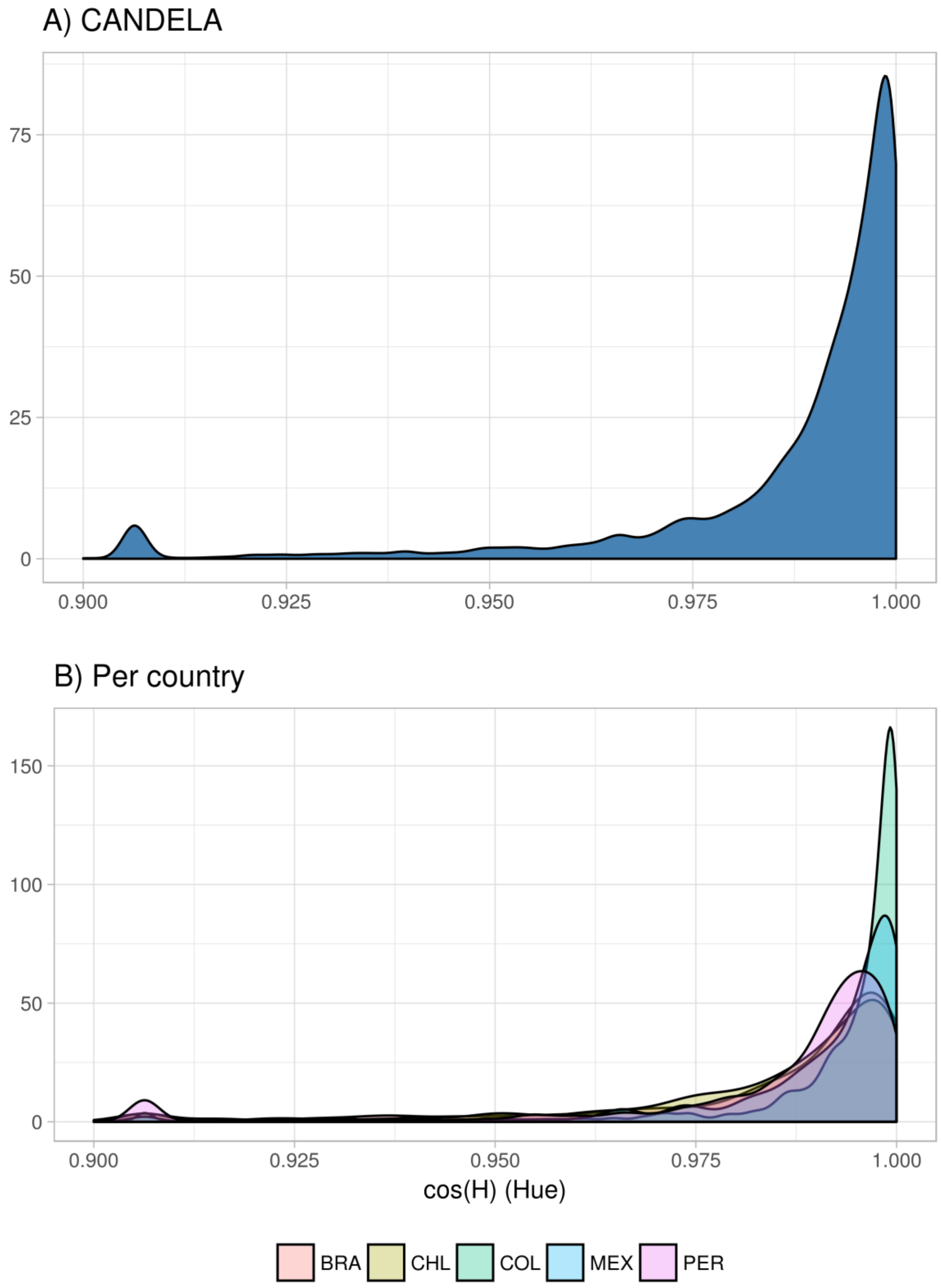

Supplementary Figure 5: Continental ancestry in the CANDELA sample

Ancestry estimates values were estimated from a set of 160,858 autosomal SNPs (LD-pruned from the full chip data) via supervised runs of the ADMIXTURE software (70). Reference populations for African, European, East Asian, and Native American groups were chosen from the 1000 Genomes Project and from selected Native American populations as described in Ruiz-Linares, Adhikari (71).

Individual ancestry barplots for each country are shown below. Individuals within each country are sorted by their European ancestry proportion.

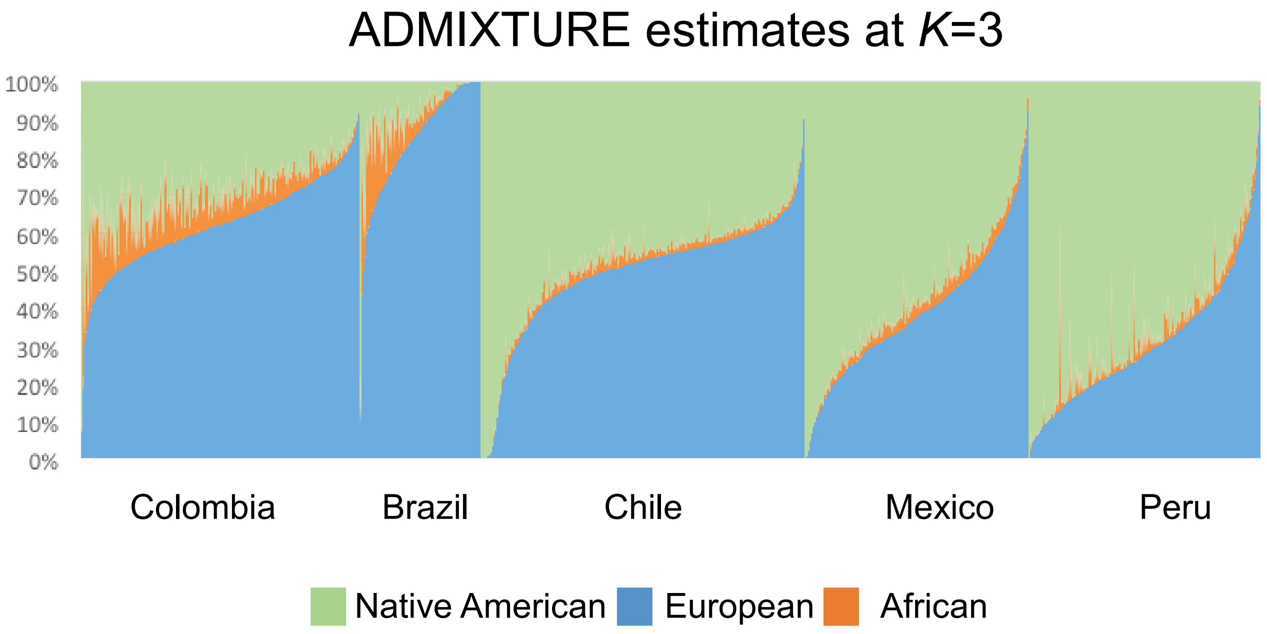

Mean ancestry estimates for each country and overall are:

	African	European	Native American
Colombia	9.6%	61.2%	29.2%
Brazil	9.3%	78.6%	12.1%
Chile	4.6%	46.2%	49.3%
Mexico	4.8%	37.7%	57.5%
Peru	4.6%	30.6%	64.8%
CANDELA	6.2%	48.2%	45.6%

Ancestry estimates from supervised ADMIXTURE run at K=4 is shown below, with the additional East Asian component.

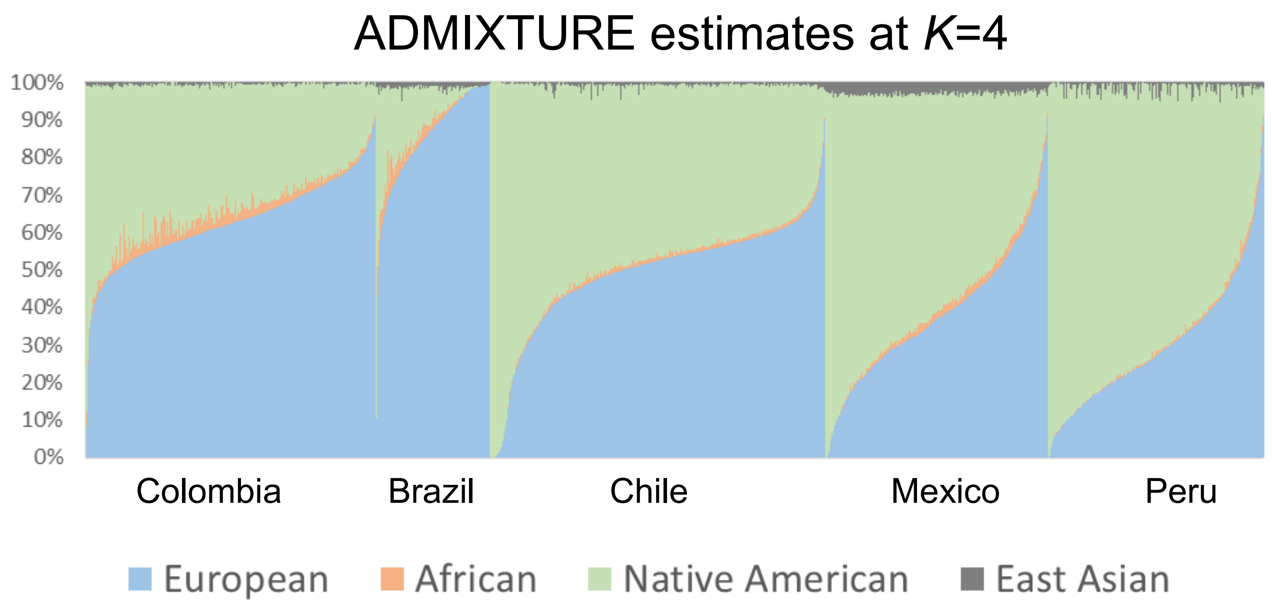

Exclusion criteria based on ancestry:

- 185 samples had >5% East Asian ancestry and these were excluded from the GWAS analysis.
- A total of 188 individuals with high African ancestry were removed. By examining the long thin tail of the distribution of individual African ancestry values, we decided to use the upper 2.5% quantile as the threshold, which equates to 20% ancestry.

Supplementary Figure 6: Selection of genetic Principal Components for inclusion in the GWAS analyses

A) Scree plot

Principal components were extracted from an LD-pruned SNP dataset (see Methods section in main text). The proportion of variance explained by each PC is shown below.

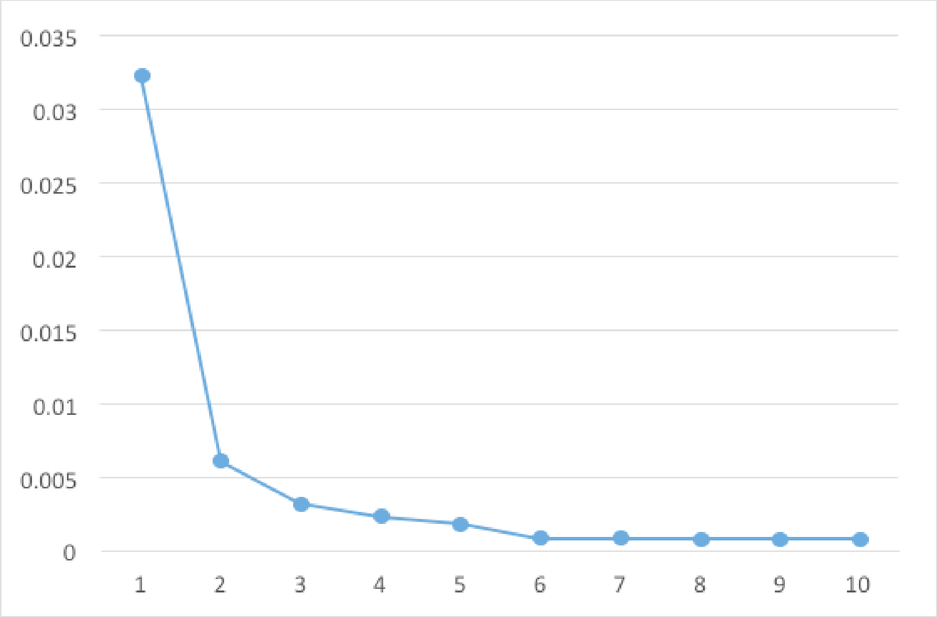

B) GWAS Q-Q plots

Skin pigmentation (Melanin Index)

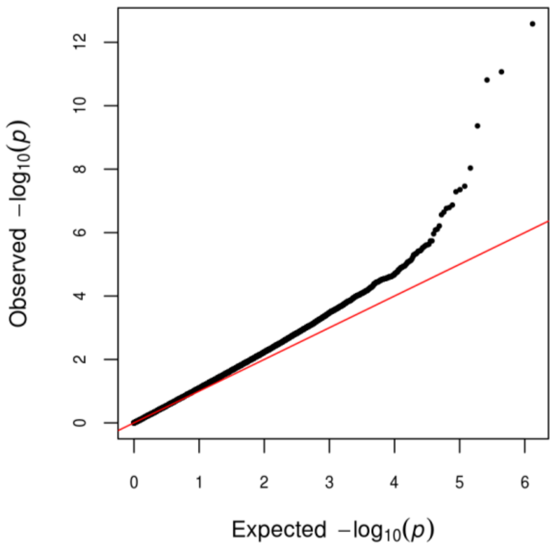

Hair color (categorical)

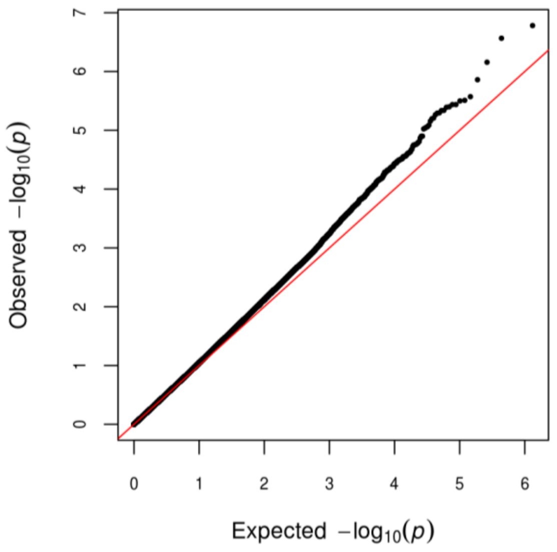

Eye color (categorical)

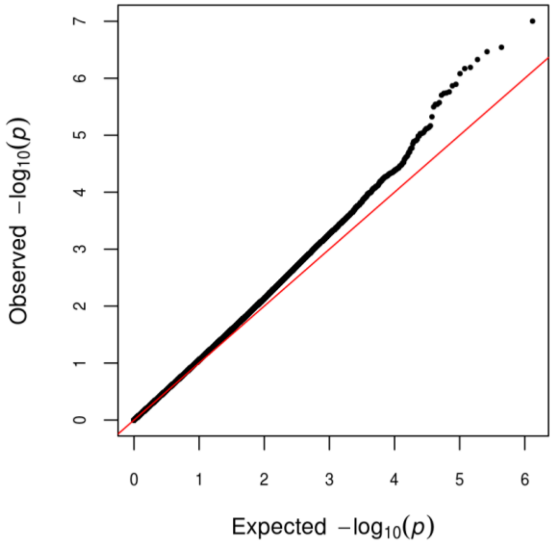

L (Brightness)

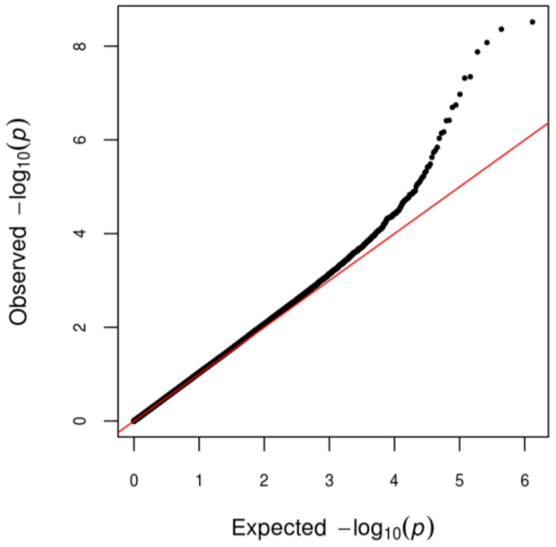

C (Saturation)

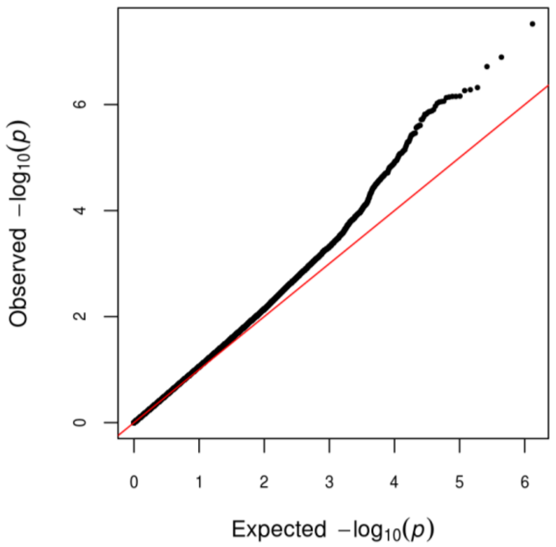

cos(H) (Hue)

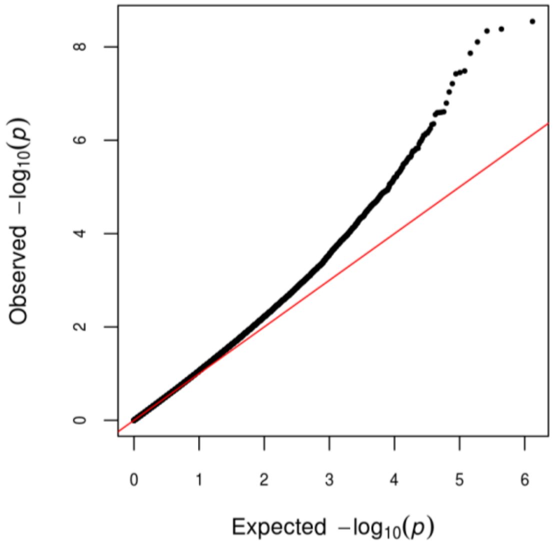

Supplementary Figure 7: Regional association plots of genome-wide significant SNPs associated to pigmentation traits

Regional association plots for each index SNP in Table 1 (except novel SNPs already shown in Figure 6). LocusZoom plots are given below. For simplicity, only the trait with most significant P-value is shown.

A) rs16891982 – 5p13 (SLC45A2) – Skin pigmentation (MI)

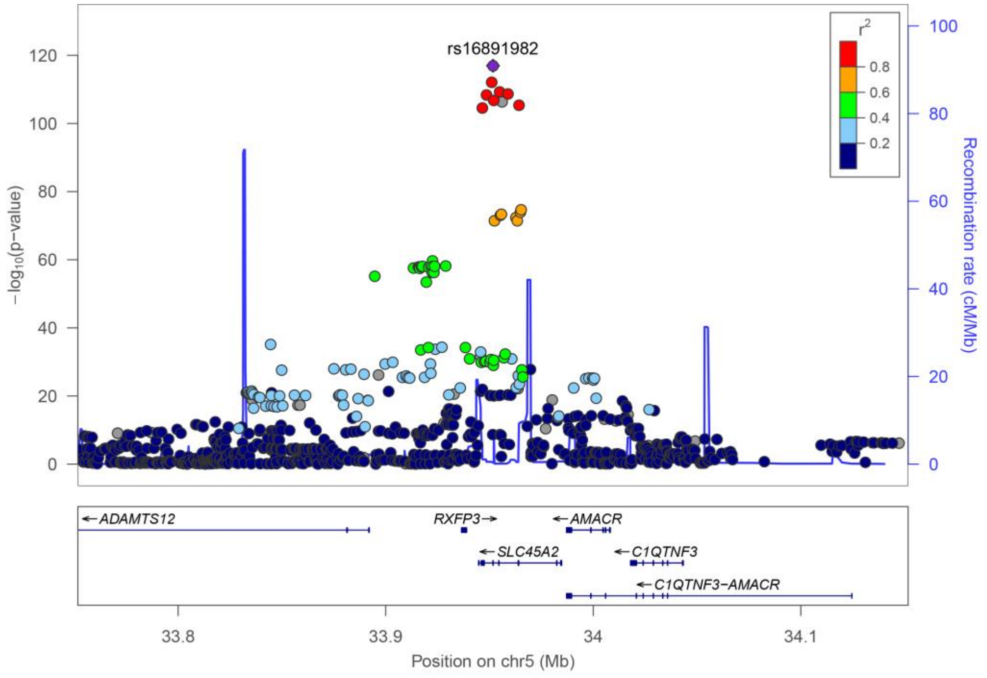

B) rs12203592 – 6p25 (IRF4) – L (Brightness)

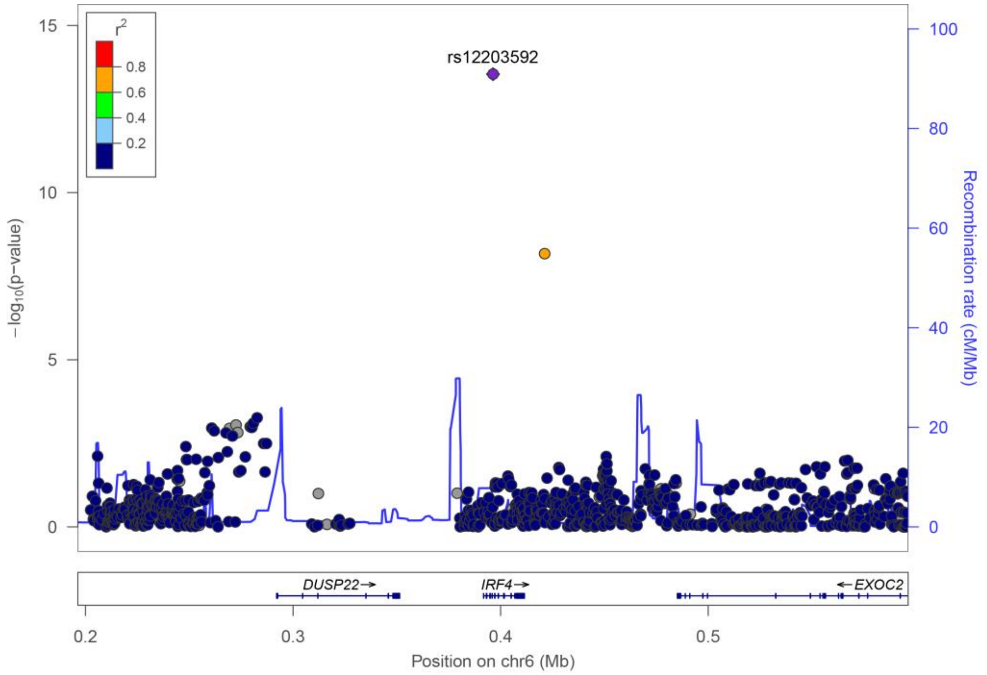

C) rs10809826 – 9p23 (TYRP1) – L (Brightness)

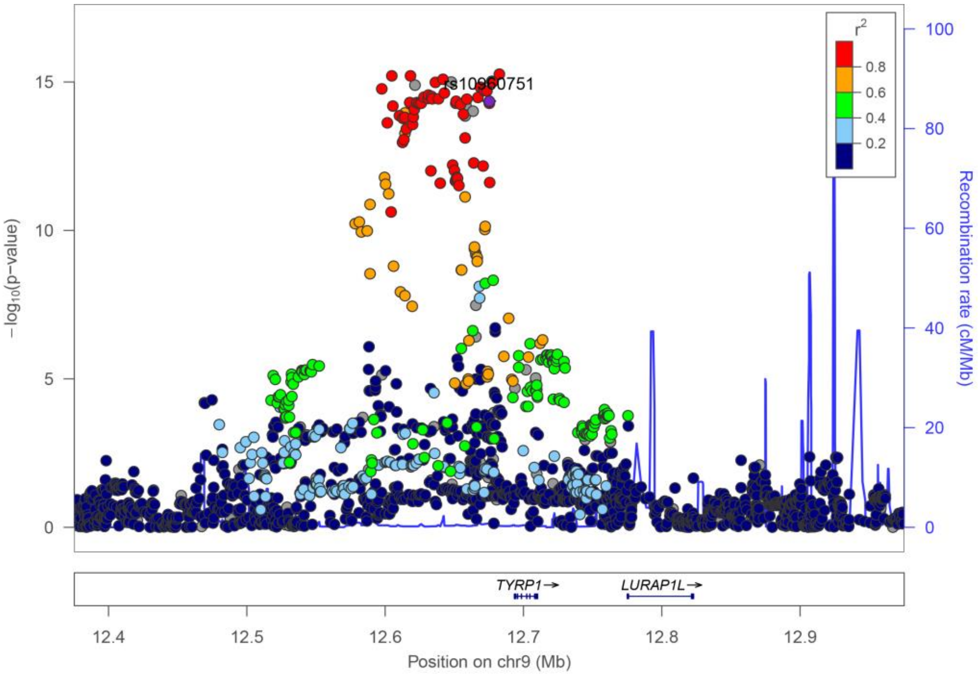

D) rs7118677 – 11q14 (GRM5) – Skin pigmentation (MI)

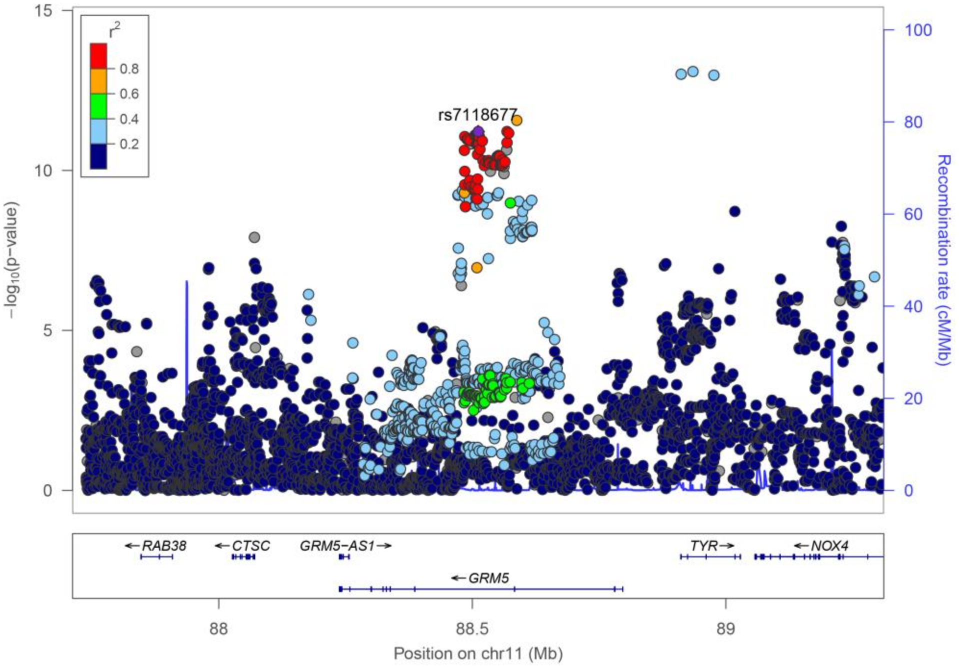

E) rs1042602 – 11q14 (TYR) – Skin pigmentation (MI)

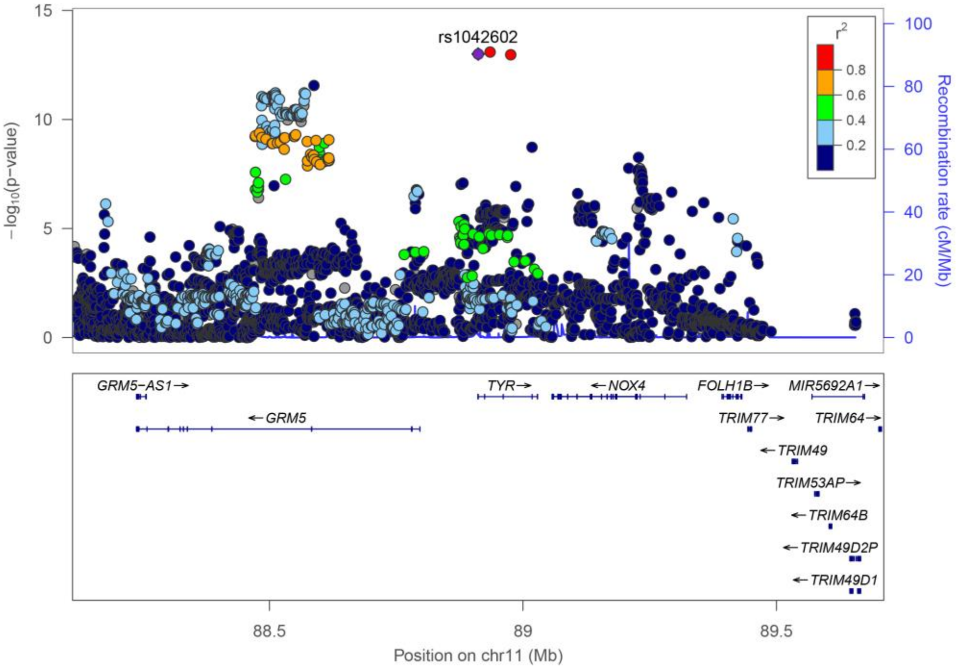

F) rs1126809 – 11q14 (TYR) – Skin pigmentation (MI)

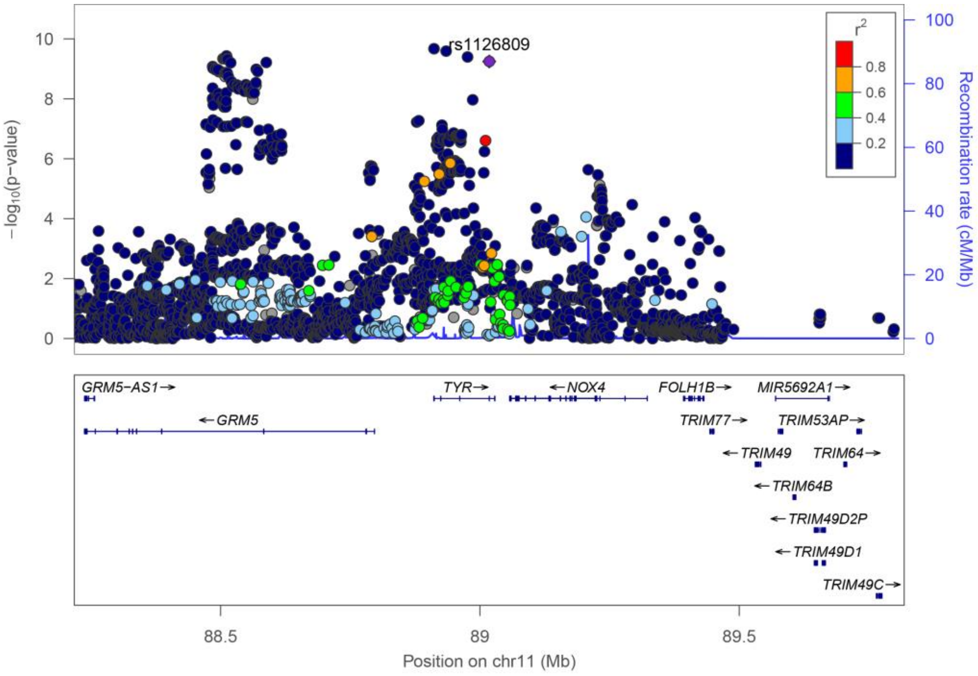

G) rs4778219 – 15q13 (OCA2) – L (Brightness)

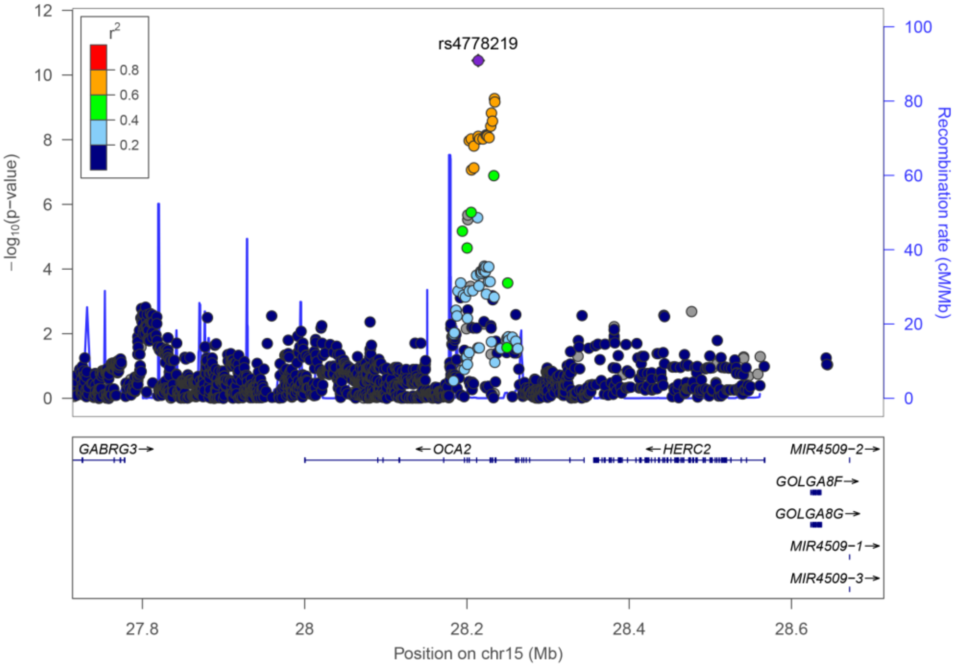

H) rs1800407 – 15q13 (OCA2) – L (Brightness)

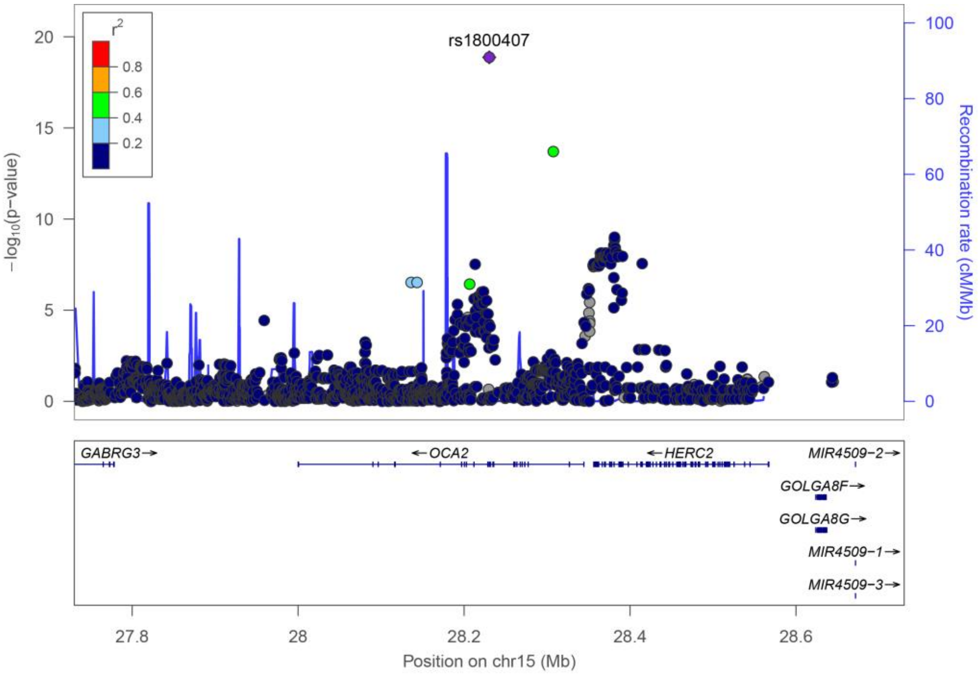

I) rs1800404 – 15q13 (OCA2) – Skin pigmentation (MI)

(Although L has stronger P-values for this SNP, the plot for L is difficult to see clearly due to the very high peak from HERC2 nearby, as seen below)

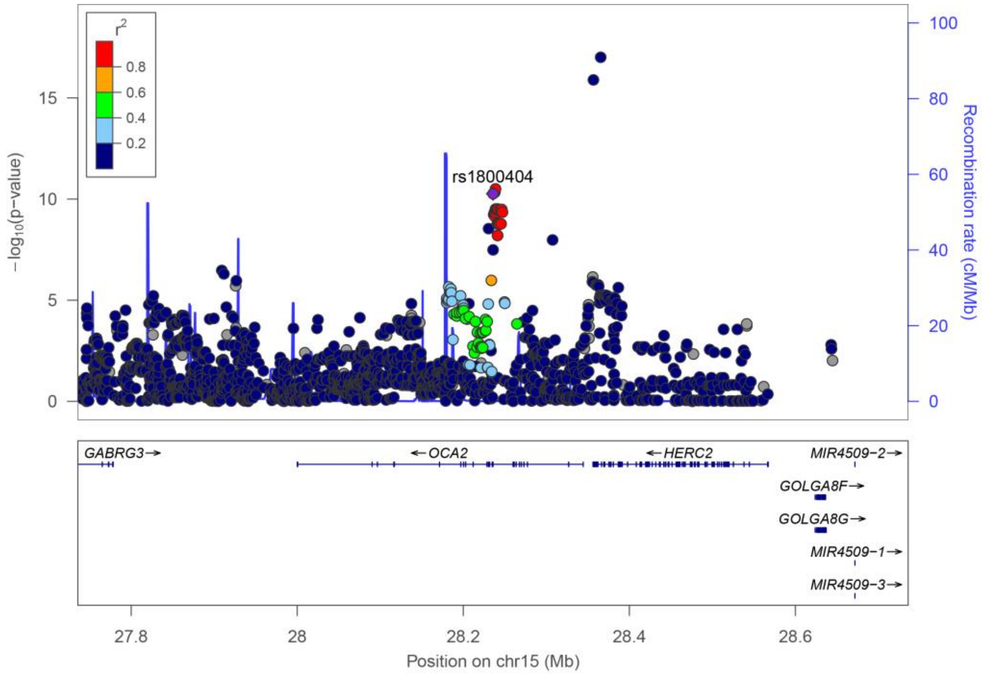

J) rs12913832 – 15q13 (HERC2) – L (Brightness)

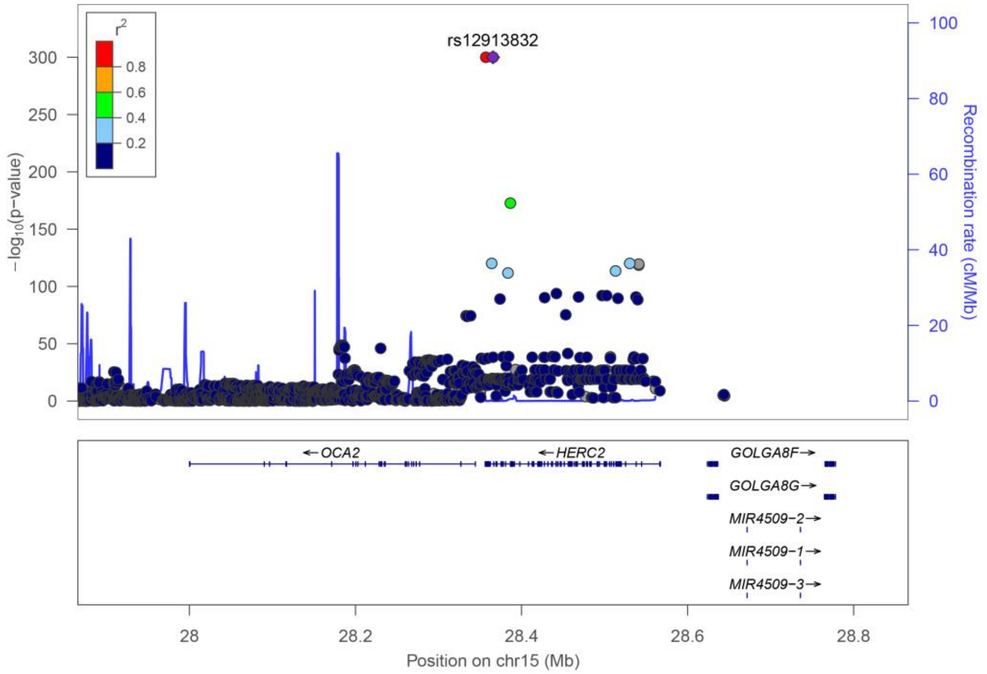

K) rs4778249 – 15q13 (HERC2) – C (Saturation)

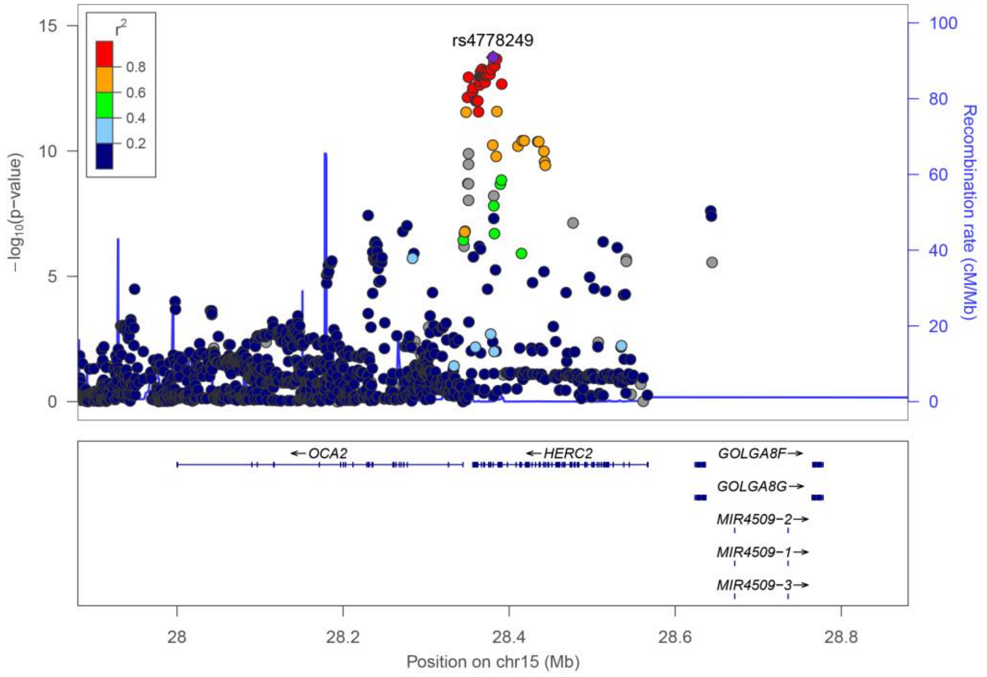

L) rs1426654 – 15q21 (SLC24A5) – Skin pigmentation (MI)

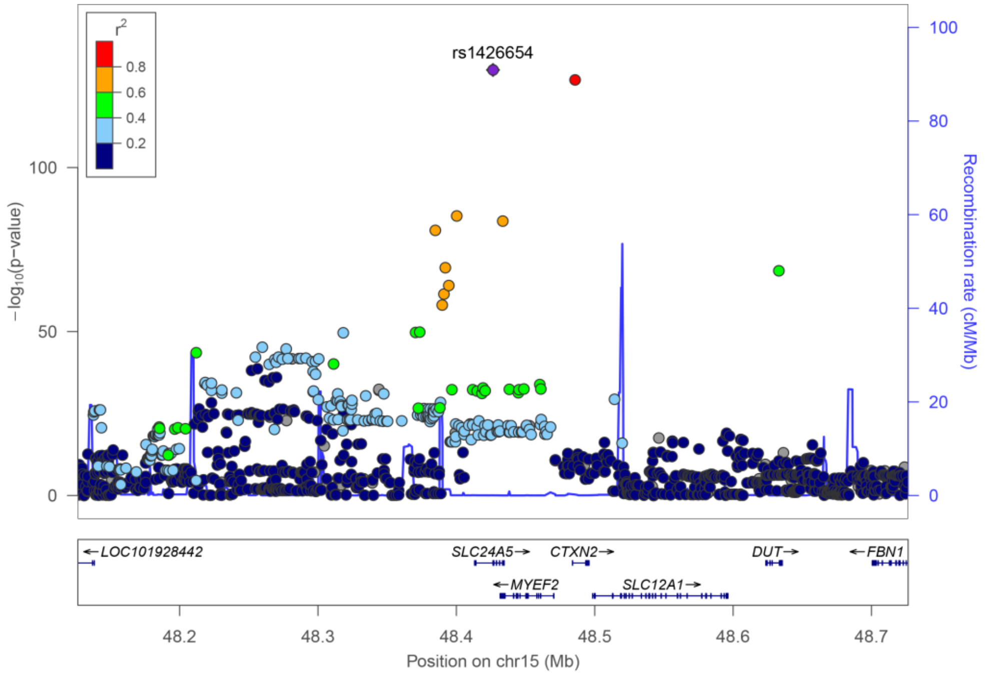

M) rs885479 – 16q24 (MC1R) – Skin pigmentation (MI)

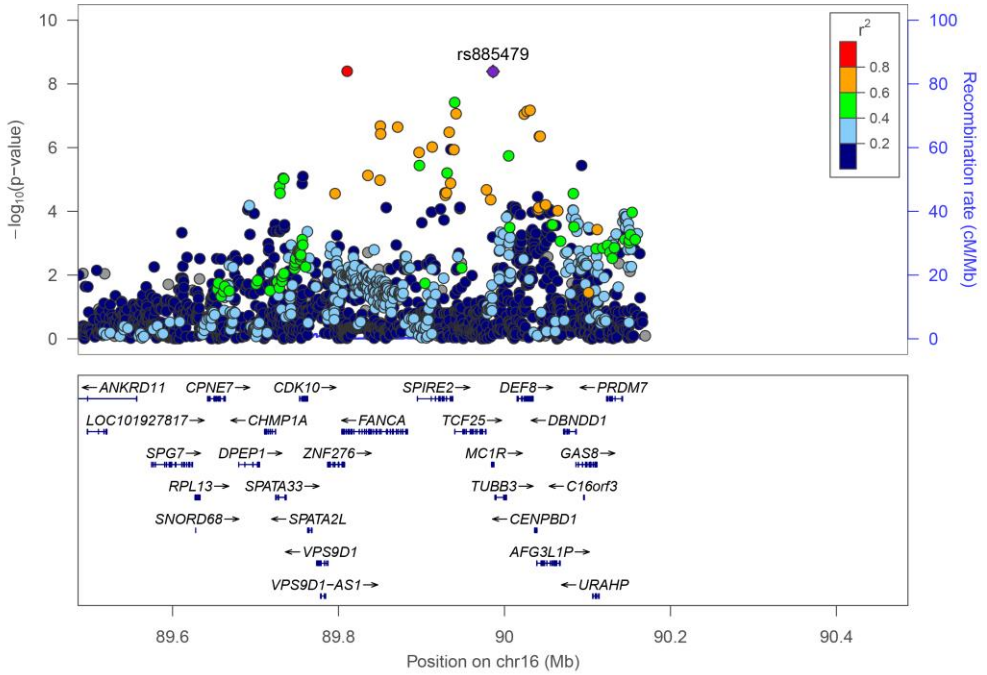

Supplementary Figure 8: Meta-analysis for 6 index SNPs representing novel associations to pigmentation traits

Results for Melanin Index (MI), L (Lightness/Brightness), C (Chroma/Saturation) and cos(H) (Hue/Tone) are shown top to bottom. Panels **a-b, d, f-g, i** show forest plots for the index SNPs in each country sample (blue) and in a combined meta-analysis (red). Boxes indicate allelic effects, with box size proportional to sample size and horizontal lines representing confidence intervals of 2 standard errors. Significant effects are shown in dark blue. On the right is shown the frequency distribution for each trait in all samples (panels **c, e, g, h, j**). For Cos(H), the proportion of negative values is small (i.e. blue-grey eyes are relatively rare in the CANDELA sample). We therefore also show a histogram restricted to the negative values of cos(H) in panel **k.** This panel highlights that blue/grey eyes are mostly seen in Brazil, consistent with the significant allelic effect detected only in the sample from that country **(i)**.

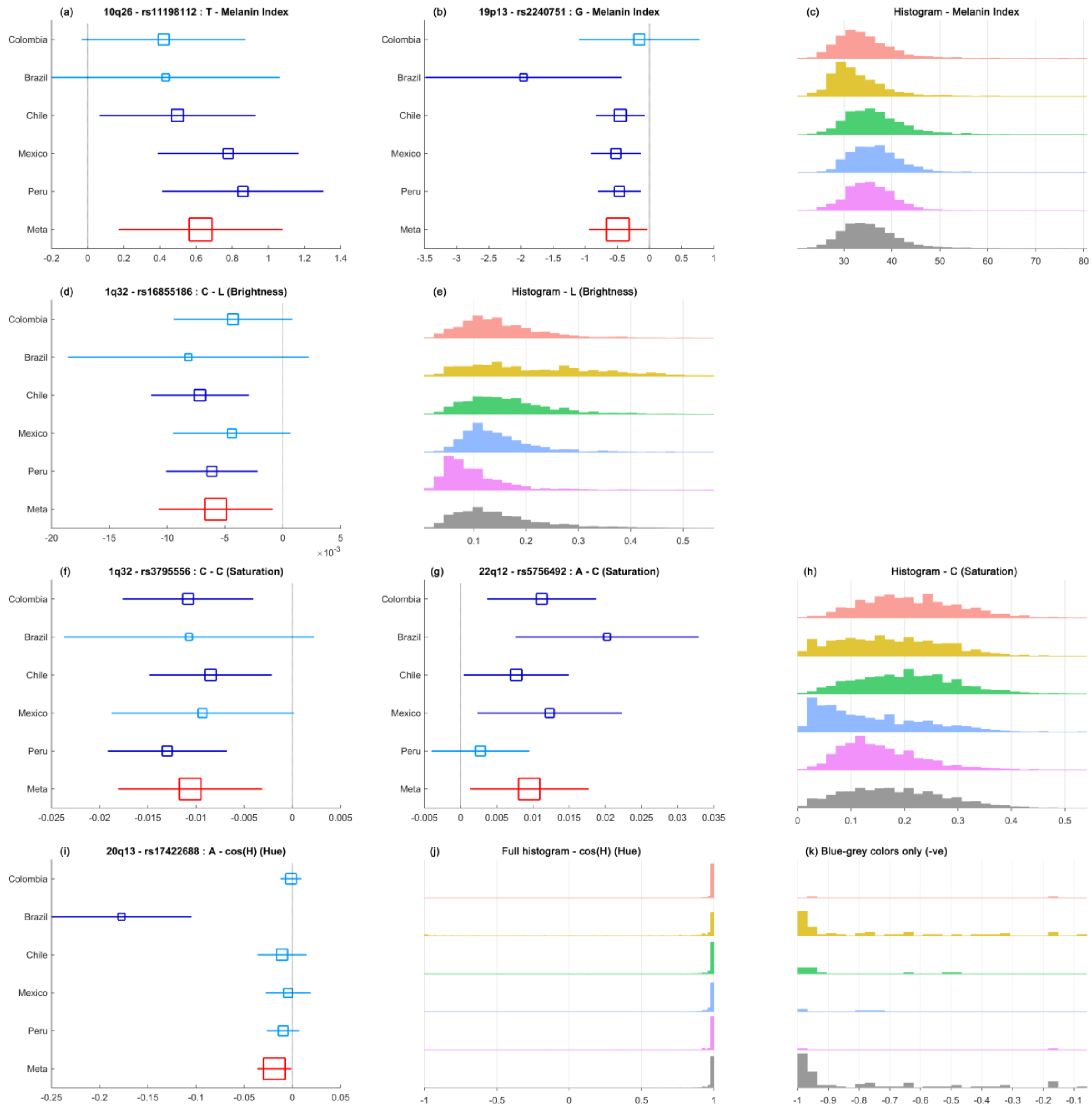

Supplementary Figure 9: Genomic annotation in the 10q26 intergenic region around rs11198112

A UCSC genome-browser screenshot from the (GRCh37/hg19) assembly for the 10q26 intergenic region around rs11198112 (highlighted in black at the bottom), our index SNP. SNP rs11198112 is included in the binding site for EBF1 (Early B-cell factor) transcription factor. Additionally, this region shows enriched values of PhyloP and GERP conservation scores.

Full view:

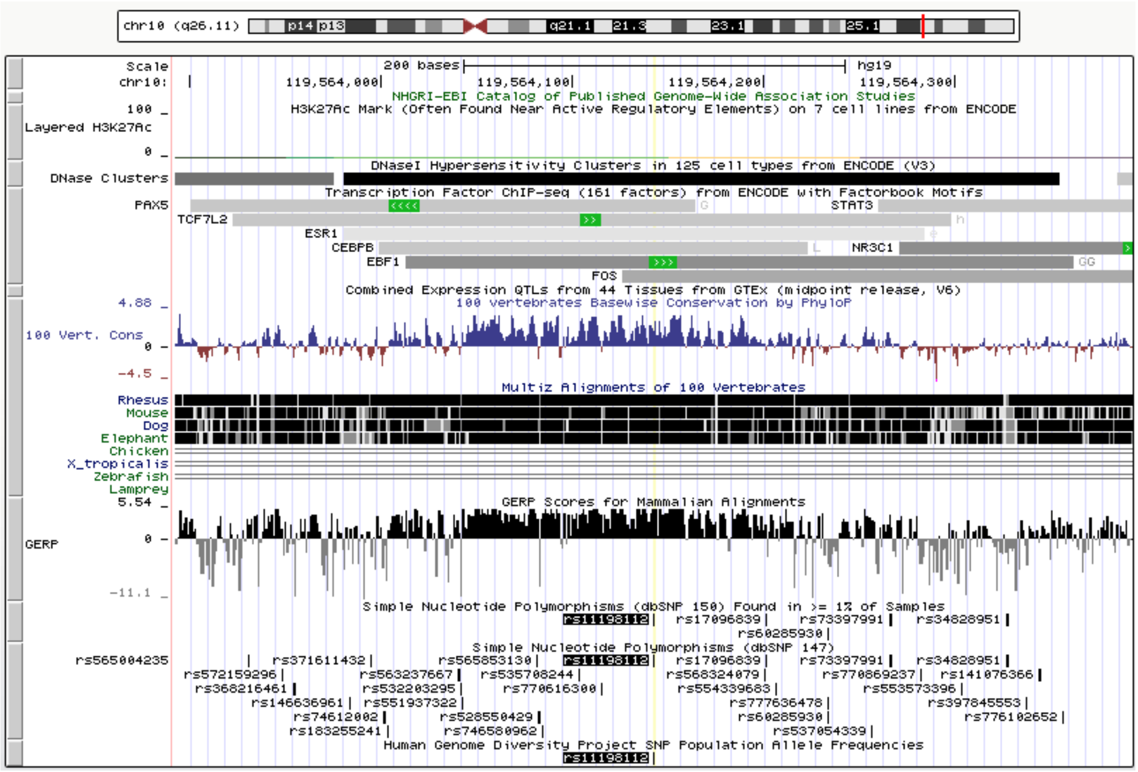

Zoomed in view:

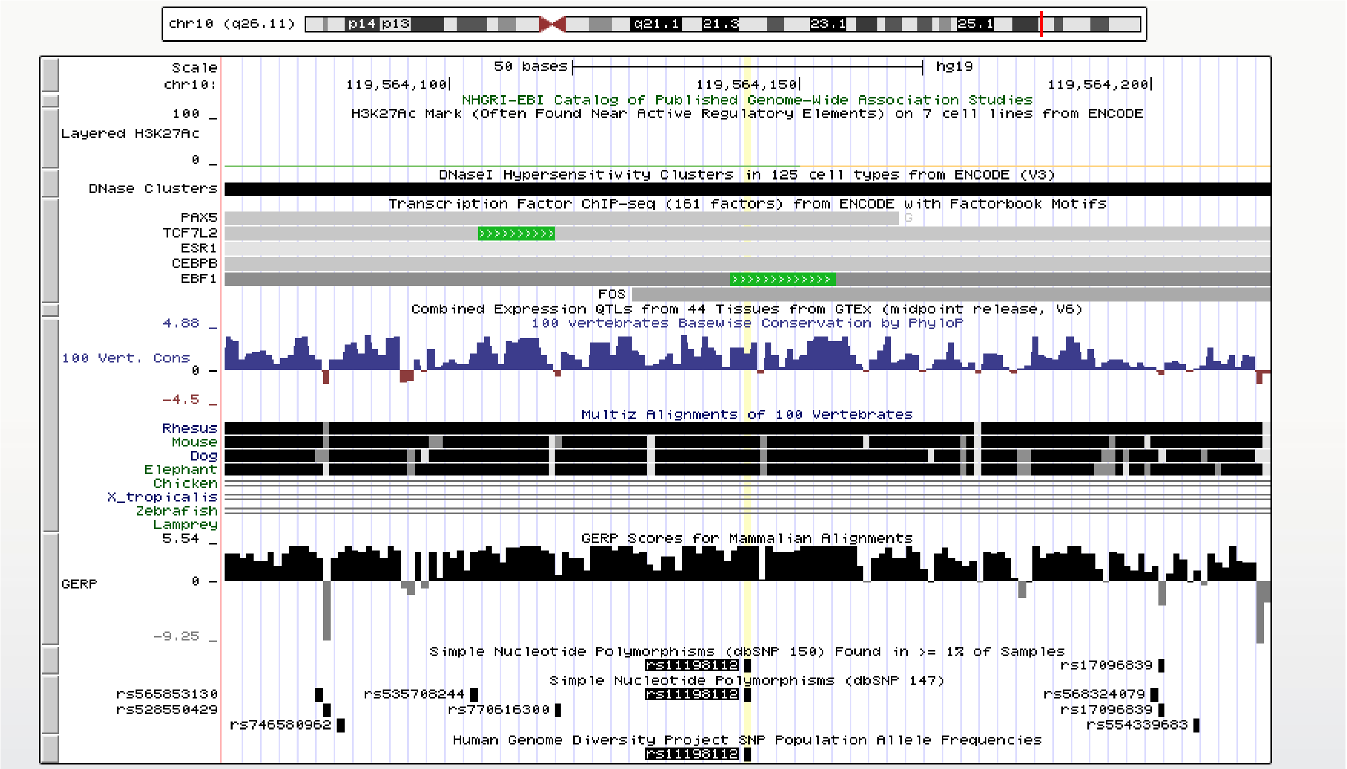

Supplementary Figure 10: Worldwide allele frequency of rs11198112

The allele frequency for SNP rs11198112 was retrieved from the Allele Frequency Database (ALFRED) via https://alfred.med.yale.edu/alfred/. This database includes allele frequency information for 84 worldwide populations. The colors of the bars reflect the major geographic origin of the populations as categorized by ALFRED: Africa (green), Europe (blue), Middle East and North Africa (brown), Asia (orange), East Asia (purple), Siberia (grey), America (red) and Oceania (yellow). The number of individuals per populations (N) is given next to the population name and the derived allele frequency is displayed on the top of each bar.

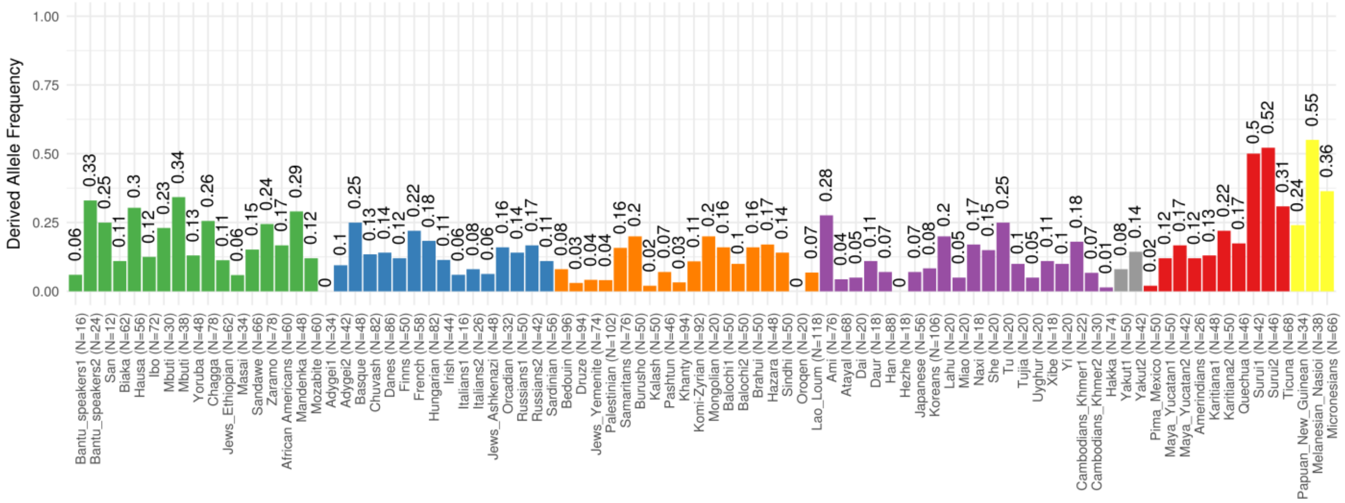

Supplementary Figure 11: Worldwide allele frequencies of rs2240751 (MFSD12)

The allele frequencies are estimated from 2391 unrelated individuals from a worldwide dataset including 64 native populations (Supplementary Table 10). The colors of the bars reflect the geographic origin of the populations: Africa (green), Europe (blue), Middle East and North Africa (brown), East Asia (purple), South Asia (pink), South East Asia (orange), Siberia (grey), America (red) and Oceania (yellow). The number of individuals per populations (N) is given next to the population name and the derived allele frequency is displayed on the top of each bar.

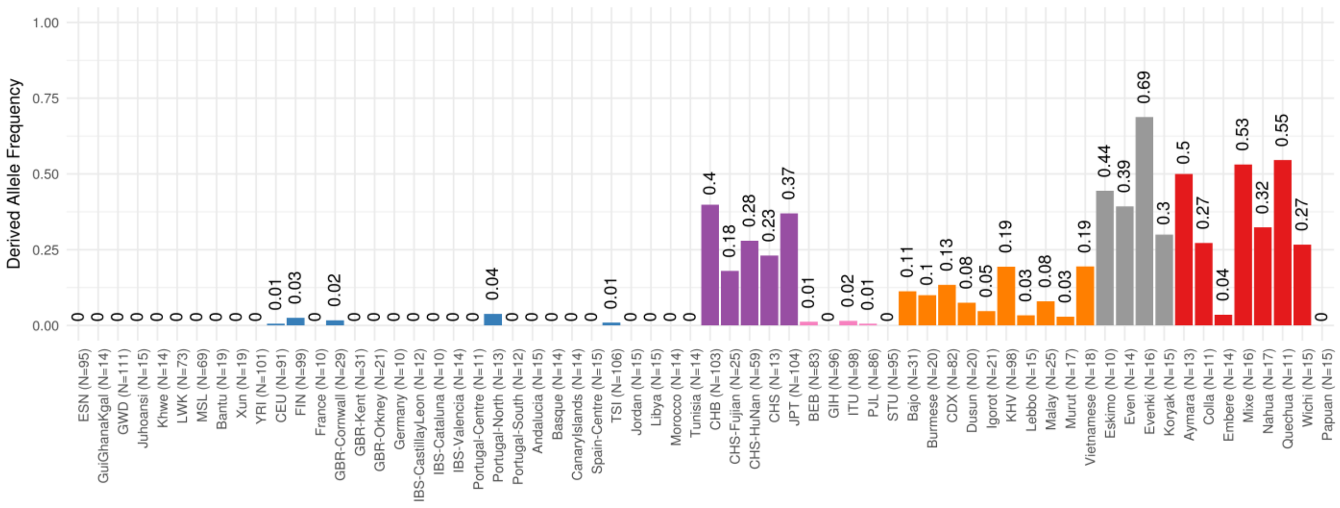

Supplementary Figure 12: Cross-tissue expression of novel candidate genes of pigmentation traits in the GTEx database

Expression box plots across all 53 tissues that have RNA sequencing data in the GTEx database for our pigmentation associated novel candidate genes (Table 1A). The boxplot displays the median, 25th and 75th percentiles of the expression values in each tissue. Circles are displayed as outliers if they are above or below 1.5 times the interquartile range. RPMK refers to reads per kilobase per million. In all panels, tissues are ordered according to the median expression values in descending order. Black arrows point to skin cell types.

A) 1q32 DSTYK

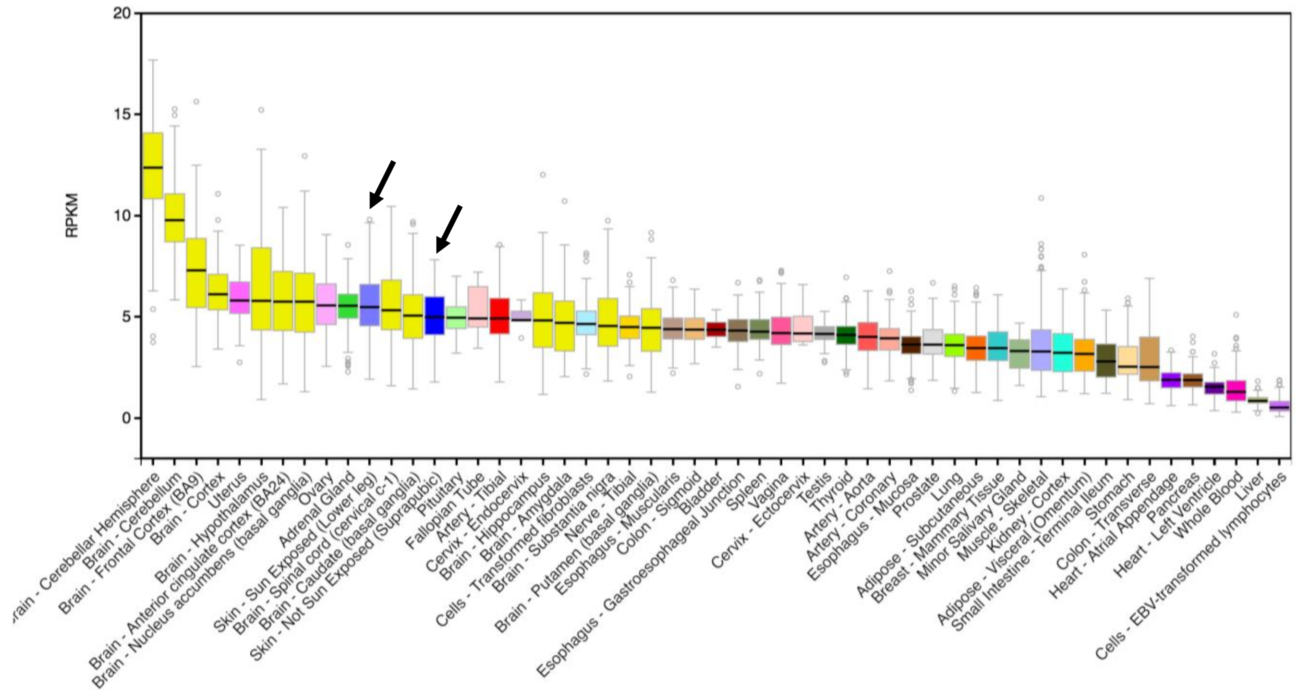

B) 10q26 EMX2

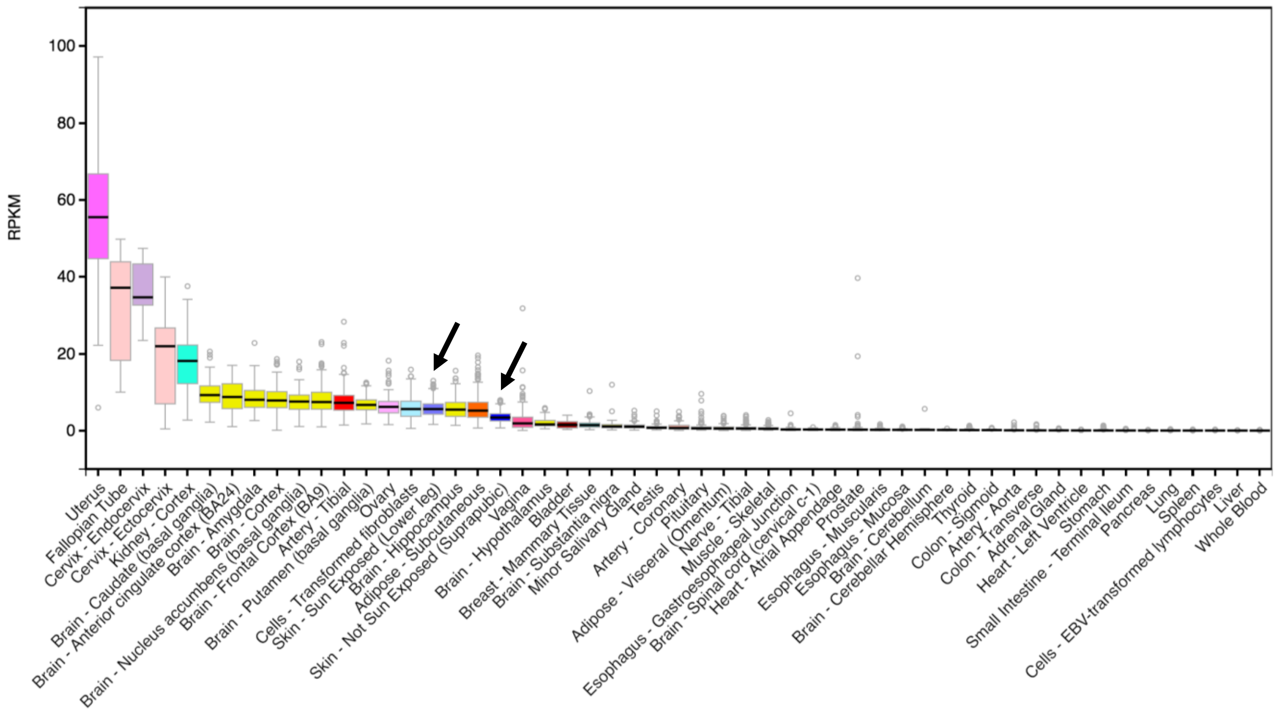

C) 19p13 MFSD12

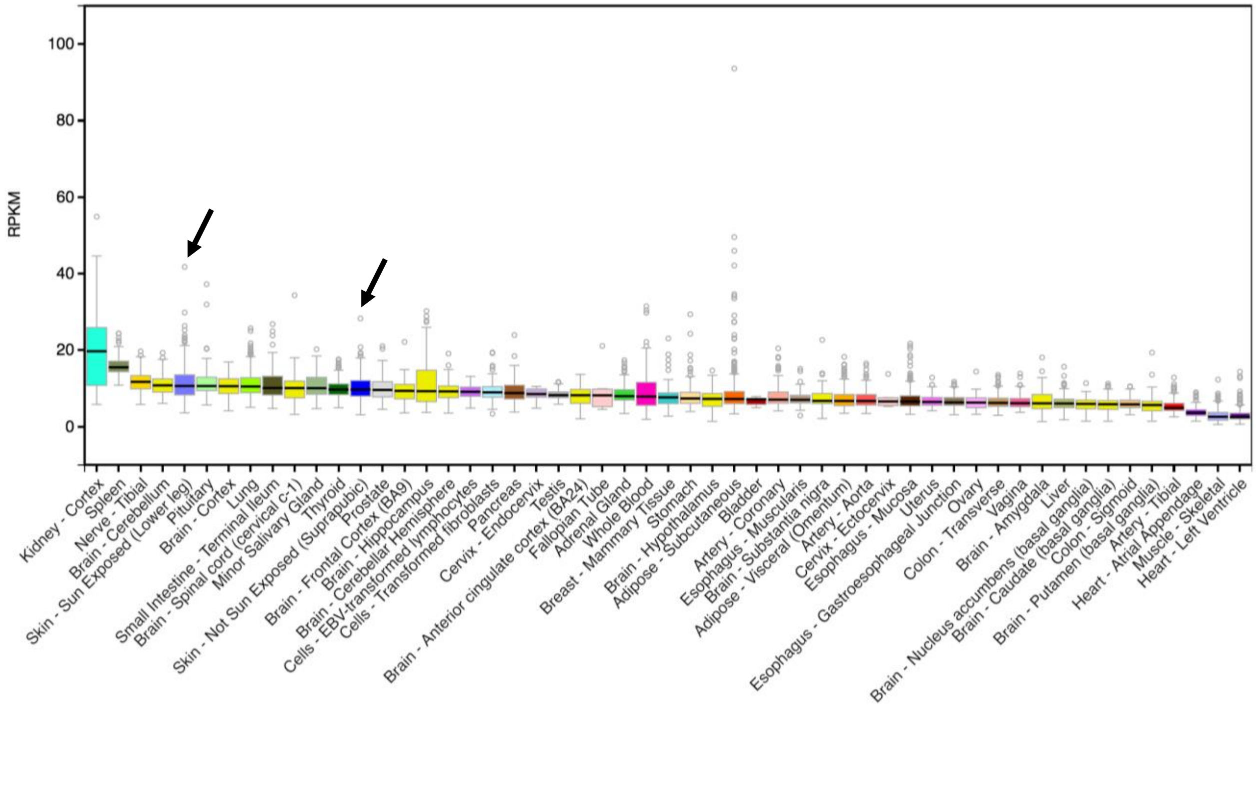

D) 20q13 WFDC5

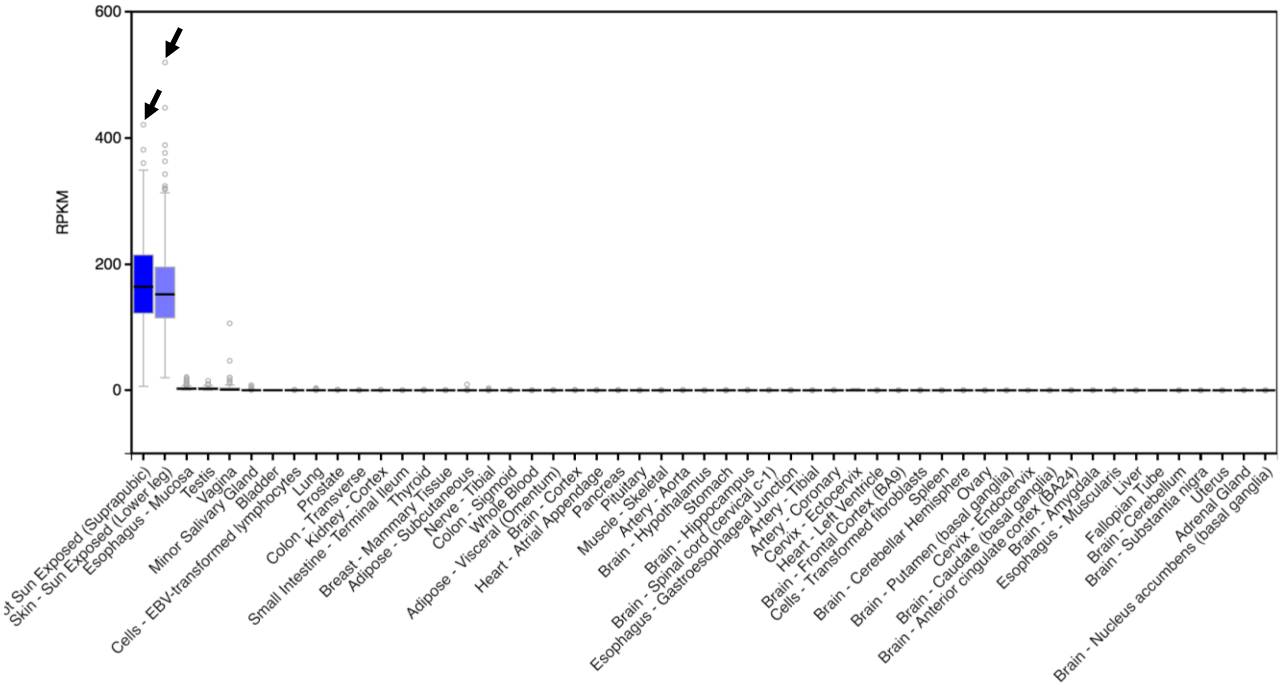

E) 22q12 MPST

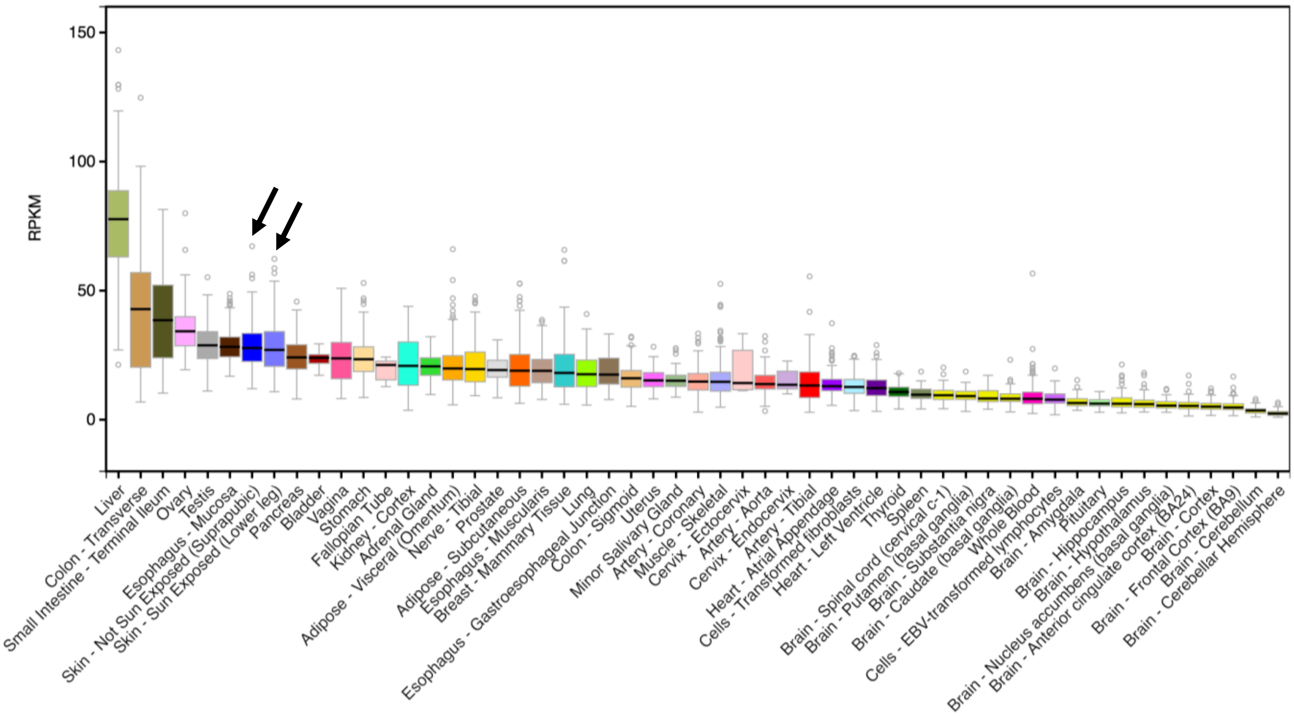

Supplementary Figure 13: Distribution of selection statistics scores surrounding index SNPs

We computed three selection statistics at regions surrounding the index SNPs in the CHB and CEU populations from the 1000 Genomes Project: the integrated Haplotype Score (iHS), Tajima's D and the Population Branch Statistic (PBS). Note that depending on the selection statistic, it is not possible to assign a selection score to each SNP (see Methods), and therefore the number of SNPs with assigned scores may differ between selection tests. At each plot the dashed black line corresponds to the 99th percentile of the selection statistic and additionally the 1st percentile for Tajima's D. If present, the index SNP is highlighted in purple and its position represented with a dashed vertical line. The bottom plot shows the genes at the specific genomic region.

A) 1q32 – rs3795556

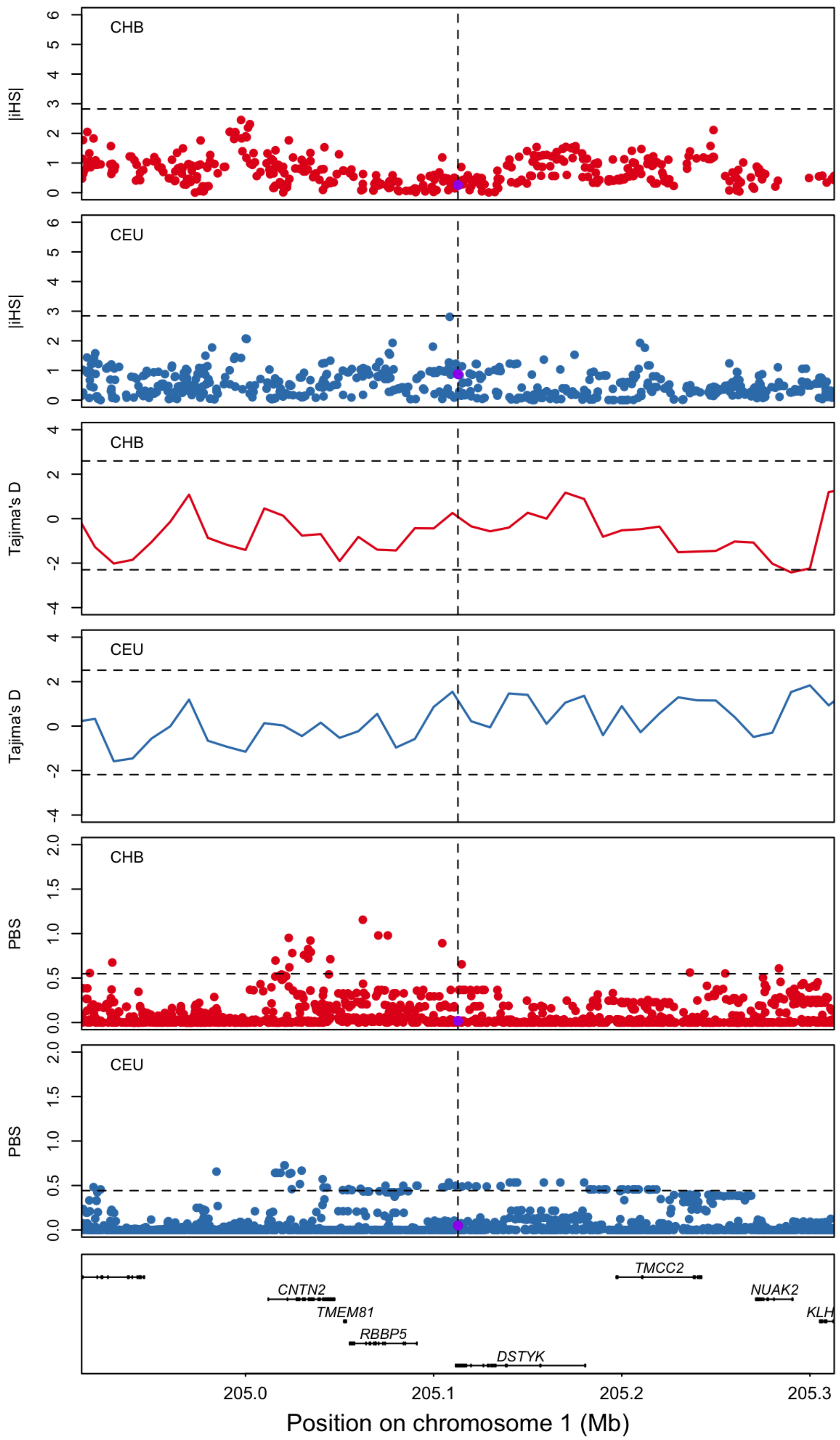

B) 5p13 – rs16891982

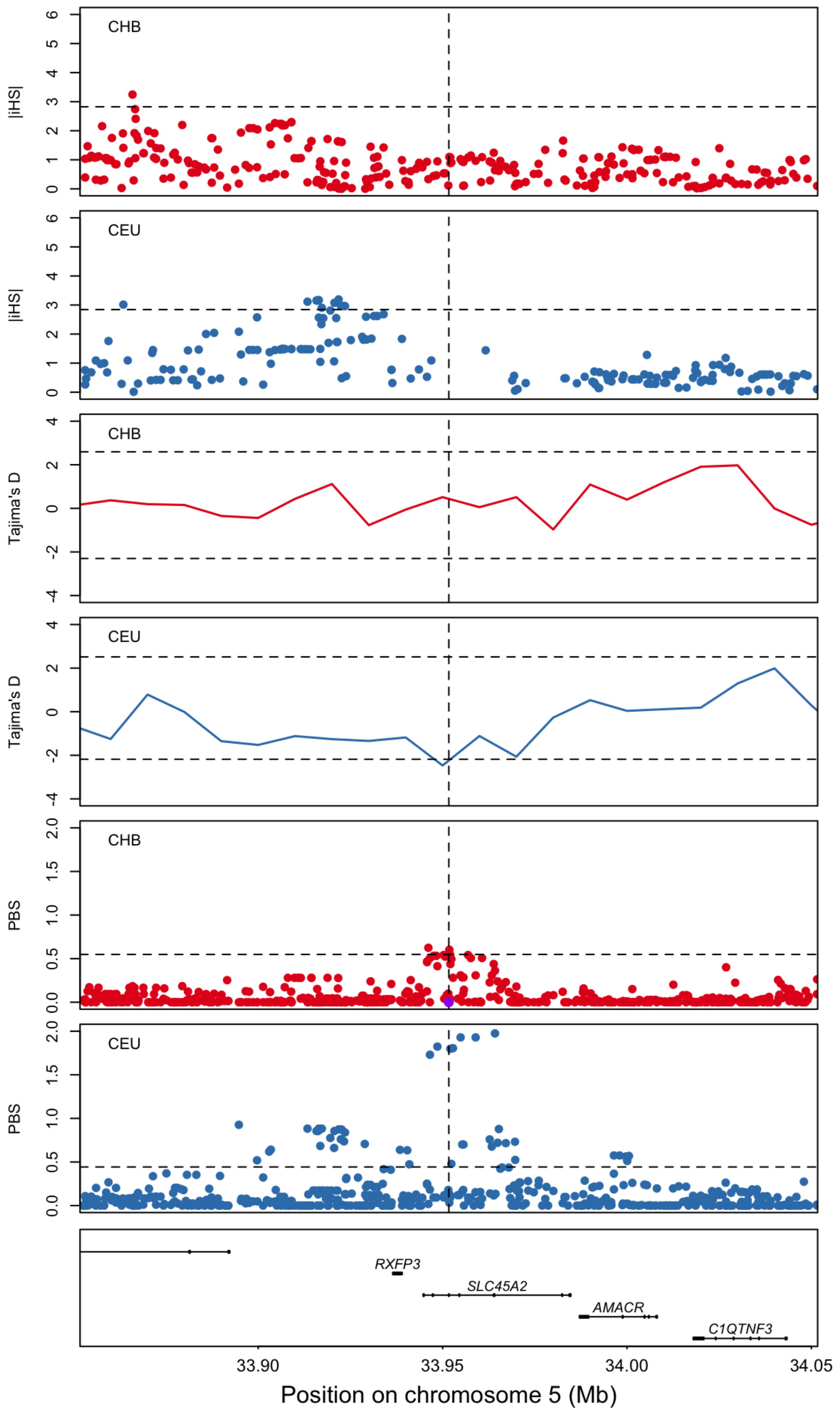

C) 6p25 – rs12203592

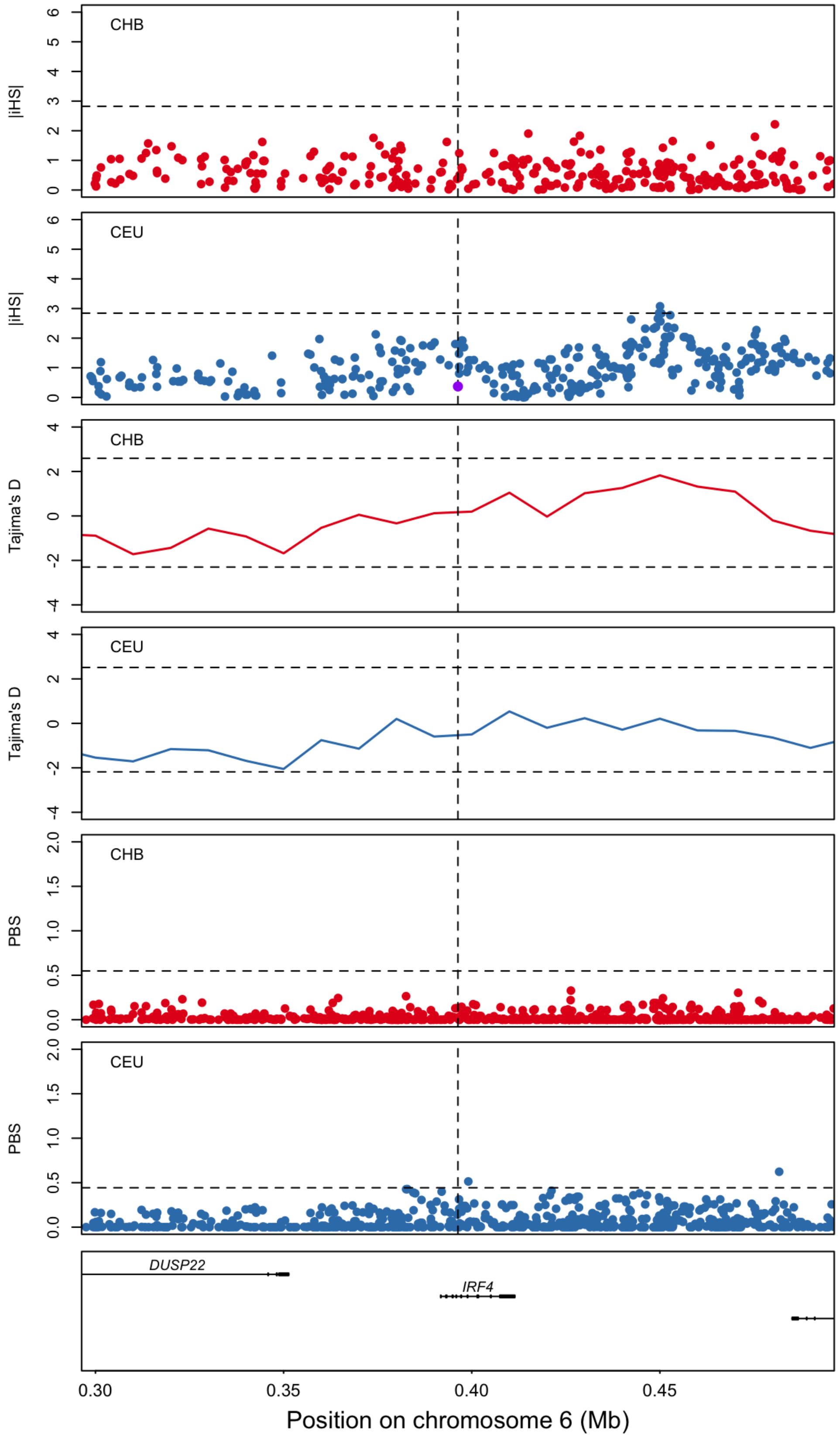

D) 9p23 – rs10809826

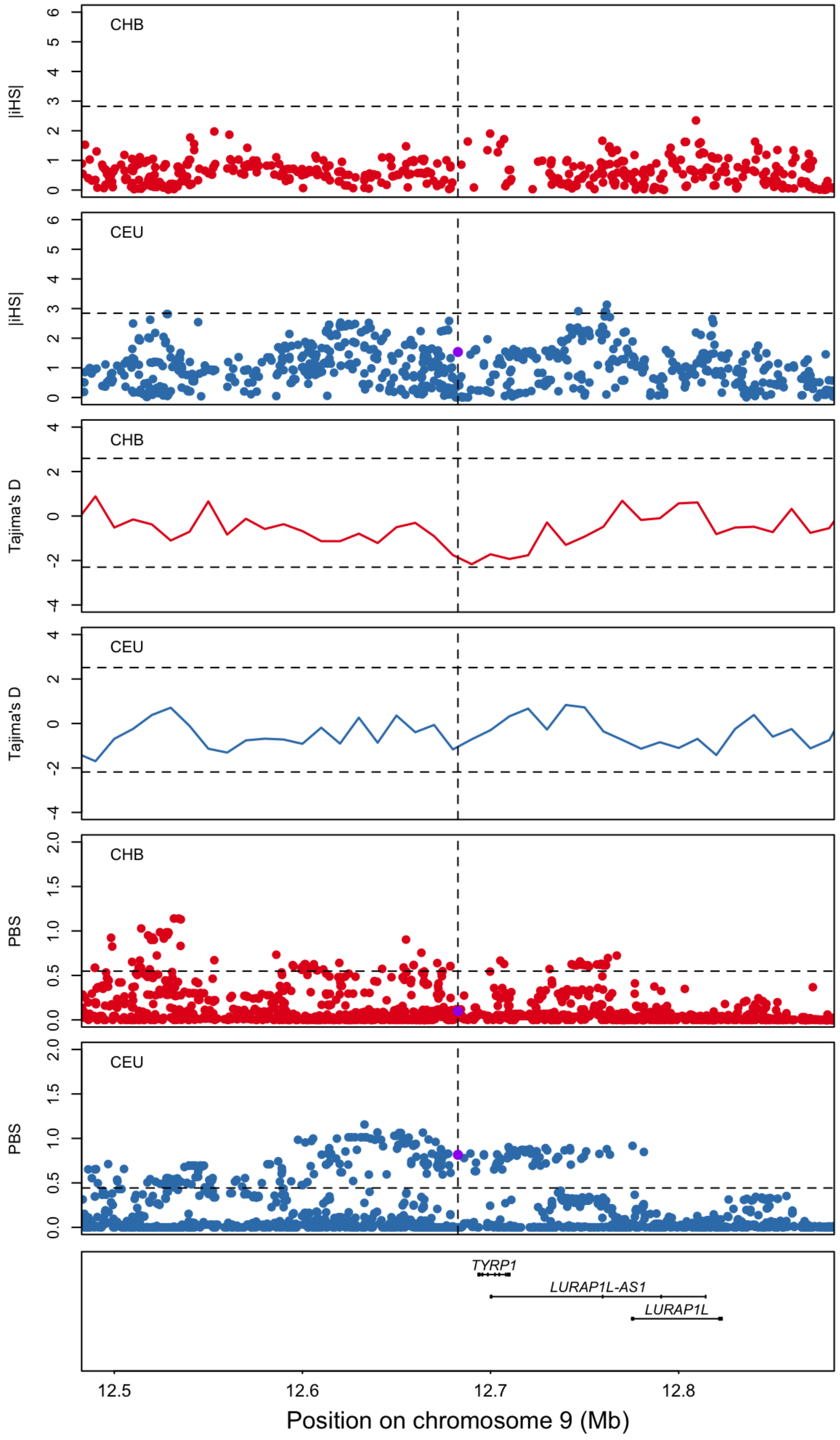

E) 10q26 – rs11198112

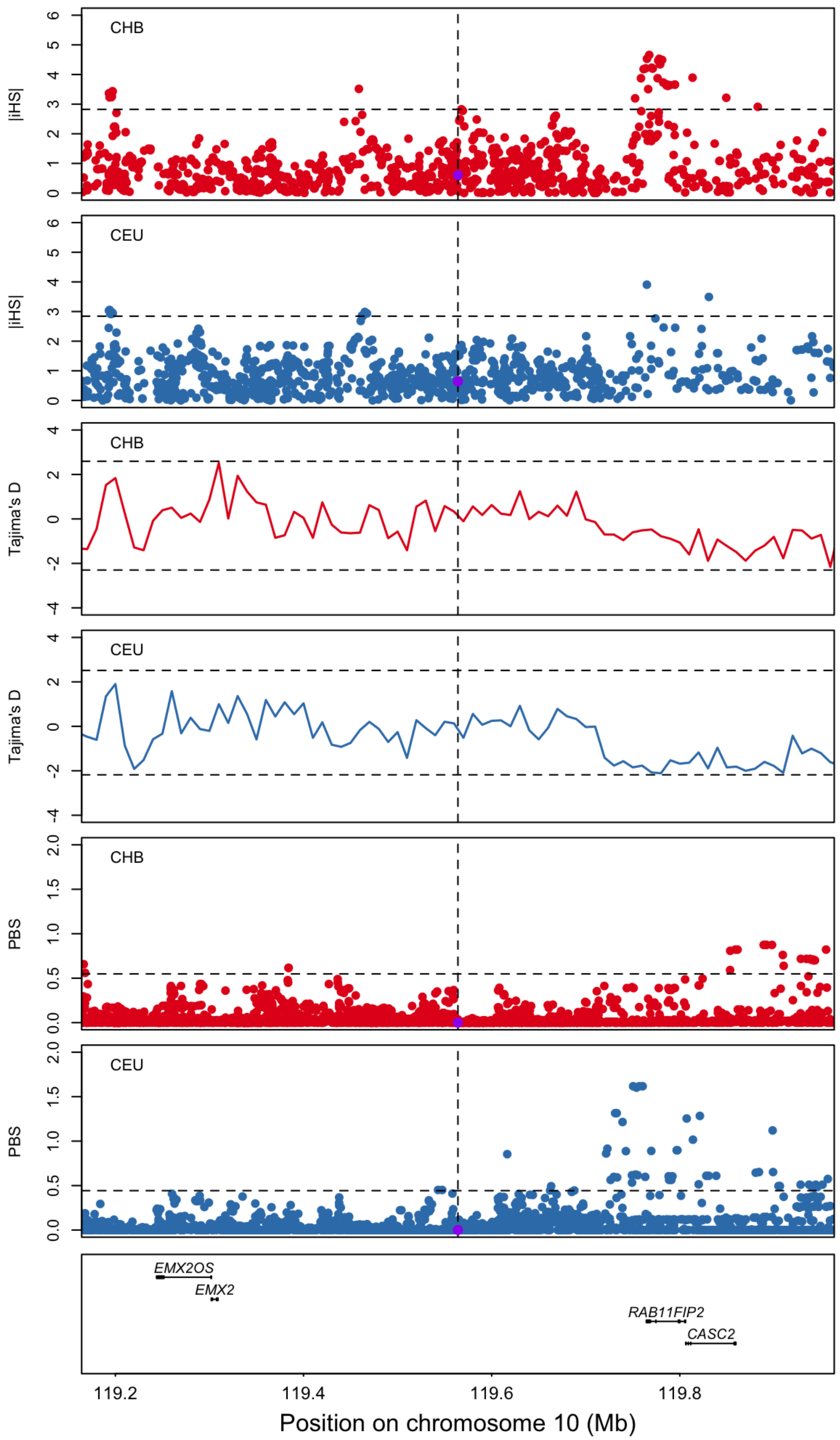

F) 11q14 – rs1042602

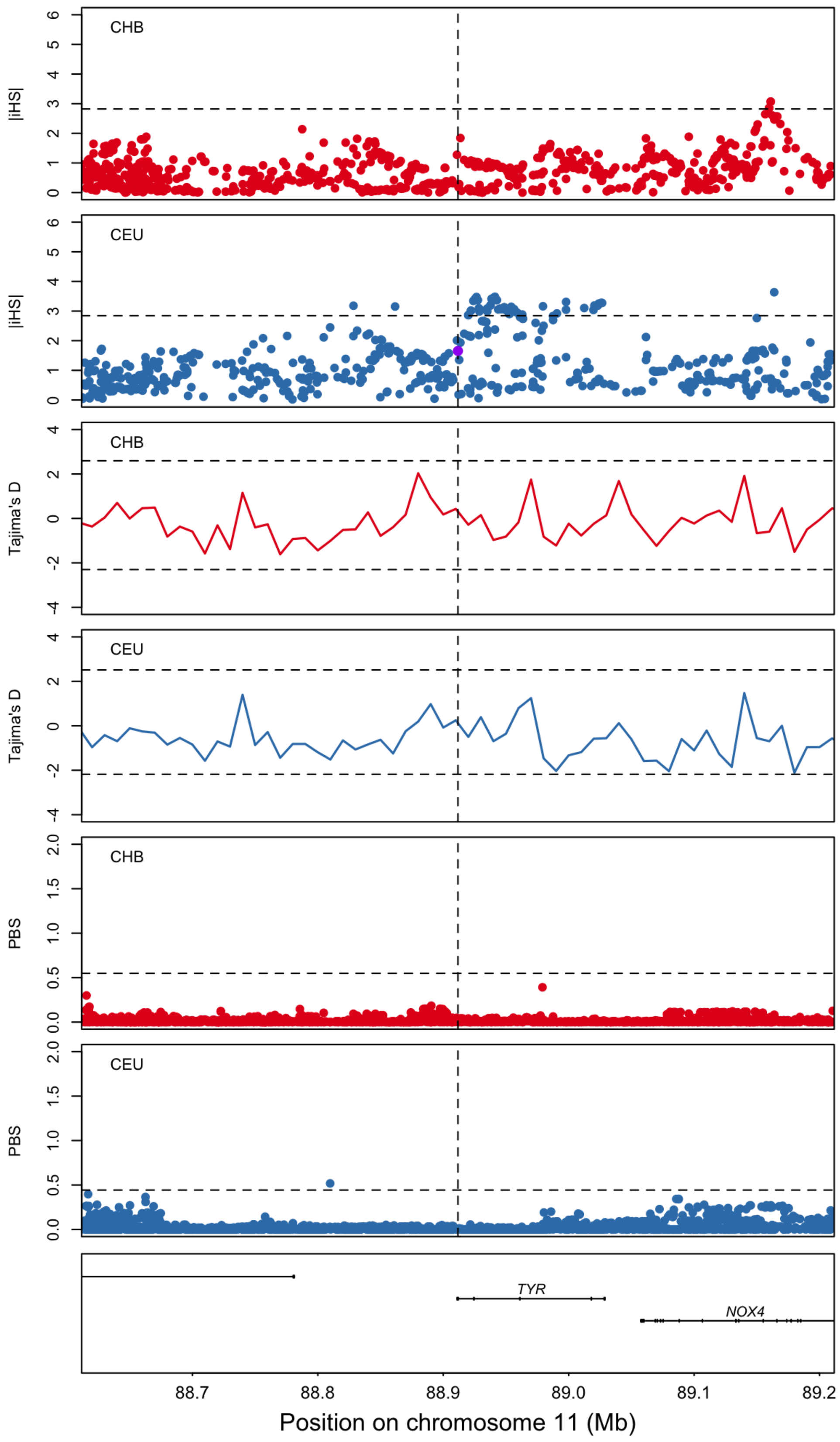

G) 11q14 – rs1126809

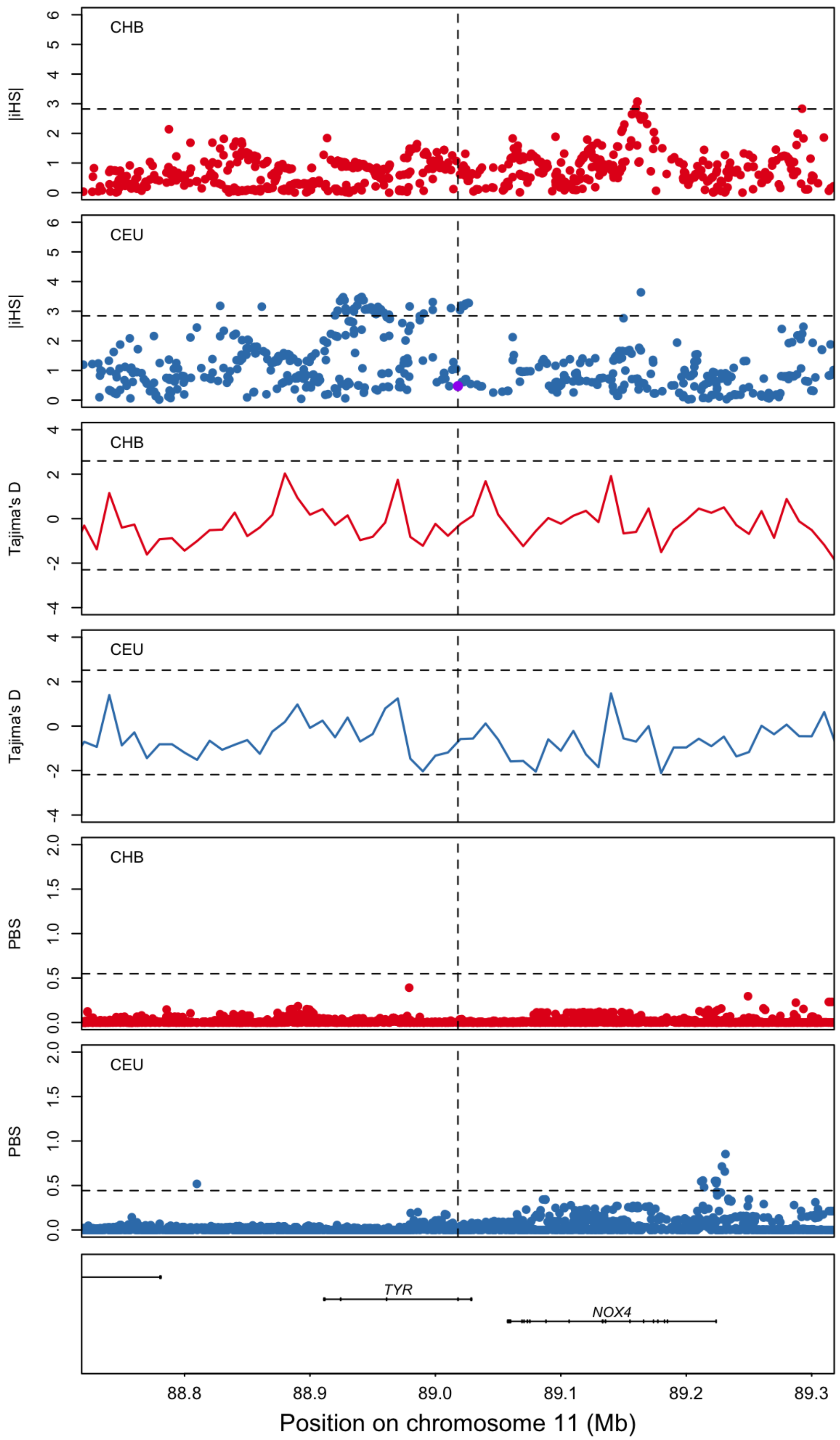

H) 11q14 – rs7118677

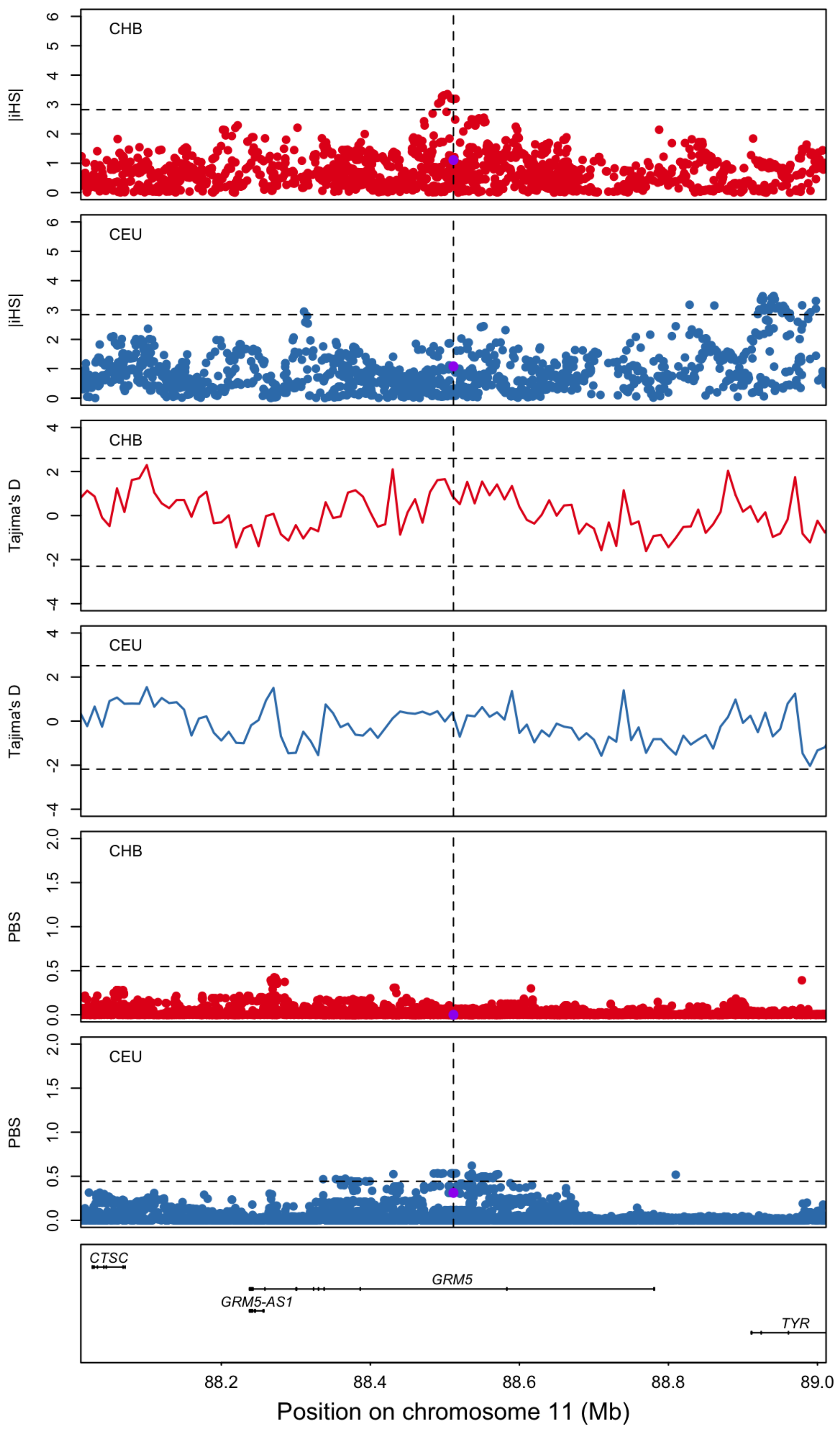

I) 15q13 – rs1800404

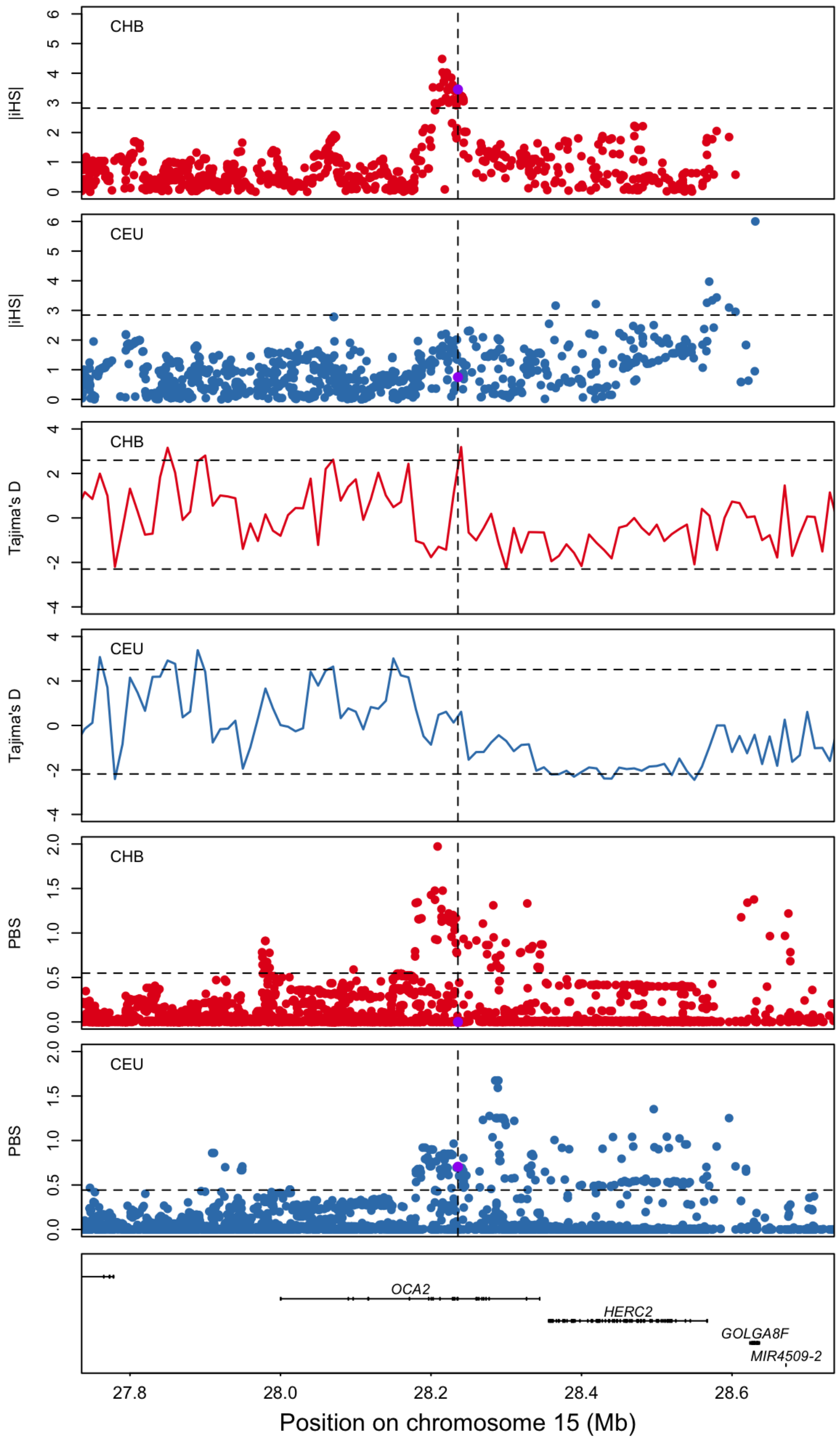

J) 15q13 – rs1800407

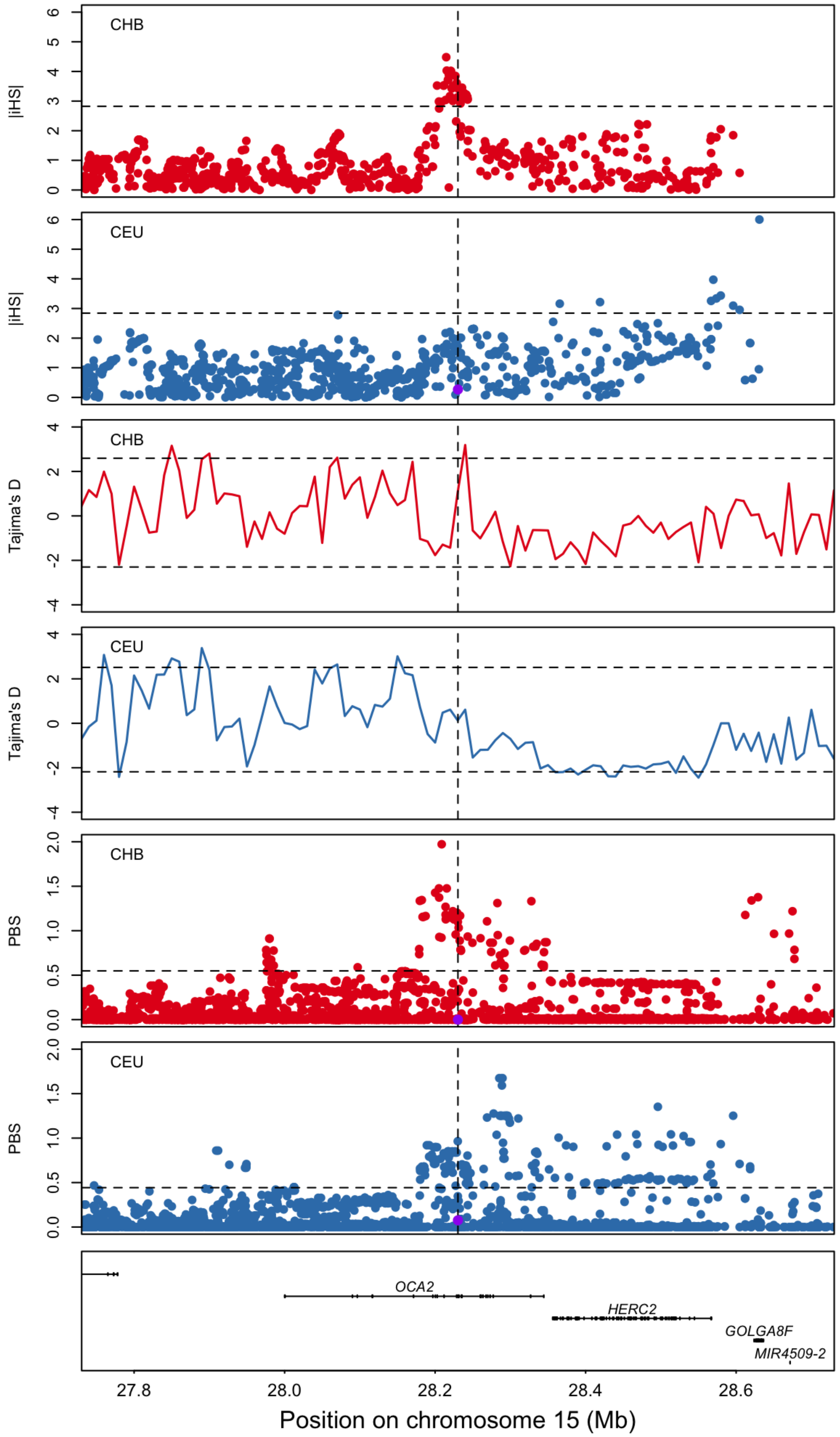

K) 15q13 – rs4778219

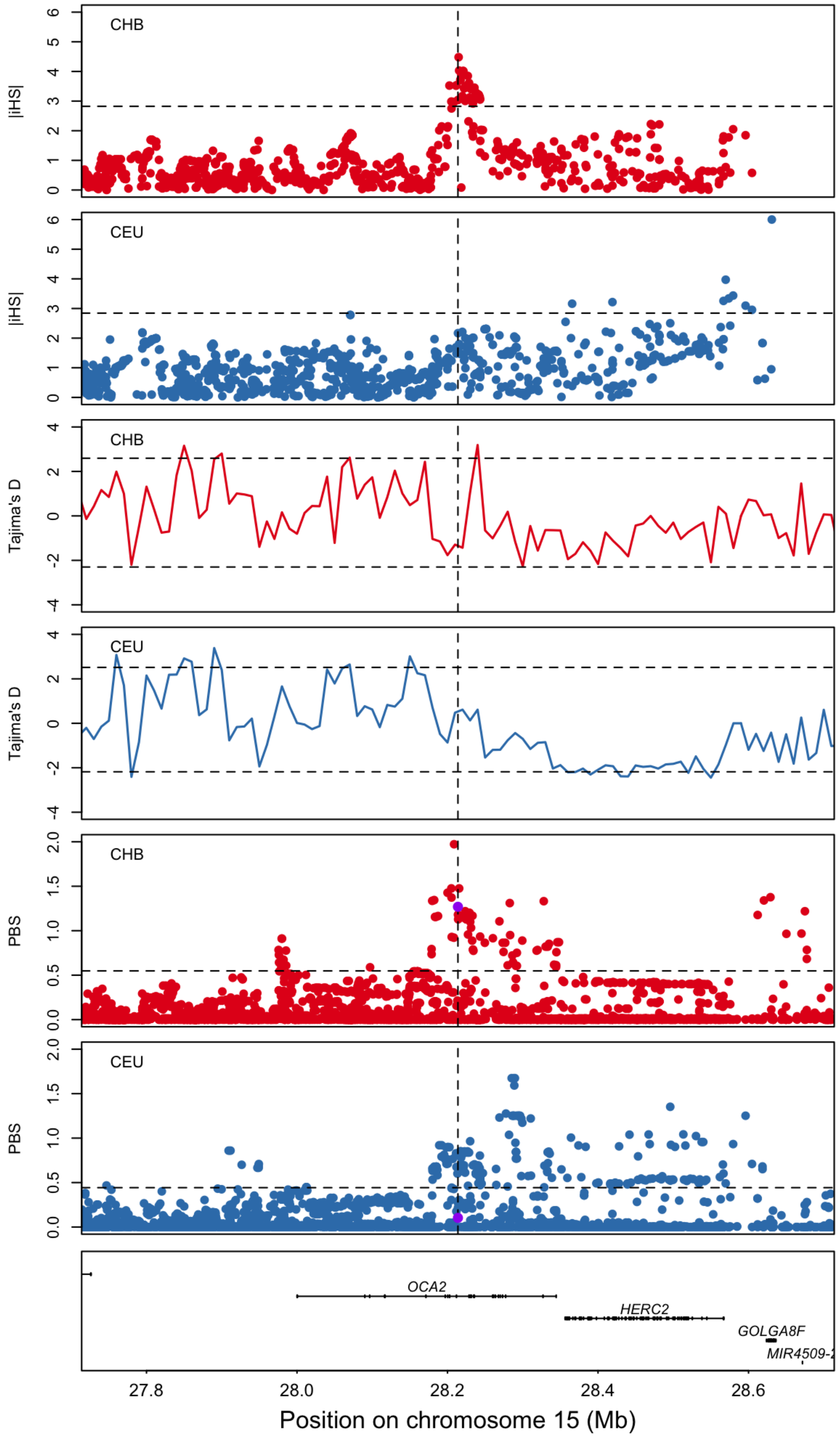

L) 15q13 – rs4778249

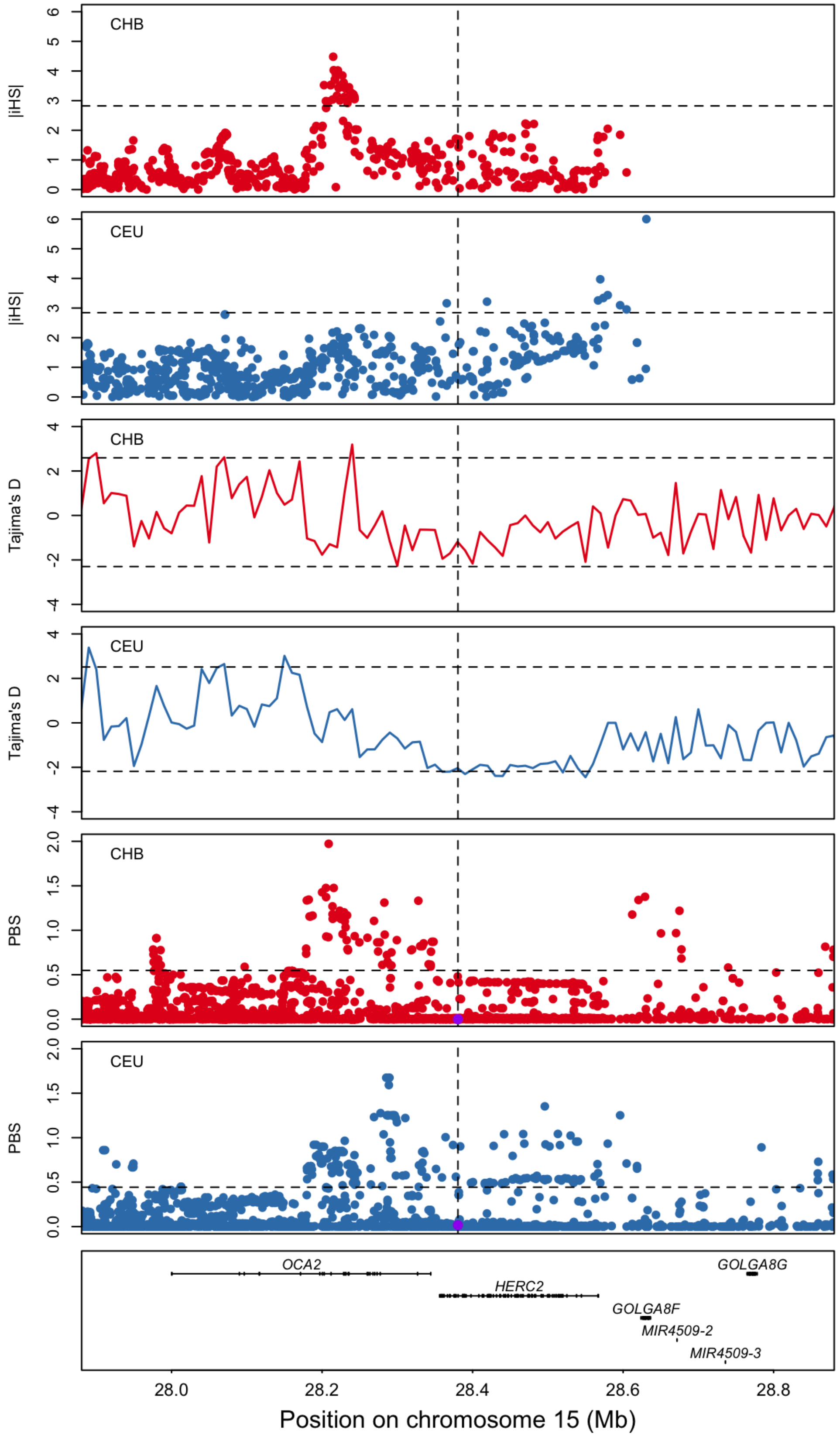

M) 15q13 – rs12913832

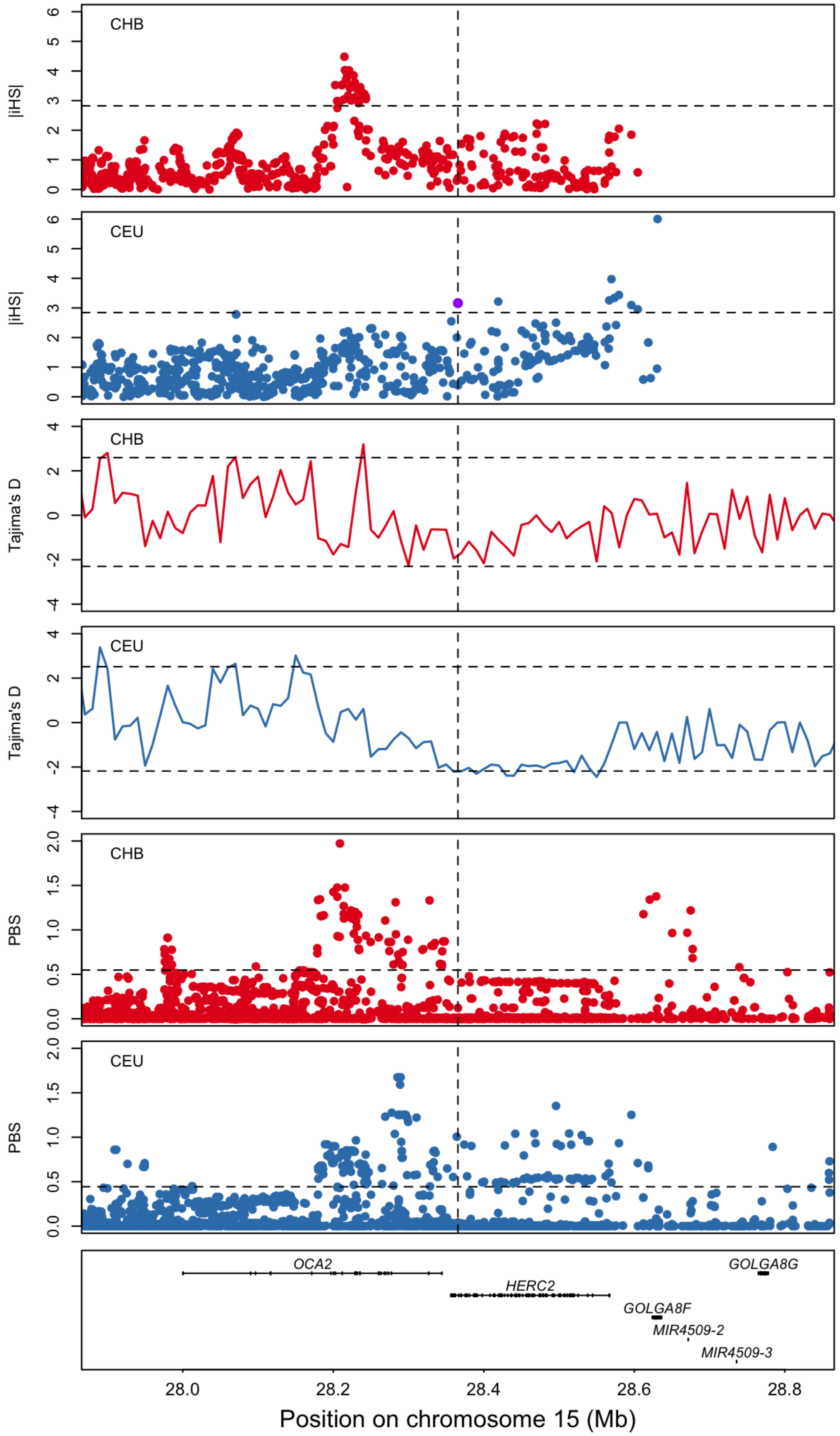

N) 15q21 – rs1426654

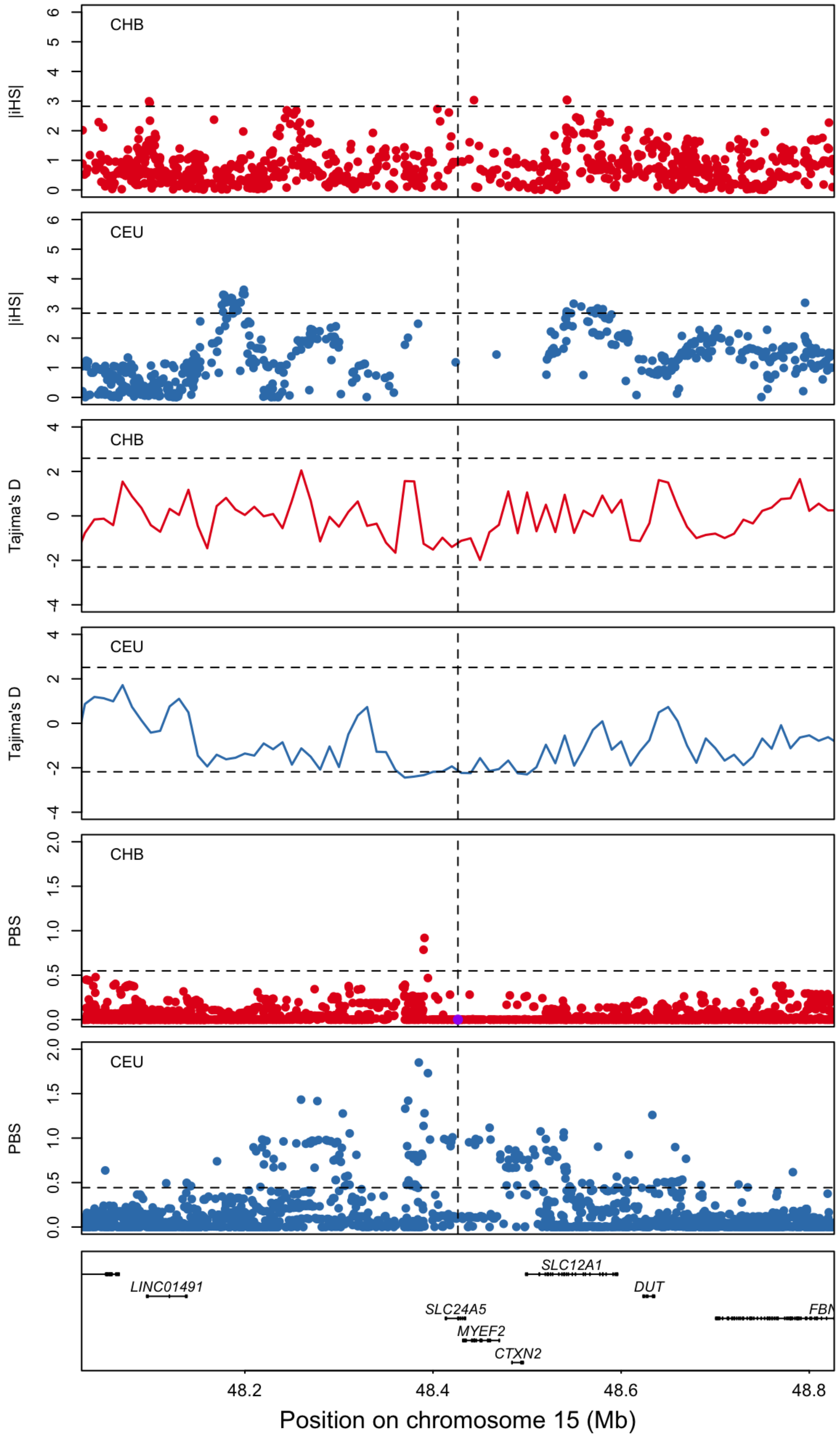

O) 16q24 - rs885479

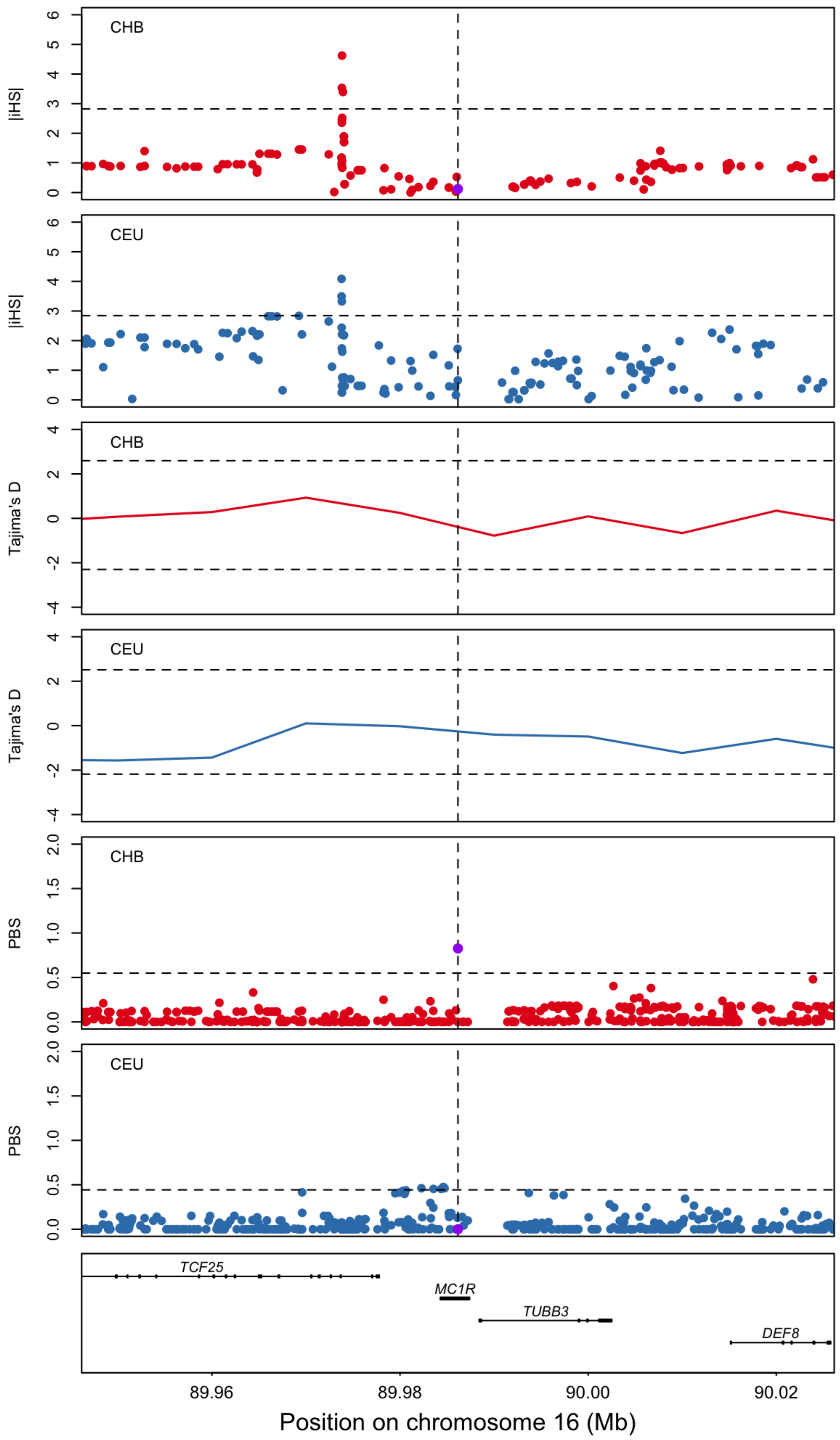

P) 19p13 – rs2240751

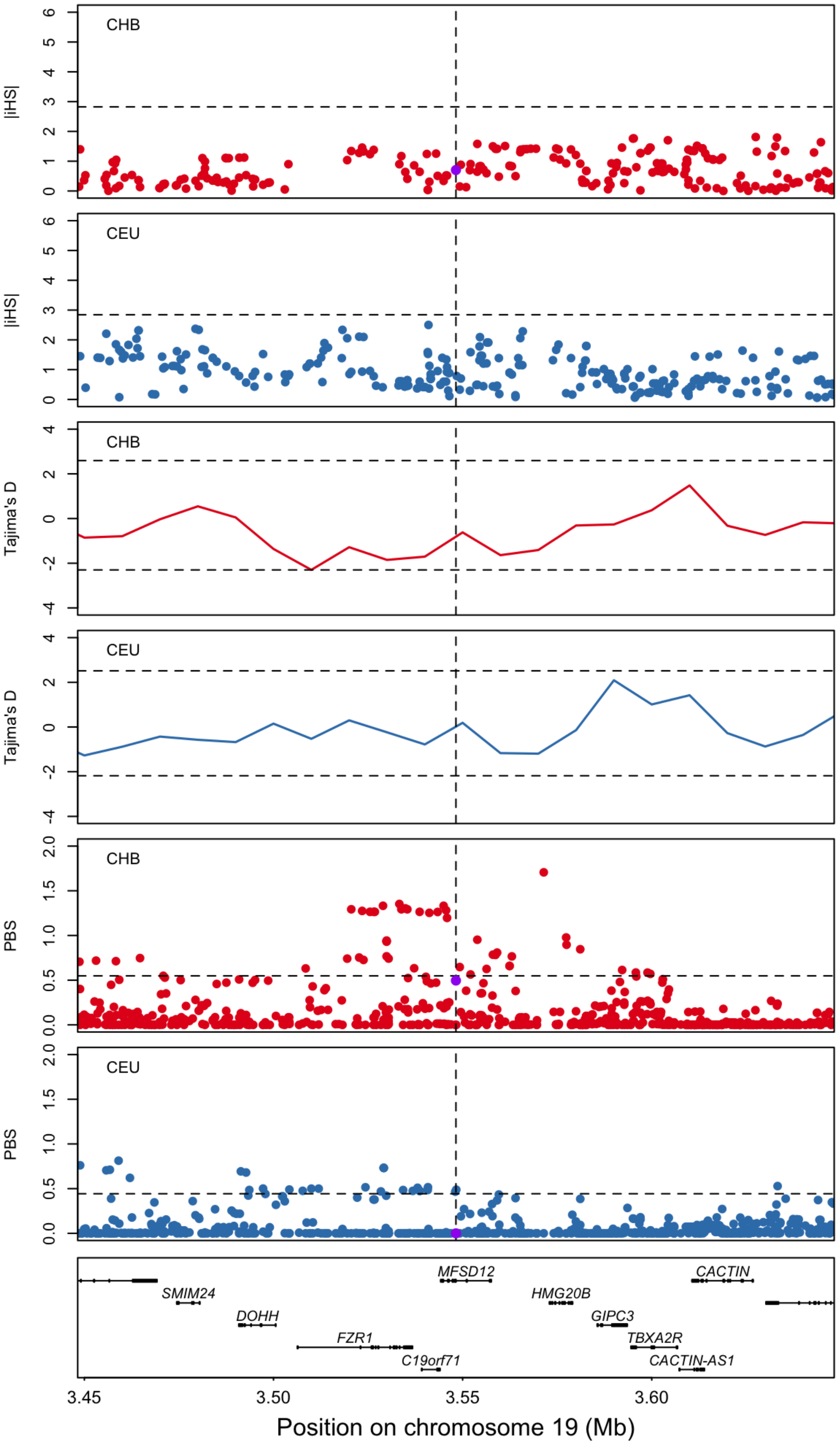

Q) 20q13 – rs17422688

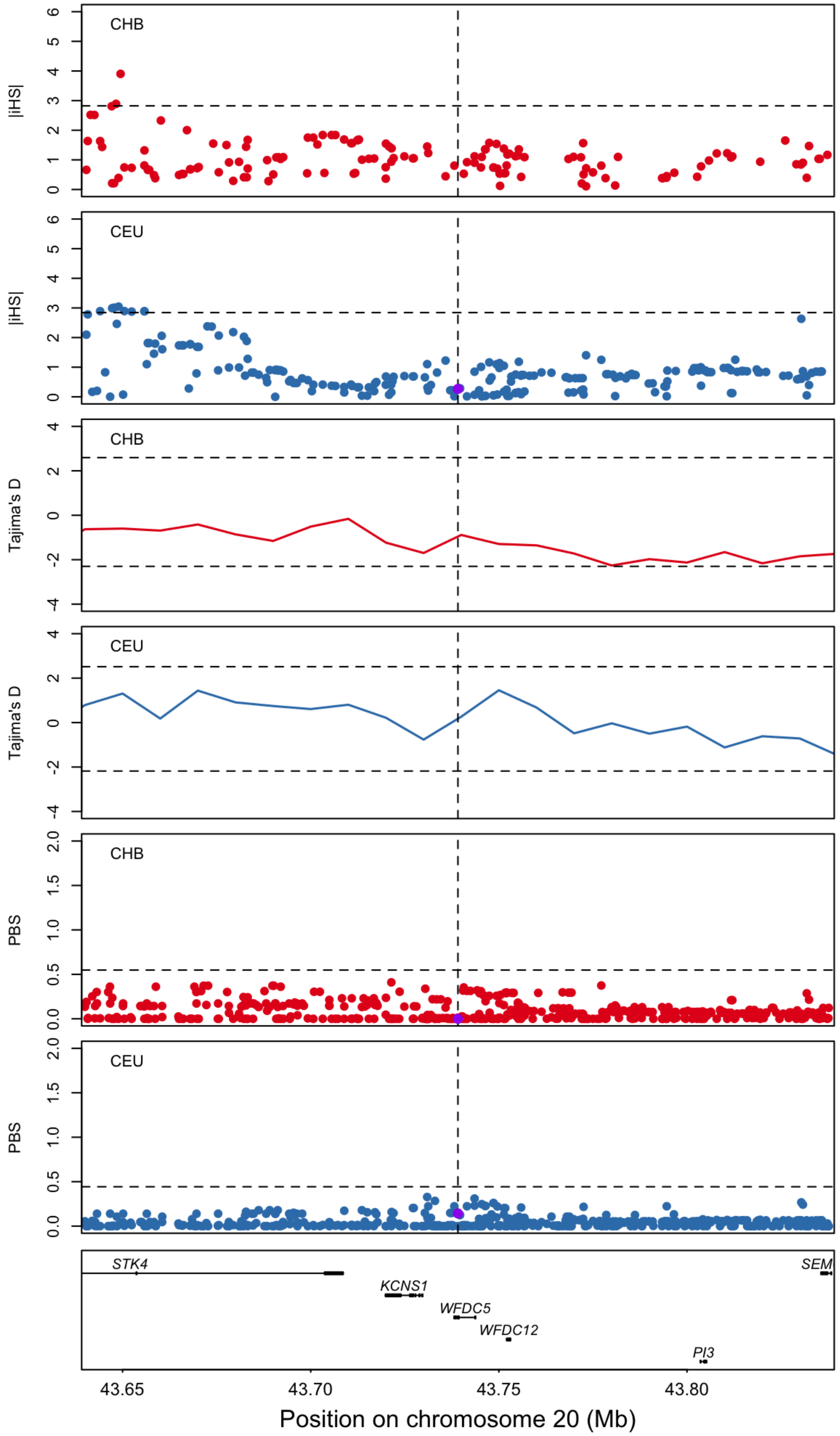

R) 22q12 – rs5756492

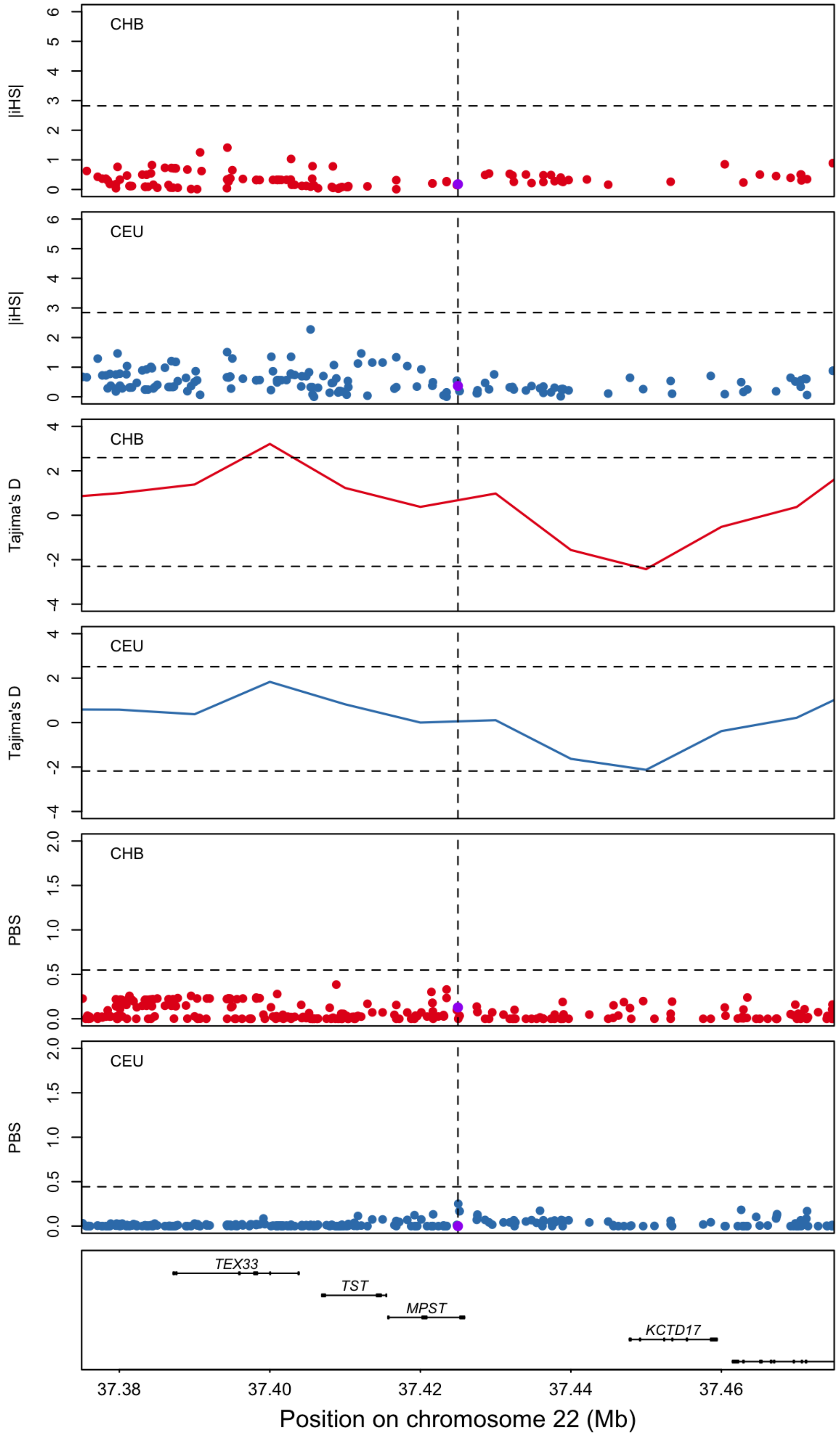

Supplementary Figure 14: RMSE plots.

Information contained within each PLS component for the start of selection time (left panel) and selection coefficient (right panel).

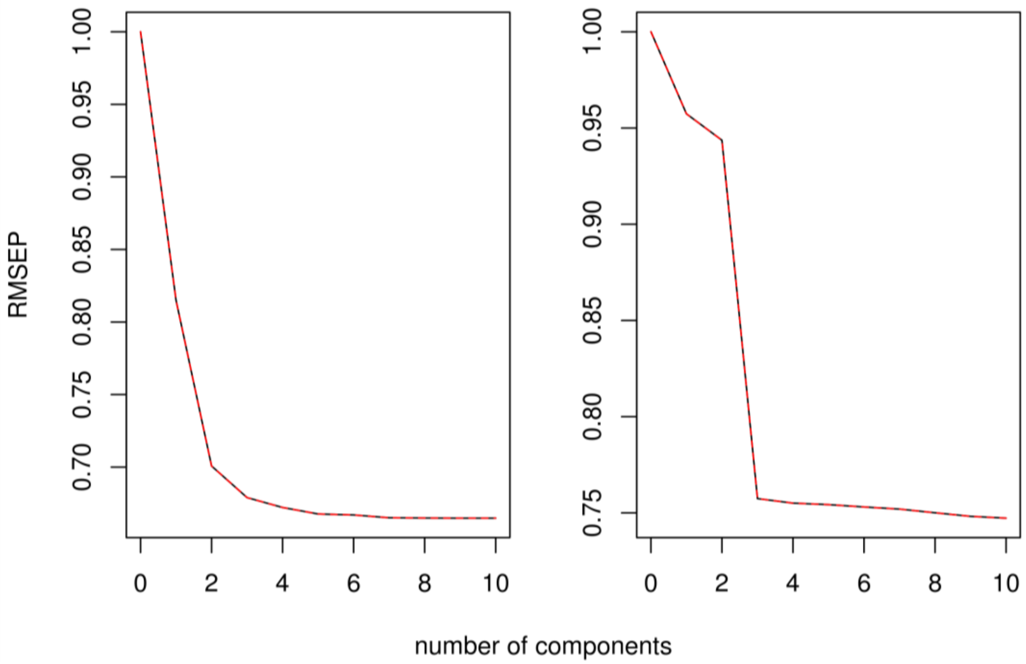

Supplementary Figure 15: Estimation of the start of selection and selection coefficient at the *MFSD12* gene region

Estimation of the start of selection (T; left panel) and selection coefficient (s; right panel) using an ABC approach. Prior and posterior distributions are represented as a red and black line, respectively. Note that the priors are not uniformly distributed as we only used simulations were the selected allele was still present at the end of the simulation. The joint posterior probability is presented in Supplementary Figure 15. As mentioned in the main text, the estimated credible intervals for these parameters are large so median point estimates should be taken with caution.

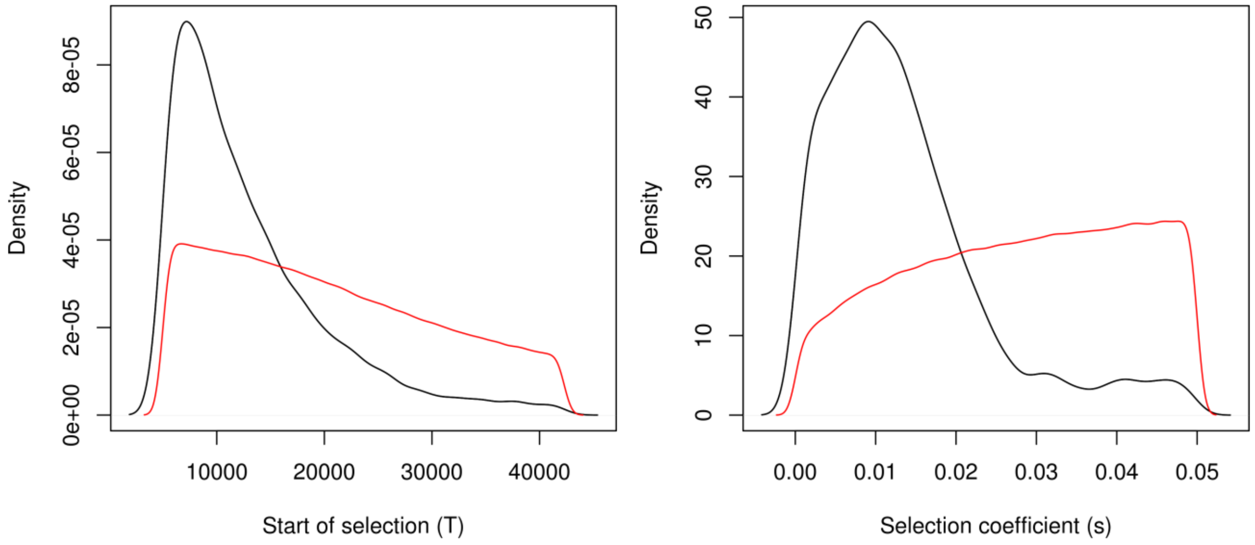

The estimated Predictive Error using the median point estimate based on a cross-validation sample of 100. The estimates were insensitive to difference tolerance rates.

Acceptance rate	Selection Time (T)	Selection coefficient (s)
0.001	0.33	0.61
0.005	0.34	0.62
0.01	0.35	0.62
0.05	0.46	0.65

Supplementary Figure 16: Joint estimation start of the start of selection and selection coefficient at the *MFSD12* gene region

Joint inference of starting time of selection (T) and selection coefficient (s) using an ABC approach. The white, yellow, and red colors mark areas of high, moderate, and low joint density respectively. The black cross indicates the joint maximum a posteriori (MAP) at s=0.139 and T = 8,508 years ago.

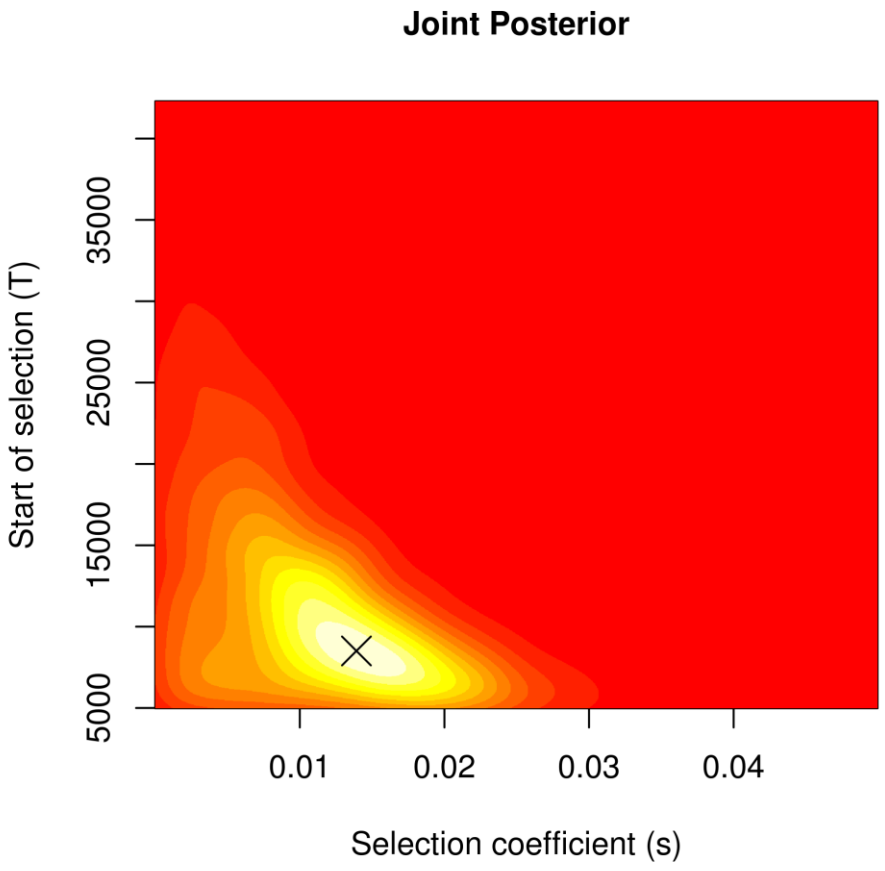

Supplementary Figure 17: Histogram of Melanin Index measurement variability

Measurements across the two arms were compared for each individual to assess variability of the Melanin Index measurement. The absolute difference between the two measurements was taken as the variability for an individual. This variability values are plotted in the histogram below, with the median shown as a purple vertical line and the quantiles as black lines.

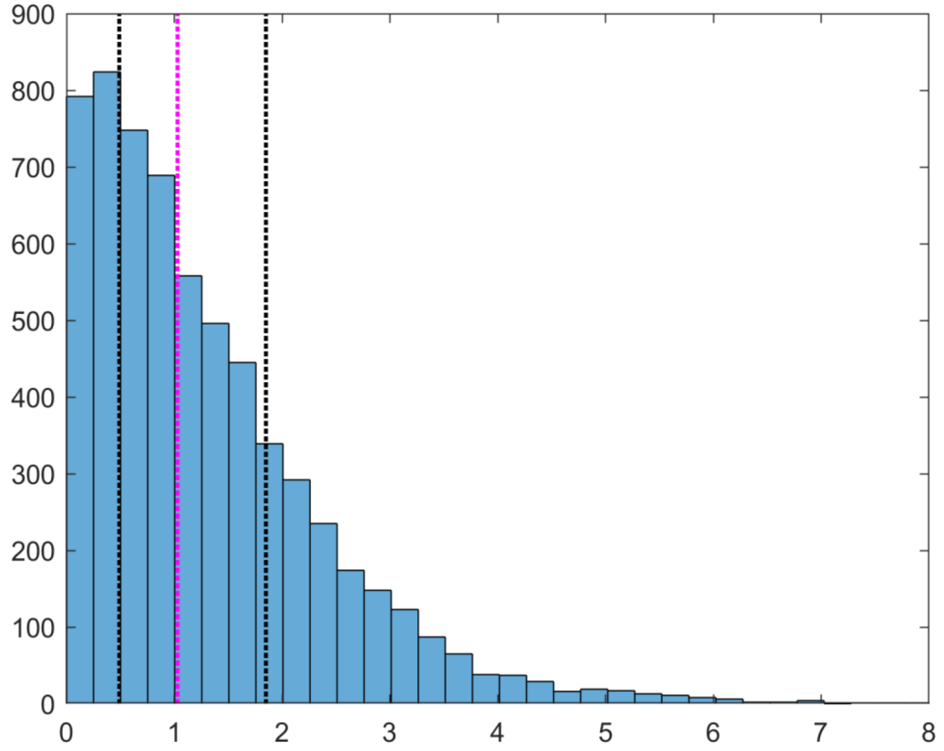

Supplementary Figure 18: Conversion of Melanin Index into approximate skin color

The DermaSpectrometer DSMEII reflectometer (Cortex Technology) uses tristimulus colorimetry to measure RGB (red, green, blue) color values and output Melanin Index (MI). The formula used for measuring MI is:

However, the measured RGB values are processed through an internal calibration step in the instrument, and hence the output RGB values for a certain MI level do not match RGB values obtainable through standard color measurement methods (such as a digital camera), and therefore doesn't match the average perceived skin color at that given MI value either (the output RGB values appear much darker, as seen in the figure below). This discrepancy was observed by simultaneously obtaining MI and RGB values from the reflectometer on a set of 100 volunteers of varying skin tone. It is most prominent at lower MI levels (e.g. 20-30), which correspond to fairer West/North European skin colors.

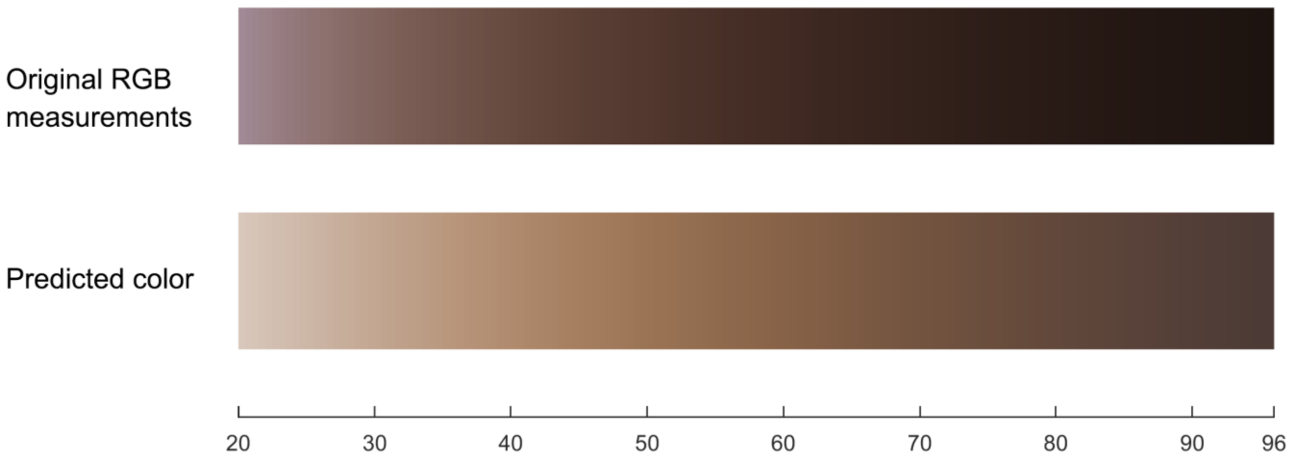

In Shriver and Parra (72) the Photovolt ColorWalk (another tristimulus colorimeter) was used to measure CIE Lab values simultaneously with MI values obtained from the Cortex DermaSpectrometer. The relationship between MI and L, a, b values as deduced from the article was used to predict Lab color values for the range of MI values observed in the whole CANDELA data. The CIE Lab values were then converted to RGB values to get the colors for plotting in Figure 1A. The following Supplementary Equations were used:

$$L = 110.1 \times e^{-0.0151 \times \mathrm{MI}} \qquad (2)$$

$$z = (\mathrm{L} - 50)/40 \qquad (3)$$

$$\mathrm{a} = 7.5689 - 0.982 \times z + 3.721 \times \cos(\pi z) \qquad (4)$$

$$\mathrm{b} = 12 + 5.5565 \times z + 10.5941 \times \cos(\pi z) \qquad (5).$$

The range of predicted Lab colors were also compared with the ranges given in (73, 74) to verify similarity in perceived color.

The range of MI values in the whole CANDELA dataset is 20-96, while after QC of individuals for GWAS (includes removing individuals with >20% African ancestry) the range shrinks to 20-65 (as shown in Figure 1A).

Supplementary Notes

Supplementary Note 1: Coalescent simulations for the Out-of-Africa model

The following msms command line was used to generate coalescent simulations of the Out-of-Africa model described in Jouganous et al. (75), that was employed in our ABC framework. For the mutation rate and the generation time we used $\mu = 1.44 \times 10^{-8}$ and $T_g = 29\ year$, respectively. As noted in the Methods section, we assumed a uniform distribution U[0 - 0.05] for the selection coefficient (`selection_coefficient`) and a uniform distribution U[5,000 – 42,229 years ago (ya)] for the starting time of selection (`selection_time`). Note that we used an additive model for the beneficial mutation, as denoted by the product of 2 for the homozygous genotype.

msms command:

```
ms -N 10000 620 1 -I 3 216 198 206 -t 288 -r 300 500000 -n 1 2.3721 -g 2
81.95 -g 3 121.96 -em 0.0 1 2 0.44 -em 0.0 1 3 0.192 -em 0.0 2 3 1.676 -eg
0.03646552 2 0.0 -eg 0.03646552 3 0.0 -ej 0.03646552 3 2 -en 0.03646552 2
0.3104 -em 0.03646552 1 2 6.32 -ej 0.1077586 2 1 -en 0.2689655 1 1.1273 -
oTrace -Sp 0.5 -SI selection_time 3 0 0.01 0.01 -Sc 0 3
selection_coefficient*2 selection_coefficient 0
```

REFERENCES

1. Beleza S, Johnson NA, Candille SI, Absher DM, Coram MA, Lopes J, et al. Genetic architecture of skin and eye color in an African-European admixed population. PLoS genetics. 2013;9(3):e1003372.
2. Adhikari K, Fuentes-Guajardo M, Quinto-Sanchez M, Mendoza-Revilla J, Camilo Chacon-Duque J, Acuna-Alonzo V, et al. A genome-wide association scan implicates DCHS2, RUNX2, GLI3, PAX1 and EDAR in human facial variation. Nature communications. 2016;7:11616.
3. Adhikari K, Reales G, Smith AJ, Konka E, Palmen J, Quinto-Sanchez M, et al. A genome-wide association study identifies multiple loci for variation in human ear morphology. Nature communications. 2015;6:7500.
4. Adhikari K, Fontanil T, Cal S, Mendoza-Revilla J, Fuentes-Guajardo M, Chacon-Duque JC, et al. A genome-wide association scan in admixed Latin Americans identifies loci influencing facial and scalp hair features. Nature communications. 2016;7:10815.
5. Franke A, McGovern DP, Barrett JC, Wang K, Radford-Smith GL, Ahmad T, et al. Genome-wide meta-analysis increases to 71 the number of confirmed Crohn's disease susceptibility loci. Nature genetics. 2010;42(12):1118-25.
6. Heid IM, Jackson AU, Randall JC, Winkler TW, Qi L, Steinthorsdottir V, et al. Meta-analysis identifies 13 new loci associated with waist-hip ratio and reveals sexual dimorphism in the genetic basis of fat distribution. Nature genetics. 2010;42(11):949-60.
7. Lango Allen H, Estrada K, Lettre G, Berndt SI, Weedon MN, Rivadeneira F, et al. Hundreds of variants clustered in genomic loci and biological pathways affect human height. Nature. 2010;467(7317):832-8.
8. Loh P-R, Kichaev G, Gazal S, Schoech AP, Price AL. Mixed model association for biobank-scale data sets. bioRxiv. 2017.
9. Manning AK, Hivert MF, Scott RA, Grimsby JL, Bouatia-Naji N, Chen H, et al. A genome-wide approach accounting for body mass index identifies genetic variants influencing fasting glycemic traits and insulin resistance. Nature genetics. 2012;44(6):659-69.
10. Okbay A, Baselmans BM, De Neve JE, Turley P, Nivard MG, Fontana MA, et al. Genetic variants associated with subjective well-being, depressive symptoms, and neuroticism identified through genome-wide analyses. Nature genetics. 2016;48(6):624-33.
11. Speliotes EK, Willer CJ, Berndt SI, Monda KL, Thorleifsson G, Jackson AU, et al. Association analyses of 249,796 individuals reveal 18 new loci associated with body mass index. Nature genetics. 2010;42(11):937-48.
12. Teslovich TM, Musunuru K, Smith AV, Edmondson AC, Stylianou IM, Koseki M, et al. Biological, clinical and population relevance of 95 loci for blood lipids. Nature. 2010;466(7307):707-13.
13. Wang J, Carvajal-Carmona LG, Chu JH, Zauber AG, Collaborators APCT, Kubo M, et al. Germline variants and advanced colorectal adenomas: adenoma prevention with celecoxib trial genome-wide association study. Clinical cancer research : an official journal of the American Association for Cancer Research. 2013;19(23):6430-7.
14. Wen CC, Yee SW, Liang X, Hoffmann TJ, Kvale MN, Banda Y, et al. Genome-wide association study identifies ABCG2 (BCRP) as an allopurinol transporter and a determinant of drug response. Clinical pharmacology and therapeutics. 2015;97(5):518-25.
15. Wheeler HE, Gamazon ER, Frisina RD, Perez-Cervantes C, El Charif O, Mapes B, et al. Variants in WFS1 and Other Mendelian Deafness Genes Are Associated with Cisplatin-Associated Ototoxicity. Clinical cancer research : an official journal of the American Association for Cancer Research. 2017;23(13):3325-33.
16. Willer CJ, Schmidt EM, Sengupta S, Peloso GM, Gustafsson S, Kanoni S, et al. Discovery and refinement of loci associated with lipid levels. Nature genetics. 2013;45(11):1274-83.
17. Zhou X, Stephens M. Efficient multivariate linear mixed model algorithms for genome-wide association studies. Nature methods. 2014;11(4):407-9.
18. Stephens M. A unified framework for association analysis with multiple related phenotypes. PloS one. 2013;8(7):e65245.

19. Hastings GA, Coleman TA, Haudenschild CC, Stefansson S, Smith EP, Barthlow R, et al. Neuroserpin, a brain-associated inhibitor of tissue plasminogen activator is localized primarily in neurons. Implications for the regulation of motor learning and neuronal survival. The Journal of biological chemistry. 1997;272(52):33062-7.
20. Scherfer C, Tang H, Kambris Z, Lhocine N, Hashimoto C, Lemaitre B. Drosophila Serpin-28D regulates hemolymph phenoloxidase activity and adult pigmentation. Developmental biology. 2008;323(2):189-96.
21. Irving JA, Pike RN, Lesk AM, Whisstock JC. Phylogeny of the serpin superfamily: implications of patterns of amino acid conservation for structure and function. Genome research. 2000;10(12):1845-64.
22. Steele FR, Chader GJ, Johnson LV, Tombran-Tink J. Pigment epithelium-derived factor: neurotrophic activity and identification as a member of the serine protease inhibitor gene family. Proceedings of the National Academy of Sciences of the United States of America. 1993;90(4):1526-30.
23. Chacon-Duque JC, Adhikari K, Fuentes-Guajardo M, Mendoza-Revilla J, Acuna-Alonzo V, Barquera Lozano R, et al. Latin Americans show wide-spread Converso ancestry and the imprint of local Native ancestry on physical appearance. bioRxiv. 2018.
24. Genomes Project C, Auton A, Brooks LD, Durbin RM, Garrison EP, Kang HM, et al. A global reference for human genetic variation. Nature. 2015;526(7571):68-74.
25. Schlebusch CM, Skoglund P, Sjodin P, Gattepaille LM, Hernandez D, Jay F, et al. Genomic variation in seven Khoe-San groups reveals adaptation and complex African history. Science. 2012;338(6105):374-9.
26. Eichstaedt CA, Antao T, Pagani L, Cardona A, Kivisild T, Mormina M. The Andean adaptive toolkit to counteract high altitude maladaptation: genome-wide and phenotypic analysis of the Collas. PloS one. 2014;9(3):e93314.
27. Mallick S, Li H, Lipson M, Mathieson I, Gymrek M, Racimo F, et al. The Simons Genome Diversity Project: 300 genomes from 142 diverse populations. Nature. 2016;538(7624):201-6.
28. Morseburg A, Pagani L, Ricaut FX, Yngvadottir B, Harney E, Castillo C, et al. Multi-layered population structure in Island Southeast Asians. European journal of human genetics : EJHG. 2016;24(11):1605-11.
29. Cardona A, Pagani L, Antao T, Lawson DJ, Eichstaedt CA, Yngvadottir B, et al. Genome-wide analysis of cold adaptation in indigenous Siberian populations. PloS one. 2014;9(5):e98076.
30. Stacey SN, Gudbjartsson DF, Sulem P, Bergthorsson JT, Kumar R, Thorleifsson G, et al. Common variants on 1p36 and 1q42 are associated with cutaneous basal cell carcinoma but not with melanoma or pigmentation traits. Nature genetics. 2008;40(11):1313-8.
31. Hernandez-Pacheco N, Flores C, Alonso S, Eng C, Mak AC, Hunstman S, et al. Identification of a novel locus associated with skin colour in African-admixed populations. Scientific reports. 2017;7:44548.
32. Hou S, Du L, Lei B, Pang CP, Zhang M, Zhuang W, et al. Genome-wide association analysis of Vogt-Koyanagi-Harada syndrome identifies two new susceptibility loci at 1p31.2 and 10q21.3. Nature genetics. 2014;46(9):1007-11.
33. Martin AR, Lin M, Granka JM, Myrick JW, Liu X, Sockell A, et al. An Unexpectedly Complex Architecture for Skin Pigmentation in Africans. Cell. 2017;171(6):1340-53 e14.
34. Zhang M, Song F, Liang L, Nan H, Zhang J, Liu H, et al. Genome-wide association studies identify several new loci associated with pigmentation traits and skin cancer risk in European Americans. Human molecular genetics. 2013;22(14):2948-59.
35. Edwards M, Cha D, Krithika S, Johnson M, Cook G, Parra EJ. Iris pigmentation as a quantitative trait: variation in populations of European, East Asian and South Asian ancestry and association with candidate gene polymorphisms. Pigment cell & melanoma research. 2016;29(2):141-62.
36. Liu F, Visser M, Duffy DL, Hysi PG, Jacobs LC, Lao O, et al. Genetics of skin color variation in Europeans: genome-wide association studies with functional follow-up. Human genetics. 2015;134(8):823-35.
37. Law MH, Medland SE, Zhu G, Yazar S, Vinuela A, Wallace L, et al. Genome-Wide Association Shows that Pigmentation Genes Play a Role in Skin Aging. The Journal of investigative dermatology. 2017;137(9):1887-94.
38. Jacobs LC, Wollstein A, Lao O, Hofman A, Klaver CC, Uitterlinden AG, et al. Comprehensive candidate gene study highlights UGT1A and BNC2 as new genes determining continuous skin color variation in Europeans. Human genetics. 2013;132(2):147-58.

39. Lloyd-Jones LR, Robinson MR, Moser G, Zeng J, Beleza S, Barsh GS, et al. Inference on the Genetic Basis of Eye and Skin Color in an Admixed Population via Bayesian Linear Mixed Models. Genetics. 2017;206(2):1113-26.
40. Eriksson N, Macpherson JM, Tung JY, Hon LS, Naughton B, Saxonov S, et al. Web-based, participant-driven studies yield novel genetic associations for common traits. PLoS genetics. 2010;6(6):e1000993.
41. Lin BD, Mbarek H, Willemsen G, Dolan CV, Fedko IO, Abdellaoui A, et al. Heritability and Genome-Wide Association Studies for Hair Color in a Dutch Twin Family Based Sample. Genes. 2015;6(3):559-76.
42. Stokowski RP, Pant PV, Dadd T, Fereday A, Hinds DA, Jarman C, et al. A genomewide association study of skin pigmentation in a South Asian population. American journal of human genetics. 2007;81(6):1119-32.
43. Sturm RA. Molecular genetics of human pigmentation diversity. Human molecular genetics. 2009;18(R1):R9-17.
44. Sturm RA. GSTP1 and MC1R in melanoma susceptibility. The British journal of dermatology. 2012;166(6):1155-6.
45. Nan H, Kraft P, Hunter DJ, Han J. Genetic variants in pigmentation genes, pigmentary phenotypes, and risk of skin cancer in Caucasians. International journal of cancer. 2009;125(4):909-17.
46. Han J, Kraft P, Nan H, Guo Q, Chen C, Qureshi A, et al. A genome-wide association study identifies novel alleles associated with hair color and skin pigmentation. PLoS genetics. 2008;4(5):e1000074.
47. Graf J, Hodgson R, van Daal A. Single nucleotide polymorphisms in the MATP gene are associated with normal human pigmentation variation. Human mutation. 2005;25(3):278-84.
48. Graf J, Voisey J, Hughes I, van Daal A. Promoter polymorphisms in the MATP (SLC45A2) gene are associated with normal human skin color variation. Human mutation. 2007;28(7):710-7.
49. Candille SI, Absher DM, Beleza S, Bauchet M, McEvoy B, Garrison NA, et al. Genome-wide association studies of quantitatively measured skin, hair, and eye pigmentation in four European populations. PloS one. 2012;7(10):e48294.
50. Jacobs LC, Hamer MA, Gunn DA, Deelen J, Lall JS, van Heemst D, et al. A Genome-Wide Association Study Identifies the Skin Color Genes IRF4, MC1R, ASIP, and BNC2 Influencing Facial Pigmented Spots. The Journal of investigative dermatology. 2015;135(7):1735-42.
51. Sulem P, Gudbjartsson DF, Stacey SN, Helgason A, Rafnar T, Magnusson KP, et al. Genetic determinants of hair, eye and skin pigmentation in Europeans. Nature genetics. 2007;39(12):1443-52.
52. Yang Z, Zhong H, Chen J, Zhang X, Zhang H, Luo X, et al. A Genetic Mechanism for Convergent Skin Lightening during Recent Human Evolution. Molecular biology and evolution. 2016;33(5):1177-87.
53. Laville V, Clerc SL, Ezzedine K, Jdid R, Taing L, Labib T, et al. A genome-wide association study in Caucasian women suggests the involvement of HLA genes in the severity of facial solar lentigines. Pigment cell & melanoma research. 2016;29(5):550-8.
54. Kenny EE, Timpson NJ, Sikora M, Yee M-C, Moreno-Estrada A, Eng C, et al. Melanesian blond hair is caused by an amino acid change in TYRP1. Science. 2012;336(6081):554-.
55. Sulem P, Gudbjartsson DF, Stacey SN, Helgason A, Rafnar T, Jakobsdottir M, et al. Two newly identified genetic determinants of pigmentation in Europeans. Nature genetics. 2008;40(7):835-7.
56. Crawford NG, Kelly DE, Hansen MEB, Beltrame MH, Fan S, Bowman SL, et al. Loci associated with skin pigmentation identified in African populations. Science. 2017;358(6365).
57. Abe Y, Tamiya G, Nakamura T, Hozumi Y, Suzuki T. Association of melanogenesis genes with skin color variation among Japanese females. Journal of dermatological science. 2013;69(2):167-72.
58. Eaton K, Edwards M, Krithika S, Cook G, Norton H, Parra EJ. Association study confirms the role of two OCA2 polymorphisms in normal skin pigmentation variation in East Asian populations. American journal of human biology : the official journal of the Human Biology Council. 2015;27(4):520-5.
59. Edwards M, Bigham A, Tan J, Li S, Gozdzik A, Ross K, et al. Association of the OCA2 polymorphism His615Arg with melanin content in east Asian populations: further evidence of convergent evolution of skin pigmentation. PLoS genetics. 2010;6(3):e1000867.
60. Rawofi L, Edwards M, Krithika S, Le P, Cha D, Yang Z, et al. Genome-wide association study of pigmentary traits (skin and iris color) in individuals of East Asian ancestry. PeerJ. 2017;5:e3951.

61. Pospiech E, Draus-Barini J, Kupiec T, Wojas-Pelc A, Branicki W. Gene-gene interactions contribute to eye colour variation in humans. Journal of human genetics. 2011;56(6):447-55.
62. Liu F, Wollstein A, Hysi PG, Ankra-Badu GA, Spector TD, Park D, et al. Digital quantification of human eye color highlights genetic association of three new loci. PLoS genetics. 2010;6(5):e1000934.
63. Sturm RA, Duffy DL. Human pigmentation genes under environmental selection. Genome biology. 2012;13(9):248.
64. Yamaguchi K, Watanabe C, Kawaguchi A, Sato T, Naka I, Shindo M, et al. Association of melanocortin 1 receptor gene (MC1R) polymorphisms with skin reflectance and freckles in Japanese. Journal of human genetics. 2012;57(11):700-8.
65. Kanetsky PA, Swoyer J, Panossian S, Holmes R, Guerry D, Rebbeck TR. A polymorphism in the agouti signaling protein gene is associated with human pigmentation. American journal of human genetics. 2002;70(3):770-5.
66. Schmid P, Fischer S, editors. Colour segmentation for the analysis of pigmented skin lesions. Image Processing and Its Applications, 1997, Sixth International Conference on; 1997: IET.
67. Mengel-From J, Wong TH, Morling N, Rees JL, Jackson IJ. Genetic determinants of hair and eye colours in the Scottish and Danish populations. BMC genetics. 2009;10:88.
68. Norton HL, Edwards M, Krithika S, Johnson M, Werren EA, Parra EJ. Quantitative assessment of skin, hair, and iris variation in a diverse sample of individuals and associated genetic variation. American journal of physical anthropology. 2016;160(4):570-81.
69. Hastie T, Stuetzle W. Principal curves. Journal of the American Statistical Association. 1989;84(406):502-16.
70. Alexander DH, Novembre J, Lange K. Fast model-based estimation of ancestry in unrelated individuals. Genome Res. 2009;19(9):1655-64.
71. Ruiz-Linares A, Adhikari K, Acuna-Alonzo V, Quinto-Sanchez M, Jaramillo C, Arias W, et al. Admixture in Latin America: geographic structure, phenotypic diversity and self-perception of ancestry based on 7,342 individuals. PLoS genetics. 2014;10(9):e1004572.
72. Shriver MD, Parra EJ. Comparison of narrow-band reflectance spectroscopy and tristimulus colorimetry for measurements of skin and hair color in persons of different biological ancestry. American journal of physical anthropology. 2000;112(1):17-27.
73. Del Bino S, Bernerd F. Variations in skin colour and the biological consequences of ultraviolet radiation exposure. The British journal of dermatology. 2013;169 Suppl 3:33-40.
74. Visscher MO. Skin Color and Pigmentation in Ethnic Skin. Facial plastic surgery clinics of North America. 2017;25(1):119-25.
75. Jouganous J, Long W, Ragsdale AP, Gravel S. Inferring the Joint Demographic History of Multiple Populations: Beyond the Diffusion Approximation. Genetics. 2017;206(3):1549-67.
